# A database of human exposomes and phenomes from the US National Health and Nutrition Examination Survey

**DOI:** 10.1038/sdata.2016.96

**Published:** 2016-10-25

**Authors:** Chirag J. Patel, Nam Pho, Michael McDuffie, Jeremy Easton-Marks, Cartik Kothari, Isaac S. Kohane, Paul Avillach

**Affiliations:** 1 Department of Biomedical Informatics, Harvard Medical School, 10 Shattuck St., Boston, Massachusetts 02115, USA

**Keywords:** Diagnostic markers, Databases, Epidemiology

## Abstract

The National Health and Nutrition Examination Survey (NHANES) is a population survey implemented by the Centers for Disease Control and Prevention (CDC) to monitor the health of the United States whose data is publicly available in hundreds of files. This Data Descriptor describes a single unified and universally accessible data file, merging across 255 separate files and stitching data across 4 surveys, encompassing 41,474 individuals and 1,191 variables. The variables consist of phenotype and environmental exposure information on each individual, specifically (1) demographic information, physical exam results (e.g., height, body mass index), laboratory results (e.g., cholesterol, glucose, and environmental exposures), and (4) questionnaire items. Second, the data descriptor describes a dictionary to enable analysts find variables by category and human-readable description. The datasets are available on DataDryad and a hands-on analytics tutorial is available on GitHub. Through a new big data platform, BD2K *Patient Centered Information Commons* (http://pic-sure.org), we provide a new way to browse the dataset via a web browser (https://nhanes.hms.harvard.edu) and provide application programming interface for programmatic access.

## Background & Summary

United States health agencies, including the United States Centers for Disease Control and Prevention (CDC), have made a significant investment in monitoring the health of the population through surveys such as the National Health and Nutrition Examination Survey (NHANES). These datasets provide individual-level health-related measures in a large and representative sample of the United States (e.g., from 1999–2006, *N*=41,474). For example, these data are instrumental in providing prevalence of disease-related factors, such as diabetes and obesity (e.g., refs 1–3), drug use^4^, and present reference intervals for child growth, such as head circumference. These data have helped to shape public health policy. For example, these data were used to demonstrate the effect of removal of lead from gasoline (a gross decrease since legislation). Many have used these data to create hypotheses regarding associations between biomarkers of environmental chemical factors and disease, such as diabetes and heart disease. We have used these data to perform the first ‘environment-wide association studies’ (EWAS)^5^, linking >250 environmental biomarkers with disease phenotypes, such as diabetes^6,7^, self-reported preterm birth^8^, serum cholesterol levels^9^, blood pressure^10^, all-cause mortality^11^, telomere length^12^, and income^13^.

The NHANES is a CDC program that began in the 1960 s and in the current day, bi-annually samples 15 counties of United States population (N~5 K per year). Each year, the counties that are sampled change, ensuring a representative and diverse sampling. Specifically, NHANES uses a multistage and ‘probability’ sampling design. To provide reliable statistics, the NHANES ‘over-samples’ persons 60 and older, African Americans, and Hispanics and analysts.

The NHANES is designed to estimate major disease prevalence, such as diabetes, obesity, and cardiovascular disease in the United States. It is one of the only studies that combines simultaneously assessed self-reported questionnaires and physical measurements. Self-reported instruments include dietary questionnaires the estimate nutrient content of foods consumed around the time of survey and health and disease-related questionnaire. Second, the NHANES contains phenotypes such as blood pressure, pulse rate, respiratory capacity, height, weight, and tooth count in an effort to estimate the range and prevalence of these phenotypic measures. They are not used for medical diagnoses for the participants.

The *exposome* has been touted as the comprehensive battery of environmental exposures encountered in humans^14^. The CDC NHANES is one of the first population survey programs to have *exposome* measurements. The CDC samples urine, blood, and other human tissue to measure environmental exposure indicators of the exposome using gold standard mass spectrometry and immunological assays. Environmental exposure assays include, for example, lead, mercury, arsenic, pesticide metabolites, air pollution indicators, and plasticizing agents, all hypothesized to have some relationship with health. The NHANES has been instrumental in providing what and how many environmental chemicals are found in human tissue (e.g., ref. 15). Clinical and physiological phenotypes of the phenome include cholesterol (e.g., HDL-cholesterol, LDL-cholesterol, triglycerides), glucose, insulin, C-reactive protein (CRP), white blood counts, and other blood or urine based measures. All of the measures are taken simultaneously.

The NHANES raw datasets for surveys currently exist in >250 number of separate proprietary SAS-formatted files (e.g.: https://wwwn.cdc.gov/Nchs/Nhanes/1999-2000/DEMO.XPT). Description of each variable (e.g., a human-readable variable name and units of measurement) exist in a separate table embedded in an.html webpage (e.g.: https://wwwn.cdc.gov/nchs/nhanes/search/variablelist.aspx?Component=Demographics). All technical information about each variable, such as way it was measured, are also available on the NHANES website as a.html page. The NHANES has variables of many types, including biomarkers of environmental exposures, clinical markers, physiological measures, questionnaire items, that are continuous or categorical. Next, the NHANES consists of multiple ‘survey waves’ that represent a sampling for a 2-year period (e.g., 1999–2000 to 2005–2006 and beyond). Our data resource allows investigators move beyond examining a handful of variables to one that takes advantage of the multiple variables across a number of NHANES survey waves (e.g., akin to refs 11,16,17). Second, our data resource allows for quick evaluation of hypotheses before executing a formal scientific investigation. We are offering this integrated resource ready to analyze for free of cost, leveraging our previous experience.

We also offer a way to access the dataset programmatically through an ‘application programming interface’ (API). We utilize *i2b2*/*tranSMART*, a data repository software platform used to implement BD2K *Patient Information Commons-Standardized Unification of Research Elements (PIC-SURE)* (http://pic-sure.org). The *Informatics for Integrating Biology and the Bedside* (*i2b2*) open-source software was developed to provide a federated informatics infrastructure to house/store, maintain, and analyze cohort data emerging from population-level datasets from around the nation for the purpose of driving biological discovery^18–20^. *i2b2* enables the cohesive analysis of heterogeneous phenotypic data. *tranSMART* is an open-sourced ‘application layer’ for *i2b2* (refs 21,22), providing a software add-ons to *i2b2* for user interfaces, data mapping, and loading cohort data. This software provides a means to assemble, query, and analyze disparate and heterogeneous cohort datasets, such as the NHANES. The PIC-SURE software technology provides an accessible representation of NHANES, facilitating ad hoc querying of the health measures of the US while providing an application programming interface (API) for consumption by external applications and scripts, such as statistical tools such as *R*.

In this data descriptor, we provide (1) a data descriptor for unified raw NHANES data, (2) sample starter analytic code, analytic compute environment in a *Docker* container, and guide to conduct analysis with the NHANES data, and (3) introduce the *PIC-SURE* enabled web application to browse and download the data through an ‘application programming interface’ (API). Further, we have provided a web video tutorial on the web application located here: https://vimeo.com/182576739.

We emphasize our data descriptor is an introduction for use of the NHANES dataset and that all analyses must be verified with data from CDC/NHANES directly. Furthermore, we also emphasize that the derived variables we include were suitable for our own analyses of NHANES and may not be suitable for hypotheses specific to other investigators. Therefore, we include all raw variables in our integrated dataset for investigators.

## Methods

### National Health and Nutrition Examination Surveys (NHANES) data

NHANES datasets are publicly accessible through the United States Centers of Disease Control and Prevention (US CDC)^23–26^. All NHANES participants have consented for their information to be used in research.

**Figure 1:**
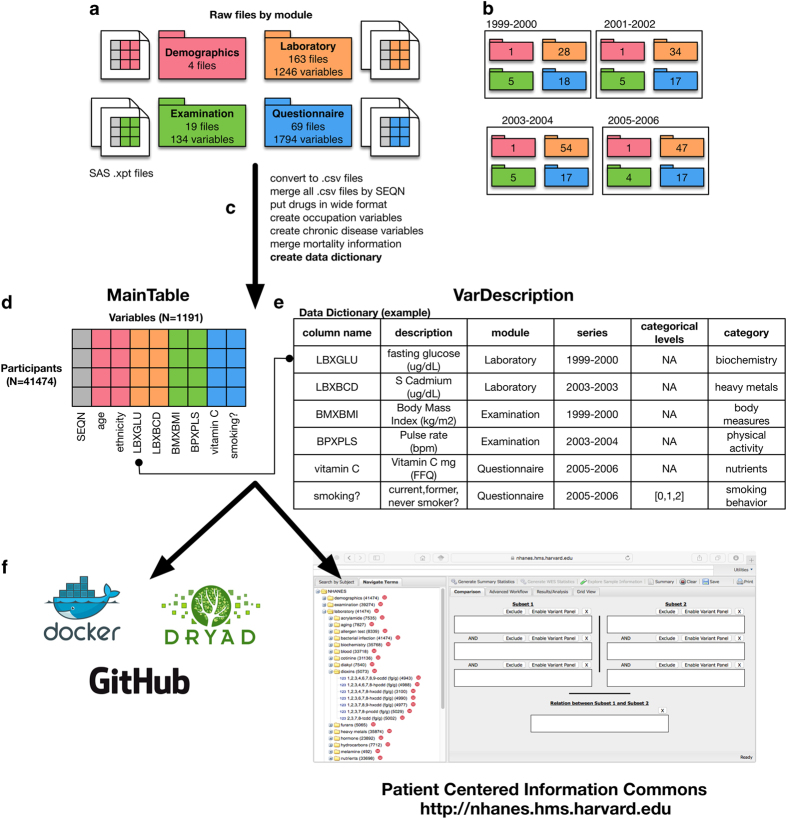
Methods overview for creating the unified NHANES dataset. (**a**) Each SAS-formatted (.xpt) data file provided by the CDC/NHANES are binned by ‘module’ (represented by folders), including Demographics (4 files), Laboratory (163 files), Examination (19 files), and Questionnaire (69 files). Participant identifiers to merge data files across modules are depicted as gray colums. (**b**) File number breakdown by survey year and module. (**c**) We processed the data to create new variables, added pharmaceutical drug information, and added mortality information. (**d**) We merged all 255 files by the patient identifier to create a large unified table (‘MainTable’) consisting of 41 K participants and 1191 unique variables. (**e**) We created a data dictionary that contains human readable variable descriptions and other meta-data, such as variable category and the levels of the variable if categorical. (**f**) Data is accessible via DataDryad and browsable through the PIC-SURE website (https://nhanes.hms.harvard.edu). Data and a Usage Guide is available on GitHub. Rstudio analytics environment with dataset, xwas R library, and user guides packaged as a Docker hub container (chiragjp/nhanes_scidata).

Figure 1 shows our procedure. We downloaded 255 total data files, encoded in proprietary SAS ‘.xpt’ format, corresponding to participants surveyed in 1999–2000 (52 files), 2001–2002 (57 files), 2003–2004 (77 files), 2005–2006 (69 files) from the CDC NHANES website (Fig. 1a,b) which are hyperlinked to a CDC website in January 2014. We chose to focus on these surveys as they had the greatest number of variables available at the time of download. We will make future instances of merged NHANES available via DataDryad with additional Data Descriptors.

Each participant of the NHANES has a unique identifier; in other words, there is no overlap in participants in the 1999–2000, 2001–2002, 2003–2004, and 2005–2006 surveys. In total, these 255 files contain information on 41,474 distinct individuals representative of the United States population and 1,191 unique variables.

Each.xpt formatted data file consists of information structured in a ‘N×M’ form, in which N number of individuals make up every row and M number of columns of variables for each individual (Fig. 1a,b) and a participant identifier (called ‘SEQN’), the primary key that joins the data files together (shown as a gray column, Fig. 1a). The CDC/NHANES have binned each file in 4 separate ‘modules’ that corresponded to (1) whether they contain demographic information (e.g., age, race/ethnicity, survey characteristics, income [Fig. 1a, red folder]), (2) laboratory measures (e.g., biomarker measurements assayed in biological tissue, such as serum or urine, depicted in orange [Fig. 1a, orange folder]), (3) physical examination (e.g., measurements such as body mass index, weight, height; [Fig. 1a, green folder]), or questionnaire (e.g., food-frequency questionnaire or health status questionnaire [Fig. 1a, blue folder]). Each of these categories, or ‘modules’, are called Demographics, Laboratory, Examination, and/or Questionnaire modules respectively.

In total, we downloaded 4 Demographics data files, 163 Laboratory data files, 19 Examination files, and 69 Questionnaire data files. Fig. 1b and Table 1 depicts the total files for each NHANES module for the 1999–2000, 2001–2002, 2003–2004, and 2005–2006 datasets.

After downloading all 255.xpt files, we executed a number of data processing steps. First, all.xpt files were converted into.csv files using using the ‘foreign’ R package^27^, preserving the original ‘N×M’ form of the data. Next, we created some derived variables to ease potential downstream analyses, including (1) occupation (1 variable), (2) chronic disease (40 variables), and (3) pharmaceutical drug use (100 variables) (Fig. 1c).

We coded occupation as variables that correspond to (1) white-collar and professional jobs that are coded as white-collar and semi-routine (e.g., technicians), blue-collar and high-skill (e.g., mechanics, construction trades, and military), blue-collar and semi-routine (e.g., personal services, farm workers) as previously described in our previous EWAS^28^. Labor force participation was defined as working at a job or business or having a job or business within the last two weeks, not including work around the house.

We defined presence of 6 types of chronic diseases, including diabetes (1 variable), coronary disease (1 variable), hypertension (1 variable), asthma (1 variable), rheumatoid arthritis, osteoarthritis, and 30 site-specific cancers. We coded diabetes as present (as an integer 1) if the participant had a fasting blood glucose greater than 125 mg/dl (as per American Diabetes Association [ADA]) threshold for diabetes diagnosis or if the participant answered ‘yes’ to the question, ‘Other than during pregnancy, have you ever been told by a doctor or health professional that {you have/{he/she/SP} has} diabetes or sugar diabetes?’. If the participant did not have both of those characteristics, he/she were coded as 0 (ref. 29). Similarly, we defined presence of hypertension as 1 if the participant had a systolic over diastolic blood pressure greater than 130 over 90 or answered ‘yes’ to the question, ‘Have you ever been told by a doctor or other health professional that you had hypertension, also called high blood pressure’ and 0 otherwise. We defined presence of coronary disease as 1 if the participant answered ‘yes’ to the question, ‘Has a doctor or other health professional ever told you that you had coronary (kor-o-nare-ee) heart disease?’ and 0 otherwise. The NHANES also contains coding for site-specific cancers. First, participants were asked whether a doctor has ‘ever told you you have cancer?’. If the participant replies yes to a question, a followup question is administered, ‘what type of cancer do you have’ and the participant can answer from a set of 27 cancers, such as breast, skin, lung, colon, bladder, kidney, and other type of cancers. We turned these into 27 separate variables that are coded 1 if the site-specific cancer is present, 0 otherwise.

Third, we extracted pharmaceutical drug use for each participant. The CDC used a Master Drug Database (MDDB), a proprietary but comprehensive database of all prescription and some nonprescription drug products available in the U.S. drug market. The CDC NHANES interviewer asked participants whether they were taking a drug in the past month, and if they were, what drugs they were taking. The CDC NHANES interviewer matched each drug to an MDDB identifier and drug description (e.g., METFORMIN or ALBUTEROL). Second, the CDC NHANES interviewer—if the interview was occurring at the participant’s home—verified possession of the prescription drug container. Each participant could report taking more than one drug. There were 626, 668, 667, and 692 unique drugs found by the CDC interviewers in the 1999–2000, 2001–2002, 2003–200, and 2005–2006 cohorts respectively. To keep the merged data table (Fig. 1d) of tractable size, we chose to focus on the top 100 drugs that were most prevalent in the population. We coded a participant was on a drug if (1) they reported use of a drug and (2) whether the interviewer verified the container was present.

The CDC also ascertained cause and time of death (mortality) information for a subset of the participants in 2006 by linking eligible participants to the National Death Index. We incorporated this data into our data merge (*n*=11,429 participants). The variables that describe the mortality information include ELIGSTAT (whether the person was eligible for death linkage), MORTSTAT (whether the participant was deceased), PERMTH_INT (time to death from interview or time to linkage if participant is living [censored data]), PERMTH_EXM (time to death from examination or time to linkage if participant if living), DIABETES (if the cause of death was diabetes), HYPERTEN (if the cause of death was hypertension, and HIPFRACT (if the cause of death was hip fracture).

Finally, we combined the 255 files together into single data file by merging by the patient identifier (‘SEQN’) (Fig. 1d). This merge resulted in one consolidated and analysis-ready data file representing a grand total of 1,191 variables on 41,474 participants.

### Creation of a digital handbook: annotating and categorizing the NHANES datasets

The CDC NHANES have provided a.html formatted codebook (e.g.: https://wwwn.cdc.gov/Nchs/Nhanes/Search/variablelist.aspx?Component=Laboratory&CycleBeginYear=1999) that consists of variable name (column in the.xpt file) and a human-readable description of each variable. For example, the variable with names RIDAGEYR or LBXGLU is described as ‘Age in Years’ and ‘fasting serum glucose [mgul^−1^]’ respectively. These descriptions include the variable units, such as ‘ug/mL’ (inferred as a continuous variable), or ‘positive’/‘negative’ (a binary variable) of each variable.

We have extended the CDC NHANES data description methodology in the following ways (Fig. 1e) to facilitate analysis and data browsing. Specifically, we have created a data dictionary that contains the name of the variable, a human readable description of the variable, what ‘module’ a variable belongs to, what survey the variable was measured (e.g., 1999–2000). Second, we have binned each variable into categories that offer more specificity than the CDC NHANES ‘module’ characterization. We make available the data dictionary (Fig. 1e) along with the data set (Data Citation 1). A summary of the number of variables per category, the median sample size for the variables in the category, and the demographic representation (percent female and race/ethnicity available for each variable) in Table 2. The entire data dictionary is available as Table 3 (available online only) (Data Citation 1 and Table 3 (available online only)).

These categories aide in the filtering and querying of variables with common types, such as ‘nutrients’, ’body measures’, ‘pharmaceutical drug’, ‘viral infection’, and ‘pesticides’. Second, we have created a column that denotes the categorical levels for variables that are categorical or binary. For example, ‘Are you a past, current, or never smoker?’ is a variable that has three levels, one representing ‘never smoker’, ‘current smoker’, and ‘past smoker’; these categories are captured in a column called ‘categorical levels’.

### Browsing and accessing the data through BD2K Patient-Centered Information Commons (PIC)

**Figure 2:**
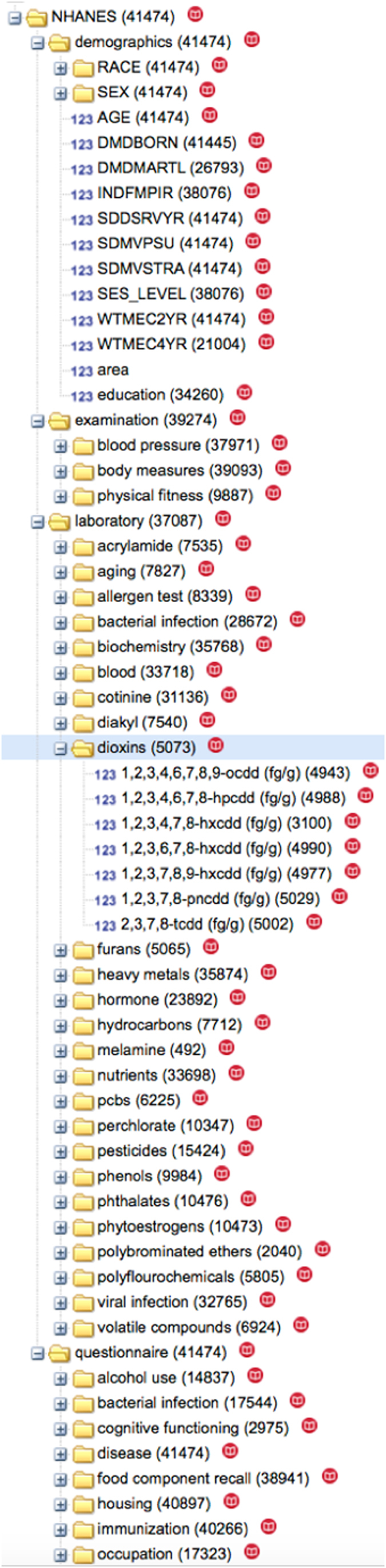
Screenshot of NHANES data hierarchy displayed in the PIC data browser tool. Variables are shown with sample sizes. Highlighted in the screen shot are all laboratory measures of dioxins, a type of environmental exposure assayed in serum.

**Figure 3:**
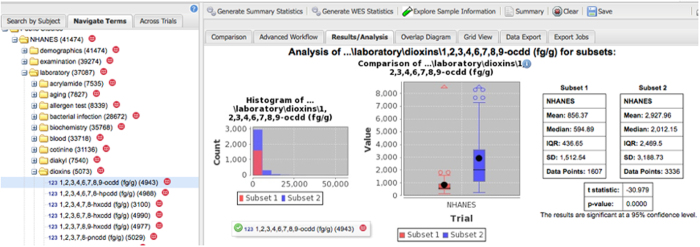
Screenshot of Drag-and-drop example to explore the NHANES datasets (left) using the PIC cohort browser tool. A comparison of raw Dioxin (1-9-ocdd) levels by age groups. Red, age <25 y; blue, age ≥25 y.

We leveraged the Patient-Centered Information Commons (PIC, for an overview, see: http://pic-sure.org)) platform is leveraged to (1) enable interactive web browsing of the NHANES data (see: https://nhanes.hms.harvard.edu) and (2) access data through an application programming interface (API). PICs are built using the i2b2/tranSMART software stack. Data is organized into a hierarchy resembling a directory structure to facilitate browsing (Figs 2 and 3). Raw data can be also queried using a drag and drop interface (Fig. 3). With the NHANES, we organized each of the 1171 variables into a multi-level hierarchy that was ordered by the module (*i.e.*, ‘Laboratory’, ‘Examination’, ‘Demographics’, and ‘Questionnaire’) and category (*i.e.*, ‘pesticides’, ‘body measures’, etc, see Table 2). To display this NHANES data hierarchy in our user interface we created a Metadata mapping file located here: https://github.com/hms-dbmi/public-data-deployments/blob/master/NHANES/nhanes_9906.map and used this mapping file to integrate the data file.

The merged dataset (‘MainTable’) and data dictionary (‘VarDescription’) (Fig. 1d,e) are made available in DataDryad (Fig. 1f). A Usage Guide and.Rdata files are provided for download in GitHub (Fig. 1f). Finally, all data are browsable at https://nhanes.hms.harvard.edu.

We have provided two additional resources for individuals to learn about the resource. The first is a tutorial of the web application located at Vimeo (https://vimeo.com/182576739). This web application shows users how to count the number of variables and number of participants (by age, sex, and race/ethnicity) that we believe will aid in planning analyses of the data. Second, we have built an online course (http://www.chiragjpgroup.org/exposome-analytics-course/) to guide users step-by-step through an investigation our group recently published (Patel *et al.*, 2016).

We plan to assess how frequently our data descriptor and data resources are being utilized by the scientific community through traditional means (e.g., number of citations to this descriptor), but also through by counting the number of unique visitors to the Vimeo video website, the web application (http://nhanes.hms.harvard.edu), and through feedback from course materials.

### Code availability

We demonstrate 3 use-cases in using the integrated NHANES datasets in a R markdown source file (see ‘Usage Notes’). Code is available on GitHub here: https://github.com/chiragjp/nhanes_scidata. One other example using our API access is available here: https://github.com/hms-dbmi/R-IRCT/blob/master/Example_NHANES.Rmd

*I2b2/TranSMART software stack.* Code to implement a PIC is open-sourced and available here: https://github.com/hms-dbmi/HMS-DBMI-transmartApp

## Data Records

Data record 1: Integrated NHANES dataset and data dictionary in.csv format.

The integrated NHANES dataset and a data dictionary is available online at Dryad (Data Citation 1) as a .zip file which includes 3 .csv formatted files. The first file (‘data file’) contains each individual (as rows) surveyed in 1999–2006 with all of their measurements (as columns) (‘MainTable’, Fig. 1d). The second file contains a data dictionary file which contains the name of the variable as represented in the data file, a human readable description of the variable, the categories that the variable belongs to), and the levels of the categories (if a categorical variable) (Fig. 1e). The third file is a dictionary specifically for demographic information, such as describing the columns for age, sex, race/ethnicity, whether the participant was born in the US, education level, income level, and mortality information. Also, to facilitate analyses using the *R* programming language, we have provided a 4th file that contains all the files described above as a *R* data object in.Rdata format.

## Technical Validation

The raw data contained herein are from the CDC NHANES. The CDC NHANES have performed extensive technical validation of their data described elsewhere (e.g., refs 30,31).

## Usage Notes

The NHANES utilizes a ‘multistage survey sampled’ study design to ensure minority subgroups (e.g., Blacks, Mexican-American, elderly, pre-adolescents) of the population are appropriately represented in the dataset^32^ and to optimize sampling resources. Therefore, statistical analyses need to take into account the structure of the sampling into account to provide accurate estimates of the population, such as means, standard errors, and correlations^33^.

To demonstrate how to properly analyse NHANES data, we provide a *R* markdown files in our GitHub repository (https://github.com/chiragjp/nhanes_scidata) to re-create several relevant analyses.

### Conducting an ‘environment-wide association analysis’ in all-cause mortality in NHANES

Previously, we conducted a data-driven search of environmental exposure factors associated with all-cause mortality known as an ‘environment-wide association study’^28^. In the guide (https://github.com/chiragjp/nhanes_scidata/blob/master/User_Guide.Rmd), we describe how to associate one of the top findings, serum cadmium, with all-cause mortality using survey-weighted Cox proportional hazards regression.

### Distribution of serum lead in in children: Accessing the NHANES in PIC-SURE API

In this guide (https://github.com/chiragjp/nhanes_scidata/blob/master/User_Guide_PIC.Rmd), we demonstrate how to access the NHANES data programmatically through the PIC-SURE API. In our example, we show how to query the API to estimate the quartiles of serum lead in the US population of all ages and aged under 18.

### Redistributable analytics environment in Docker

The issue of reproducibility, replicability, and scalability in computational scientific research has been raised on multiple occasions^34,35^. We promote a reproducible practice by packaging the curated NHANES data (Data Citation 1) with an analytics environment comprised of R-3.3.0 (ref. 36) and the Rstudio-0.99.902 (ref. 37) web interface in addition to a custom R library for regression studies in a Docker container^38^. The packaged environment is publically available on Docker hub (https://hub.docker.com/r/chiragjp/nhanes_scidata/) and can be consistently deployed across local or cloud-based environments. We have provided these materials as a hands-on short course available here: http://www.chiragjpgroup.org/exposome-analytics-course/

## Additional Information

**How to cite**: Patel, C. J. *et al.* A database of human exposomes and phenomes from the US National Health and Nutrition Examination Survey. *Sci. Data* 3:160096 doi: 10.1038/sdata.2016.96 (2016).

**Publisher’s note**: Springer Nature remains neutral with regard to jurisdictional claims in published maps and institutional affiliations.

## Supplementary Material



## Figures and Tables

**Table 1:** Number of variables and files per NHANES module.

**Description**	**NHANES module**	**Number of variables**	**Number of files**
Physical examination	Examination	134	19
Laboratory assay (serum and urine) results	Laboratory	1,246	163
Self-reported questionnaire items	Questionnaire	1,794	69
Demographic attributes and cause of death in 2006	Demographics and mortality	28	4

**Table 2:** Categories of variables, the number of variables, surveys represented (1=1999–2000, 2=2001–2002,3=2003–2004,4=2005–2006) number of raw data files, sample size, and demographic distribution.

**Category**	**Number of variables**	**Surveys**	**Median (N)**	**Female (%)**	**White (%)**	**Black (%)**	**Mexican (%)**	**Other his (%)**	**Other eth (%)**
acrylamide	2	3	7189.50	0.51	0.41	0.26	0.25	0.03	0.04
aging	1	1;2	7827.00	0.52	0.51	0.17	0.24	0.05	0.03
alcohol use	4	1;2;3;4	11141.50	0.46	0.54	0.17	0.21	0.04	0.03
allergen test	20	4	7796.50	0.51	0.40	0.26	0.26	0.03	0.05
bacterial infection	48	1;2;3;4	742.00	0.50	0.43	0.26	0.24	0.04	0.04
biochemistry	56	1;2;3;4	26038.00	0.51	0.43	0.23	0.26	0.04	0.04
blood	20	1;2;3;4	33661.00	0.51	0.39	0.25	0.28	0.04	0.04
blood pressure	4	1;2;3;4	26036.00	0.51	0.39	0.25	0.28	0.04	0.04
body measures	19	1;2;3;4	27259.00	0.48	0.40	0.25	0.27	0.04	0.04
cognitive functioning	2	1;2	2975.00	0.52	0.61	0.14	0.19	0.04	0.02
cotinine	1	1;2;3;4	31136.00	0.51	0.40	0.25	0.27	0.04	0.04
diakyl	7	1;2;3	7422.00	0.52	0.39	0.25	0.27	0.04	0.04
dioxins	7	1;2;3	4988.00	0.52	0.44	0.21	0.26	0.04	0.04
disease	40	1;2;3;4	18526.50	0.53	0.48	0.21	0.23	0.04	0.04
food component recall	162	1;2;3;4	16412.00	0.51	0.39	0.25	0.26	0.04	0.04
furans	10	1;2;3	4980.00	0.52	0.44	0.21	0.26	0.04	0.04
heavy metals	31	1;2;3;4	10081.00	0.50	0.39	0.26	0.27	0.04	0.04
hormone	8	1;2;3;4	9473.00	0.52	0.42	0.22	0.26	0.04	0.04
housing	9	1;2;3;4	35087.00	0.51	0.39	0.25	0.28	0.04	0.05
hydrocarbons	23	1;2;3	7209.00	0.52	0.41	0.25	0.27	0.04	0.04
immunization	3	1;2;3;4	35305.00	0.52	0.40	0.25	0.27	0.04	0.04
melamine	1	3	492.00	0.53	0.42	0.27	0.23	0.04	0.04
nutrients	31	1;2;3;4	22880.00	0.51	0.42	0.25	0.25	0.04	0.04
occupation	21	1;2;3;4	769.00	0.27	0.46	0.20	0.27	0.05	0.04
pcbs	38	1;2;3	6049.00	0.52	0.43	0.22	0.27	0.04	0.04
perchlorate	7	3;4	5479.50	0.51	0.41	0.26	0.25	0.03	0.05
pesticides	66	1;2;3;4	4999.00	0.52	0.39	0.25	0.27	0.04	0.04
pharmaceutical	221	1;2;3;4	20456.00	0.51	0.39	0.25	0.28	0.04	0.04
phenols	7	3;4	5065.00	0.51	0.42	0.26	0.25	0.03	0.05
phthalates	15	1;2;3;4	10476.00	0.51	0.40	0.25	0.27	0.04	0.04
physical fitness	15	1;2;3;4	8688.00	0.48	0.34	0.26	0.32	0.04	0.04
phytoestrogens	6	1;2;3;4	10453.50	0.51	0.40	0.25	0.27	0.04	0.04
polybrominated ethers	12	3	1999.50	0.51	0.45	0.24	0.24	0.03	0.04
polyflourochemicals	12	1;3;4	5805.00	0.51	0.42	0.24	0.27	0.04	0.03
sexual behavior	2	1;2;3;4	5178.00	0.00	0.48	0.21	0.22	0.04	0.04
smoking behavior	30	1;2;3;4	7479.50	0.48	0.51	0.20	0.20	0.04	0.04
smoking family	8	1;2;3;4	7668.00	0.49	0.43	0.35	0.15	0.04	0.04
social support	3	1;2;3;4	9937.00	0.51	0.56	0.19	0.18	0.03	0.03
street drug	24	1;2;3;4	600.00	0.39	0.54	0.20	0.20	0.04	0.04
sun exposure	1	3;4	2444.00	0.50	0.70	0.06	0.17	0.02	0.04
supplement use	85	1;2;3;4	41366.00	0.51	0.39	0.25	0.28	0.04	0.04
viral infection	18	1;2;3;4	15400.00	0.51	0.40	0.24	0.27	0.04	0.04
volatile compounds	51	1;2;3;4	5573.00	0.53	0.45	0.24	0.23	0.04	0.05
Sample sizes are the median participants available for the variables in the respective categories (e.g., the median sample size available for all the alcohol use variables is 11,141.5). ‘Other His’ denotes ‘Other Hispanic’. ‘Other Eth’ denotes ‘Other race/ethnicity’.									

**Table 3:** Comprehensive data dictionary

**var**	**var_desc**	**series**	**module**	**category**	**categorical_levels**
LBXHBC	Hepatitis B core antibody	2003-2004	laboratory	viral infection	NA
LBDHBG	Hepatitis B surface antigen	2003-2004	laboratory	viral infection	NA
LBDHCV	Hepatitis C antibody (confirmed)	2003-2004	laboratory	viral infection	NA
LBDHD	Hepatitis D (anti-HDV)	2003-2004	laboratory	viral infection	NA
LBXHBS	Hepatitis B Surface Antibody	2003-2004	laboratory	viral infection	NA
LBXHA	Hepatitis A Antibody (Anti-HAV)	2003-2004	laboratory	viral infection	NA
LBDHI	HIV antibody test result	2003-2004	laboratory	viral infection	NA
LBXCD4	CD4 counts (cells/mm3)	2003-2004	laboratory	viral infection	NA
LBXCD8	CD8 counts (cells/mm3)	2003-2004	laboratory	viral infection	NA
URXUP8	Perchlorate, urine (ng/mL)	2003-2004	laboratory	perchlorate	NA
URXUP8CA	Urine perchlorate (ug per g creatinine)	2003-2004	laboratory	perchlorate	NA
LBXWBF	Water Bromoform (ng/mL)	2003-2004	laboratory	volatile compounds	NA
LBXWCF	Water Chloroform (ng/mL)	2003-2004	laboratory	volatile compounds	NA
LBXWBM	Water Bromodichloromethane (ng/mL)	2003-2004	laboratory	volatile compounds	NA
LBXWCM	Water Dibromochloromethane (ng/mL)	2003-2004	laboratory	volatile compounds	NA
LBXWME	Water MTBE (ng/mL)	2003-2004	laboratory	volatile compounds	NA
LBXV1A	Blood 1,1-Dichloroethane (ng/mL)	2003-2004	laboratory	volatile compounds	NA
LBXV1D	Blood 1,2-Dichlorobenzene (ng/mL)	2003-2004	laboratory	volatile compounds	NA
LBXV1E	Blood 1,1-Dichloroethene (ng/mL)	2003-2004	laboratory	volatile compounds	NA
LBXV2A	Blood 1,2-Dichloroethane (ng/mL)	2003-2004	laboratory	volatile compounds	NA
LBXV2C	Blood cis-1,2-Dichloroethene (ng/mL)	2003-2004	laboratory	volatile compounds	NA
LBXV2T	Blood trans-1,2-Dichloroethene (ng/mL)	2003-2004	laboratory	volatile compounds	NA
LBXV3B	Blood 1,3-Dichlorobenzene (ng/mL)	2003-2004	laboratory	volatile compounds	NA
LBXV4A	Blood 1,1,2-Trichloroethane (ng/mL)	2003-2004	laboratory	volatile compounds	NA
LBXV4C	Blood Tetrachloroethene (ng/mL)	2003-2004	laboratory	volatile compounds	NA
LBXV4T	Blood 1,1,2,2-Tetrachloroethane (ng/mL)	2003-2004	laboratory	volatile compounds	NA
LBXVBF	Blood Bromoform (pg/mL)	2003-2004	laboratory	volatile compounds	NA
LBXVBM	Blood Bromodichloromethane (pg/mL)	2003-2004	laboratory	volatile compounds	NA
LBXVBZ	Blood Benzene (ng/mL)	2003-2004	laboratory	volatile compounds	NA
LBXVCB	Blood Chlorobenzene (ng/mL)	2003-2004	laboratory	volatile compounds	NA
LBXVCF	Blood Chloroform (pg/mL)	2003-2004	laboratory	volatile compounds	NA
LBXVCM	Blood Dibromochloromethane (pg/mL)	2003-2004	laboratory	volatile compounds	NA
LBXVCT	Blood Carbon Tetrachloride (ng/mL)	2003-2004	laboratory	volatile compounds	NA
LBXVDB	Blood 1,4-Dichlorobenzene (ng/mL)	2003-2004	laboratory	volatile compounds	NA
LBXVDM	Blood Dibromomethane (ng/mL)	2003-2004	laboratory	volatile compounds	NA
LBXVDP	Blood 1,2-Dichloropropane (ng/mL)	2003-2004	laboratory	volatile compounds	NA
LBXVEB	Blood Ethylbenzene (ng/mL)	2003-2004	laboratory	volatile compounds	NA
LBXVHE	Blood Hexachloroethane (ng/mL)	2003-2004	laboratory	volatile compounds	NA
LBXVMC	Blood Methylene Chloride (ng/mL)	2003-2004	laboratory	volatile compounds	NA
LBXVME	Blood MTBE (pg/mL)	2003-2004	laboratory	volatile compounds	NA
LBXVOX	Blood o-Xylene (ng/mL)	2003-2004	laboratory	volatile compounds	NA
LBXVST	Blood Styrene (ng/mL)	2003-2004	laboratory	volatile compounds	NA
LBXVTC	Blood Trichloroethene (ng/mL)	2003-2004	laboratory	volatile compounds	NA
LBXV3A	Blood 1,1,1-Trichloroethane (ng/mL)	2003-2004	laboratory	volatile compounds	NA
LBXVTO	Blood Toluene (ng/mL)	2003-2004	laboratory	volatile compounds	NA
LBXVXY	Blood m-/p-Xylene (ng/mL)	2003-2004	laboratory	volatile compounds	NA
LBX2DF	Blood 2,5-Dimethylfuran (ng/mL)	2003-2004	laboratory	volatile compounds	NA
LBXV2P	Blood 1,2-Dibromo-3-chloropropane(ng/mL)	2003-2004	laboratory	volatile compounds	NA
LBXVNB	Blood Nitrobenezene (ng/mL)	2003-2004	laboratory	volatile compounds	NA
URXUCL	Urinary Chlamydia	2003-2004	laboratory	bacterial infection	NA
URXUGC	Urinary Gonorrhea	2003-2004	laboratory	bacterial infection	NA
LBXBCD	Cadmium (ug/L)	2003-2004	laboratory	heavy metals	NA
LBXBPB	Lead (ug/dL)	2003-2004	laboratory	heavy metals	NA
LBXTHG	Mercury, total (ug/L)	2003-2004	laboratory	heavy metals	NA
LBXIHG	Mercury, inorganic (ug/L)	2003-2004	laboratory	heavy metals	NA
LBXCOT	Cotinine (ng/mL)	2003-2004	laboratory	cotinine	NA
URXUBA	Barium, urine (ng/mL)	2003-2004	laboratory	heavy metals	NA
URXUBE	Beryllium, urine (ng/mL)	2003-2004	laboratory	heavy metals	NA
URXUCD	Cadmium, urine (ng/mL)	2003-2004	laboratory	heavy metals	NA
URXUCO	Cobalt, urine (ng/mL)	2003-2004	laboratory	heavy metals	NA
URXUCS	Cesium, urine (ng/mL)	2003-2004	laboratory	heavy metals	NA
URXUMO	Molybdenum, urine (ng/mL)	2003-2004	laboratory	heavy metals	NA
URXUPB	Lead, urine (ng/mL)	2003-2004	laboratory	heavy metals	NA
URXUPT	Platinum, urine (ng/mL)	2003-2004	laboratory	heavy metals	NA
URXUSB	Antimony, urine (ng/mL)	2003-2004	laboratory	heavy metals	NA
URXUTL	Thallium, urine (ng/mL)	2003-2004	laboratory	heavy metals	NA
URXUTU	Tungsten, urine (ng/mL)	2003-2004	laboratory	heavy metals	NA
URXUUR	Uranium, urine (ng/mL)	2003-2004	laboratory	heavy metals	NA
LBXHCY	Homocysteine (umol/L)	2003-2004	laboratory	biochemistry	NA
LBXMMA	Methylmalonic acid (umol/L)	2003-2004	laboratory	biochemistry	NA
LBXRBF	Folate, RBC (ng/mL RBC)	2003-2004	laboratory	nutrients	NA
LBXB12	Vitamin B12, serum (pg/mL)	2003-2004	laboratory	nutrients	NA
LBXFOL	Folate, serum (ng/mL)	2003-2004	laboratory	nutrients	NA
LBXTFR	Transferrin receptor (mg/L)	2003-2004	laboratory	biochemistry	NA
LBXFER	Ferritin(ng/mL)	2003-2004	laboratory	biochemistry	NA
URXUAS	Urinary total Arsenic (ug/L)	2003-2004	laboratory	heavy metals	NA
URXUAS3	Urinary Arsenous acid (ug/L)	2003-2004	laboratory	heavy metals	NA
URXUAS5	Urinary Arsenic acid (ug/L)	2003-2004	laboratory	heavy metals	NA
URXUAB	Urinary Arsenobetaine (ug/L)	2003-2004	laboratory	heavy metals	NA
URXUAC	Urinary Arsenocholine (ug/L)	2003-2004	laboratory	heavy metals	NA
URXUDMA	Urinary Dimethylarsonic acid (ug/L)	2003-2004	laboratory	heavy metals	NA
URXUMMA	Urinary Monomethylacrsonic acid (ug/L)	2003-2004	laboratory	heavy metals	NA
URXUTM	Urinary Trimethylarsine Oxide (ug/L)	2003-2004	laboratory	heavy metals	NA
URXUHG	Mercury, urine (ng/mL)	2003-2004	laboratory	heavy metals	NA
URXUIO	Iodine, urine (ng/mL)	2003-2004	laboratory	heavy metals	NA
LBXVID	Vitamin D (ng/mL)	2003-2004	laboratory	nutrients	NA
LBXVIC	Vitamin C (mg/dL)	2003-2004	laboratory	nutrients	NA
LBXHE1	Herpes I	2003-2004	laboratory	viral infection	NA
LBXHE2	Herpes II	2003-2004	laboratory	viral infection	NA
LBXGH	Glycohemoglobin (%)	2003-2004	laboratory	biochemistry	NA
LBXGLU	Glucose, plasma (mg/dL)	2003-2004	laboratory	biochemistry	NA
LBXCPSI	C-peptide: SI(nmol/L)	2003-2004	laboratory	biochemistry	NA
LBXIN	Insulin (uU/mL)	2003-2004	laboratory	hormone	NA
LBXCRP	C-reactive protein(mg/dL)	2003-2004	laboratory	biochemistry	NA
LBXBAP	Bone alkaline phosphotase (ug/L)	2003-2004	laboratory	biochemistry	NA
LBXPT21	Parathyroid Hormone(Elecys method) pg/mL	2003-2004	laboratory	hormone	NA
LBXP1	PSA. total (ng/mL)	2003-2004	laboratory	biochemistry	NA
LBXP2	PSA, free (ng/mL)	2003-2004	laboratory	biochemistry	NA
LBDP3	Prostate specific antigen ratio (%)	2003-2004	laboratory	biochemistry	NA
LBXTC	Total Cholesterol (mg/dL)	2003-2004	laboratory	biochemistry	NA
LBDHDD	Direct HDL-Cholesterol (mg/dL)	2003-2004	laboratory	biochemistry	NA
LBXTR	Triglyceride (mg/dL)	2003-2004	laboratory	biochemistry	NA
LBDLDL	LDL-cholesterol (mg/dL)	2003-2004	laboratory	biochemistry	NA
URXUCRSI	Creatinine, urine (umol/L)	2003-2004	laboratory	biochemistry	NA
URXUMA	Albumin, urine (ug/mL)	2003-2004	laboratory	biochemistry	NA
URXUMASI	Albumin, urine (mg/L) SI	2003-2004	laboratory	biochemistry	NA
LBXTO1	Toxoplasma (IgG)	2003-2004	laboratory	bacterial infection	NA
LBXTO2	Toxoplasma (IgM)	2003-2004	laboratory	bacterial infection	NA
LBXME	Measles	2003-2004	laboratory	bacterial infection	NA
LBDRUIU	Rubella international units	2003-2004	laboratory	bacterial infection	NA
LBXVAR	Varicella	2003-2004	laboratory	bacterial infection	NA
LBXDFS	Floor, GFAAS (ug/sq.ft.)	2003-2004	laboratory	heavy metals	NA
LBXDFSF	Floor, FAAS (ug/sq. ft.)	2003-2004	laboratory	heavy metals	NA
LBDDWS	Window, FAAS (ug/sq. ft.)	2003-2004	laboratory	heavy metals	NA
URXBPH	Urinary Bisphenol A (ng/mL)	2003-2004	laboratory	phenols	NA
URXBP3	Urinary Benzophenone-3 (ng/mL)	2003-2004	laboratory	phenols	NA
URX4TO	Urinary 4-tert-octyl phenol (ng/mL)	2003-2004	laboratory	phenols	NA
URXTRS	Urinary Triclosan (ng/mL)	2003-2004	laboratory	pesticides	NA
LBXPFOA	Perfluorooctanoic acid	2003-2004	laboratory	polyflourochemicals	NA
LBXPFOS	Perfluorooctane sulfonic acid	2003-2004	laboratory	polyflourochemicals	NA
LBXPFHS	Perfluorohexane sulfonic acid	2003-2004	laboratory	polyflourochemicals	NA
LBXEPAH	2-(N-ethyl-PFOSA) acetate	2003-2004	laboratory	polyflourochemicals	NA
LBXMPAH	2-(N-methyl-PFOSA) acetate	2003-2004	laboratory	polyflourochemicals	NA
LBXPFDE	Perfluorodecanoic acid	2003-2004	laboratory	polyflourochemicals	NA
LBXPFBS	Perfluorobutane sulfonic acid	2003-2004	laboratory	polyflourochemicals	NA
LBXPFHP	Perfluoroheptanoic acid	2003-2004	laboratory	polyflourochemicals	NA
LBXPFNA	Perfluorononanoic acid	2003-2004	laboratory	polyflourochemicals	NA
LBXPFSA	Perfluorooctane sulfonamide	2003-2004	laboratory	polyflourochemicals	NA
LBXPFUA	Perfluoroundecanoic acid	2003-2004	laboratory	polyflourochemicals	NA
LBXPFDO	Perfluorododecanoic acid	2003-2004	laboratory	polyflourochemicals	NA
URXMBP	Mono-n-butyl phthalate	2003-2004	laboratory	phthalates	NA
URXMCP	Mono-cyclohexyl phthalate	2003-2004	laboratory	phthalates	NA
URXMEP	Mono-ethyl phthalate	2003-2004	laboratory	phthalates	NA
URXMHP	Mono-(2-ethyl)-hexyl phthalate	2003-2004	laboratory	phthalates	NA
URXMNP	Mono-isononyl phthalate	2003-2004	laboratory	phthalates	NA
URXMOP	Mono-n-octyl phthalate	2003-2004	laboratory	phthalates	NA
URXMZP	Mono-benzyl phthalate	2003-2004	laboratory	phthalates	NA
URXMNM	Mono-n-methyl phthalate	2003-2004	laboratory	phthalates	NA
URXMC1	Mono-(3-carboxypropyl) phthalate	2003-2004	laboratory	phthalates	NA
URXMHH	Mono-(2-ethyl-5-hydroxyhexyl) phthalate	2003-2004	laboratory	phthalates	NA
URXMOH	Mono-(2-ethyl-5-oxohexl) phthalate	2003-2004	laboratory	phthalates	NA
URXMIB	Mono-isobutyl phthalate	2003-2004	laboratory	phthalates	NA
URXECP	Mono-2-ethyl-5-carboxypentyl phthalate	2003-2004	laboratory	phthalates	NA
LBXWBCSI	White blood cell count (1000 cells/uL)	2003-2004	laboratory	blood	NA
LBXLYPCT	Lymphocyte percent (%)	2003-2004	laboratory	blood	NA
LBXMOPCT	Monocyte percent (%)	2003-2004	laboratory	blood	NA
LBXNEPCT	Segmented neutrophils percent (%)	2003-2004	laboratory	blood	NA
LBXEOPCT	Eosinophils percent (%)	2003-2004	laboratory	blood	NA
LBXBAPCT	Basophils percent (%)	2003-2004	laboratory	blood	NA
LBDLYMNO	Lymphocyte number	2003-2004	laboratory	blood	NA
LBDMONO	Monocyte number	2003-2004	laboratory	blood	NA
LBDNENO	Segmented neutrophils number	2003-2004	laboratory	blood	NA
LBDEONO	Eosinophils number	2003-2004	laboratory	blood	NA
LBDBANO	Basophils number	2003-2004	laboratory	blood	NA
LBXRBCSI	Red blood cell count (million cells/uL)	2003-2004	laboratory	blood	NA
LBXHGB	Hemoglobin (g/dL)	2003-2004	laboratory	blood	NA
LBXHCT	Hematocrit (%)	2003-2004	laboratory	blood	NA
LBXMCVSI	Mean cell volume (fL)	2003-2004	laboratory	blood	NA
LBXMCHSI	Mean cell hemoglobin (pg)	2003-2004	laboratory	blood	NA
LBXMC	MCHC (g/dL)	2003-2004	laboratory	blood	NA
LBXRDW	Red cell distribution width (%)	2003-2004	laboratory	blood	NA
LBXPLTSI	Platelet count SI (1000 cells/uL)	2003-2004	laboratory	blood	NA
LBXMPSI	Mean platelet volume (fL)	2003-2004	laboratory	blood	NA
URXOP1	Dimethylphosphate (ug/L)	2003-2004	laboratory	diakyl	NA
URXOP2	Diethylphosphate (ug/L)	2003-2004	laboratory	diakyl	NA
URXOP3	Dimethylthiophosphate (ug/L)	2003-2004	laboratory	diakyl	NA
URXOP4	Diethylthiophosphate (ug/L)	2003-2004	laboratory	diakyl	NA
URXOP5	Dimethyldithiophosphate (ug/L)	2003-2004	laboratory	diakyl	NA
URXOP6	Diethyldithiophosphate (ug/L)	2003-2004	laboratory	diakyl	NA
CFDRIGHT	Exercises Correct	2001-2002	questionnaire	cognitive functioning	NA
LBX028	PCB28 (ng/g)	2003-2004	laboratory	pcbs	NA
LBX066	PCB66 (ng/g)	2003-2004	laboratory	pcbs	NA
LBX074	PCB74 (ng/g)	2003-2004	laboratory	pcbs	NA
LBX105	PCB105 (ng/g)	2003-2004	laboratory	pcbs	NA
LBX118	PCB118 (ng/g)	2003-2004	laboratory	pcbs	NA
LBX156	PCB156 (ng/g)	2003-2004	laboratory	pcbs	NA
LBX157	PCB157 (ng/g)	2003-2004	laboratory	pcbs	NA
LBX167	PCB167 (ng/g)	2003-2004	laboratory	pcbs	NA
LBX189	PCB189 (ng/g)	2003-2004	laboratory	pcbs	NA
LBXD01	1,2,3,7,8-pncdd (fg/g)	2003-2004	laboratory	dioxins	NA
LBXD02	1,2,3,4,7,8-hxcdd (fg/g)	2003-2004	laboratory	dioxins	NA
LBXD03	1,2,3,6,7,8-hxcdd (fg/g)	2003-2004	laboratory	dioxins	NA
LBXD04	1,2,3,7,8,9-hxcdd (fg/g)	2003-2004	laboratory	dioxins	NA
LBXD05	1,2,3,4,6,7,8-hpcdd (fg/g)	2003-2004	laboratory	dioxins	NA
LBXD07	1,2,3,4,6,7,8,9-ocdd (fg/g)	2003-2004	laboratory	dioxins	NA
LBXF01	2,3,7,8-tcdf (fg/g)	2003-2004	laboratory	furans	NA
LBXF02	1,2,3,7,8-pncdf (fg/g)	2003-2004	laboratory	furans	NA
LBXF03	2,3,4,7,8-pncdf (fg/g)	2003-2004	laboratory	furans	NA
LBXF04	1,2,3,4,7,8-hxcdf (fg/g)	2003-2004	laboratory	furans	NA
LBXF05	1,2,3,6,7,8-hxcdf (fg/g)	2003-2004	laboratory	furans	NA
LBXF06	1,2,3,7,8,9-hxcdf (fg/g)	2003-2004	laboratory	furans	NA
LBXF07	2,3,4,6,7,8-hxcdf (fg/g)	2003-2004	laboratory	furans	NA
LBXF08	1,2,3,4,6,7,8-hpcdf (fg/g)	2003-2004	laboratory	furans	NA
LBXF09	1,2,3,4,7,8,9-hpcdf (fg/g)	2003-2004	laboratory	furans	NA
LBXF10	1,2,3,4,6,7,8,9-ocdf (fg/g)	2003-2004	laboratory	furans	NA
LBXPCB	3,3,4,4,5-pncb (fg/g)	2003-2004	laboratory	pcbs	NA
LBXTC2	3,4,4,5-tcb (fg/g)	2003-2004	laboratory	pcbs	NA
LBXHXC	3,3,4,4,5,5-hxcb (fg/g)	2003-2004	laboratory	pcbs	NA
LBXTCD	2,3,7,8-tcdd (fg/g)	2003-2004	laboratory	dioxins	NA
LBX044	PCB44 (ng/g)	2003-2004	laboratory	pcbs	NA
LBX049	PCB49 (ng/g)	2003-2004	laboratory	pcbs	NA
LBX052	PCB52 (ng/g)	2003-2004	laboratory	pcbs	NA
LBX087	PCB87 (ng/g)	2003-2004	laboratory	pcbs	NA
LBX099	PCB99 (ng/g)	2003-2004	laboratory	pcbs	NA
LBX101	PCB101 (ng/g)	2003-2004	laboratory	pcbs	NA
LBX110	PCB110 (ng/g)	2003-2004	laboratory	pcbs	NA
LBX128	PCB128 (ng/g)	2003-2004	laboratory	pcbs	NA
LBX138	PCB138 & 158 (ng/g)	2003-2004	laboratory	pcbs	NA
LBX146	PCB146 (ng/g)	2003-2004	laboratory	pcbs	NA
LBX149	PCB149 (ng/g)	2003-2004	laboratory	pcbs	NA
LBX151	PCB151 (ng/g)	2003-2004	laboratory	pcbs	NA
LBX153	PCB153 (ng/g)	2003-2004	laboratory	pcbs	NA
LBX170	PCB170 (ng/g)	2003-2004	laboratory	pcbs	NA
LBX172	PCB172 (ng/g)	2003-2004	laboratory	pcbs	NA
LBX177	PCB177 (ng/g)	2003-2004	laboratory	pcbs	NA
LBX178	PCB178 (ng/g)	2003-2004	laboratory	pcbs	NA
LBX180	PCB180 (ng/g)	2003-2004	laboratory	pcbs	NA
LBX183	PCB183 (ng/g)	2003-2004	laboratory	pcbs	NA
LBX187	PCB187 (ng/g)	2003-2004	laboratory	pcbs	NA
LBX194	PCB194 (ng/g)	2003-2004	laboratory	pcbs	NA
LBX195	PCB195 (ng/g)	2003-2004	laboratory	pcbs	NA
LBX196	PCB196 & 203 (ng/g)	2003-2004	laboratory	pcbs	NA
LBD199	PCB199 (ng/g)	2003-2004	laboratory	pcbs	NA
LBX206	PCB206 (ng/g)	2003-2004	laboratory	pcbs	NA
LBX209	PCB209 (ng/g)	2003-2004	laboratory	pcbs	NA
LBXHCB	Hexachlorobenzene (ng/g)	2003-2004	laboratory	pesticides	NA
LBXBHC	Beta-hexachlorocyclohexane (ng/g)	2003-2004	laboratory	pesticides	NA
LBXGHC	Gamma-hexachlorocyclohexane (ng/g)	2003-2004	laboratory	pesticides	NA
LBXPDE	p,p-DDE (ng/g)	2003-2004	laboratory	pesticides	NA
LBXPDT	p,p-DDT (ng/g)	2003-2004	laboratory	pesticides	NA
LBXODT	o,p-DDT (ng/g)	2003-2004	laboratory	pesticides	NA
LBXOXY	Oxychlordane (ng/g)	2003-2004	laboratory	pesticides	NA
LBXTNA	Trans-nonachlor (ng/g)	2003-2004	laboratory	pesticides	NA
LBXHPE	Heptachlor Epoxide (ng/g)	2003-2004	laboratory	pesticides	NA
LBXMIR	Mirex (ng/g)	2003-2004	laboratory	pesticides	NA
LBXALD	Aldrin (ng/g)	2003-2004	laboratory	pesticides	NA
LBXDIE	Dieldrin (ng/g)	2003-2004	laboratory	pesticides	NA
LBXEND	Endrin (ng/g)	2003-2004	laboratory	pesticides	NA
LBXBB1	2,2,4,4,5,5-hexabromobiphenyl	2003-2004	laboratory	polybrominated ethers	NA
LBXBR1	2,2,4-tribromodiphenyl ether	2003-2004	laboratory	polybrominated ethers	NA
LBXBR2	2,4,4-tribromodiphenyl ether	2003-2004	laboratory	polybrominated ethers	NA
LBXBR3	2,2,4,4-tetrabromodiphenyl ether	2003-2004	laboratory	polybrominated ethers	NA
LBXBR4	2,2,3,4,4-pentabromodiphenyl ether	2003-2004	laboratory	polybrominated ethers	NA
LBXBR5	2,2,4,4,5-pentabromodiphenyl ether	2003-2004	laboratory	polybrominated ethers	NA
LBXBR6	2,2,4,4,6-pentabromodiphenyl ether	2003-2004	laboratory	polybrominated ethers	NA
LBXBR7	2,2,4,4,5,5-hexabromodiphenyl ether	2003-2004	laboratory	polybrominated ethers	NA
LBXBR8	2,2,4,4,5,6-hexabromodiphenyl ether	2003-2004	laboratory	polybrominated ethers	NA
LBXBR9	2,2,3,4,4,5,6-heptabromodiphenyl ethr	2003-2004	laboratory	polybrominated ethers	NA
LBXBR66	2,3,4,4-tetrabromodiphenyl ether	2003-2004	laboratory	polybrominated ethers	NA
LBXBR66L	2,3,4,4-tetrabromodiphenyl lipid adj	2003-2004	laboratory	polybrominated ethers	NA
URXP01	1-napthol (ng/L)	2003-2004	laboratory	hydrocarbons	NA
URXP02	2-napthol (ng/L)	2003-2004	laboratory	hydrocarbons	NA
URXP03	3-fluorene (ng/L)	2003-2004	laboratory	hydrocarbons	NA
URXP04	2-fluorene (ng/L)	2003-2004	laboratory	hydrocarbons	NA
URXP05	3-phenanthrene (ng/L)	2003-2004	laboratory	hydrocarbons	NA
URXP06	1-phenanthrene (ng/L)	2003-2004	laboratory	hydrocarbons	NA
URXP07	2-phenanthrene (ng/L)	2003-2004	laboratory	hydrocarbons	NA
URXP08	1-benzo[c] phenanthrene (ng/L)	2003-2004	laboratory	hydrocarbons	NA
URXP10	1-pyrene (ng/L)	2003-2004	laboratory	hydrocarbons	NA
URXP11	2-benzo[c] phenanthrene (ng/L)	2003-2004	laboratory	hydrocarbons	NA
URXP12	1-benzo[a] anthracene (ng/L)	2003-2004	laboratory	hydrocarbons	NA
URXP13	6-chrysene (ng/L)	2003-2004	laboratory	hydrocarbons	NA
URXP14	3-benzo[c] phenanthrene (ng/L)	2003-2004	laboratory	hydrocarbons	NA
URXP15	3-chrysene (ng/L)	2003-2004	laboratory	hydrocarbons	NA
URXP16	3-benz[a] anthracene (ng/L)	2003-2004	laboratory	hydrocarbons	NA
URXP17	9-fluorene (ng/L)	2003-2004	laboratory	hydrocarbons	NA
URXP19	4-phenanthrene (ng/L)	2003-2004	laboratory	hydrocarbons	NA
URXP20	1-chrysene (ng/L)	2003-2004	laboratory	hydrocarbons	NA
URXP21	2-chrysene (ng/L)	2003-2004	laboratory	hydrocarbons	NA
URXP22	4-chrysene (ng/L)	2003-2004	laboratory	hydrocarbons	NA
URXP24	3-benzo(a) pyrene (ng/L)	2003-2004	laboratory	hydrocarbons	NA
CFDFINSH	Exercises Finished	2001-2002	questionnaire	cognitive functioning	NA
LBXTV	Trichomonas Vaginalis	2003-2004	laboratory	bacterial infection	NA
LBXBV	Bacterial Vaginosis	2003-2004	laboratory	bacterial infection	NA
LBXMS1	S. aureus present 1	2003-2004	laboratory	bacterial infection	NA
LBXM1	MRSA 1	2003-2004	laboratory	bacterial infection	NA
LBXMT1	Tetracycline 1	2003-2004	laboratory	bacterial infection	NA
LBXMZ1	Trimethoprim/Sulfamethoxazole 1	2003-2004	laboratory	bacterial infection	NA
LBXMC1	Clindamycin 1	2003-2004	laboratory	bacterial infection	NA
LBXME1	Erythromycin 1	2003-2004	laboratory	bacterial infection	NA
LBXMP1	Penicillin 1	2003-2004	laboratory	bacterial infection	NA
LBXMO1	Oxacillin 1	2003-2004	laboratory	bacterial infection	NA
LBXMG1	Gentamicin 1	2003-2004	laboratory	bacterial infection	NA
LBXML1	Levofloxacin 1	2003-2004	laboratory	bacterial infection	NA
LBXMR1	Rifampin 1	2003-2004	laboratory	bacterial infection	NA
LBXCD1	Clinda induction (D) test 1	2003-2004	laboratory	bacterial infection	NA
LBXCH1	Chloramphenicol 1	2003-2004	laboratory	bacterial infection	NA
LBXDY1	Daptomycin 1	2003-2004	laboratory	bacterial infection	NA
LBXETA	Enterotoxin A	2003-2004	laboratory	bacterial infection	NA
LBXETB	Enterotoxin B	2003-2004	laboratory	bacterial infection	NA
LBXETC	Enterotoxin C	2003-2004	laboratory	bacterial infection	NA
LBXETD	Enterotoxin D	2003-2004	laboratory	bacterial infection	NA
LBXETH	Enterotoxin H	2003-2004	laboratory	bacterial infection	NA
LBXTSS	Toxic Shock Syndrome Toxin 1	2003-2004	laboratory	bacterial infection	NA
LBXPVL	Panton Valentine Leukocidin	2003-2004	laboratory	bacterial infection	NA
LBXSY1	Syphilis IgG EIA	2003-2004	laboratory	bacterial infection	NA
LBDSY3	Syphilis RPR Titer Level	2003-2004	laboratory	bacterial infection	NA
LBDSY4	Syphilis TP-PA	2003-2004	laboratory	bacterial infection	NA
LBXEPP	Protoporphyrin(ug/dL RBC)	2003-2004	laboratory	biochemistry	NA
LBXSEL	Selenium(ug/L)	2003-2004	laboratory	nutrients	NA
LBXSAL	Albumin (g/dL)	2003-2004	laboratory	biochemistry	NA
LBXSATSI	Alanine aminotransferase ALT (U/L)	2003-2004	laboratory	biochemistry	NA
LBXSASSI	Aspartate aminotransferase AST (U/L)	2003-2004	laboratory	biochemistry	NA
LBXSAPSI	Alkaline phosphotase (U/L)	2003-2004	laboratory	biochemistry	NA
LBXSBU	Blood urea nitrogen (mg/dL)	2003-2004	laboratory	biochemistry	NA
LBXSCA	Total calcium (mg/dL)	2003-2004	laboratory	biochemistry	NA
LBXSCH	Cholesterol (mg/dL)	2003-2004	laboratory	biochemistry	NA
LBXSC3SI	Bicarbonate (mmol/L)	2003-2004	laboratory	biochemistry	NA
LBXSGTSI	Gamma glutamyl transferase (U/L)	2003-2004	laboratory	biochemistry	NA
LBXSGL	Glucose, serum (mg/dL)	2003-2004	laboratory	biochemistry	NA
LBXSIR	Iron, refigerated (ug/dL)	2003-2004	laboratory	biochemistry	NA
LBXSLDSI	Lactate dehydrogenase LDH (U/L)	2003-2004	laboratory	hormone	NA
LBXSPH	Phosphorus (mg/dL)	2003-2004	laboratory	biochemistry	NA
LBXSTB	Total bilirubin (mg/dL)	2003-2004	laboratory	biochemistry	NA
LBXSTP	Total protein (g/dL)	2003-2004	laboratory	biochemistry	NA
LBXSTR	Triglycerides (mg/dL)	2003-2004	laboratory	biochemistry	NA
LBXSUA	Uric acid (mg/dL)	2003-2004	laboratory	biochemistry	NA
LBXSCRINV	1/Creatinine (mg/dL)	2003-2004	laboratory	biochemistry	NA
LBXSNASI	Sodium (mmol/L)	2003-2004	laboratory	biochemistry	NA
LBXSKSI	Potassium (mmol/L)	2003-2004	laboratory	biochemistry	NA
LBXSCLSI	Chloride (mmol/L)	2003-2004	laboratory	biochemistry	NA
LBXSOSSI	Osmolality (mmol/Kg)	2003-2004	laboratory	biochemistry	NA
LBXSGB	Globulin (g/dL)	2003-2004	laboratory	biochemistry	NA
LBXIRN	Iron, Frozen Serum (ug/dL)	2003-2004	laboratory	nutrients	NA
LBXTIB	TIBC, Frozen Serum (ug/dL)	2003-2004	laboratory	biochemistry	NA
LBDPCT	Transferrin saturation (%)	2003-2004	laboratory	biochemistry	NA
LBXVB6	Vitamin B6(Pyridoxal 5-phosphate)nmol/L	2003-2004	laboratory	nutrients	NA
LBXVIE	a-Tocopherol(ug/dL)	2003-2004	laboratory	nutrients	NA
LBDATCSI	a-Tocopherol(umol/L)	2003-2004	laboratory	nutrients	NA
LBXALC	a-Carotene(ug/dL)	2003-2004	laboratory	nutrients	NA
LBXACY	a-Cryptoxanthin(ug/dL)	2003-2004	laboratory	nutrients	NA
LBXBEC	trans-b-carotene(ug/dL)	2003-2004	laboratory	nutrients	NA
LBXBCC	total b-Carotene(ug/dL)	2003-2004	laboratory	nutrients	NA
LBXCBC	cis-b-carotene(ug/dL)	2003-2004	laboratory	nutrients	NA
LBXCLC	cis-Lycopene(ug/dL)	2003-2004	laboratory	nutrients	NA
LBXCLZ	cis- Lutein/Zeaxanthin(ug/dL)	2003-2004	laboratory	nutrients	NA
LBXCRY	b-cryptoxanthin(ug/dL)	2003-2004	laboratory	nutrients	NA
LBXDTC	d-Tocopherol(ug/dL)	2003-2004	laboratory	nutrients	NA
LBXGTC	g-tocopherol(ug/dL)	2003-2004	laboratory	nutrients	NA
LBXLCC	total Lycopene(ug/dL)	2003-2004	laboratory	nutrients	NA
LBXLUT	Lutein(ug/dL)	2003-2004	laboratory	nutrients	NA
LBXLUZ	Combined Lutein/zeaxanthin (ug/dL)	2003-2004	laboratory	nutrients	NA
LBXLYC	trans-lycopene(ug/dL)	2003-2004	laboratory	nutrients	NA
LBXPHF	Phytofluene(ug/dL)	2003-2004	laboratory	nutrients	NA
LBXPHE	Phytoene(ug/dL)	2003-2004	laboratory	nutrients	NA
LBXRPL	Retinyl palmitate(ug/dL)	2003-2004	laboratory	nutrients	NA
LBXRST	Retinyl stearate(ug/dL)	2003-2004	laboratory	nutrients	NA
LBXVIA	Retinol(ug/dL)	2003-2004	laboratory	nutrients	NA
LBXZEA	Zeaxanthin(ug/dL)	2003-2004	laboratory	nutrients	NA
LBXIGE	Serum total IgE antibody (kU/L)	2005-2006	laboratory	allergen test	NA
LBXID2	D. Farinae IgE antibody (kU/L)	2005-2006	laboratory	allergen test	NA
LBXID1	D. Pteronyssinus IgE antibody (kU/L)	2005-2006	laboratory	allergen test	NA
LBXIE1	Cat IgE antibody (kU/L)	2005-2006	laboratory	allergen test	NA
LBXIE5	Dog IgE antibody (kU/L)	2005-2006	laboratory	allergen test	NA
LBXII6	Cockroach IgE antibody (kU/L)	2005-2006	laboratory	allergen test	NA
LBXIM6	Alternaria IgE antibody (kU/L)	2005-2006	laboratory	allergen test	NA
LBXF13	Peanut IgE antibody (kU/L)	2005-2006	laboratory	allergen test	NA
LBXIF1	Egg IgE antibody (kU/L)	2005-2006	laboratory	allergen test	NA
LBXIF2	Milk IgE antibody (kU/L)	2005-2006	laboratory	allergen test	NA
LBXIW1	Ragweed IgE antibody (kU/L)	2005-2006	laboratory	allergen test	NA
LBXIG5	Rye grass IgE antibody (kU/L)	2005-2006	laboratory	allergen test	NA
LBXIG2	Bermuda grass IgE antibody (kU/L)	2005-2006	laboratory	allergen test	NA
LBXIT7	Oak IgE antibody (kU/L)	2005-2006	laboratory	allergen test	NA
LBXIT3	Birch IgE antibody (kU/L)	2005-2006	laboratory	allergen test	NA
LBXF24	Shellfish IgE antibody (kU/L)	2005-2006	laboratory	allergen test	NA
LBXIM3	Aspergillus IgE antibody (kU/L)	2005-2006	laboratory	allergen test	NA
LBXW11	Thistle IgE antibody (kU/L)	2005-2006	laboratory	allergen test	NA
LBXE72	Mouse IgE antibody (kU/L)	2005-2006	laboratory	allergen test	NA
LBXE74	Rat IgE antibody (kU/L)	2005-2006	laboratory	allergen test	NA
URXUMA	Albumin, urine (ug/mL)	2005-2006	laboratory	biochemistry	NA
URXUMS	Albumin, urine (mg/L)	2005-2006	laboratory	biochemistry	NA
URXUCR	Creatinine, urine (mg/dL)	2005-2006	laboratory	biochemistry	NA
URXCRS	Creatinine, urine (umol/L)	2005-2006	laboratory	biochemistry	NA
LBXB12	Vitamin B12 ( pg/mL)	2005-2006	laboratory	nutrients	NA
LBXSAL	Albumin (g/dL)	2005-2006	laboratory	biochemistry	NA
LBXSATSI	Alanine aminotransferase ALT (U/L)	2005-2006	laboratory	biochemistry	NA
LBXSASSI	Aspartate aminotransferase AST (U/L)	2005-2006	laboratory	biochemistry	NA
LBXSAPSI	Alkaline phosphotase (U/L)	2005-2006	laboratory	biochemistry	NA
LBXSBU	Blood urea nitrogen (mg/dL)	2005-2006	laboratory	biochemistry	NA
LBXSCA	Total calcium (mg/dL)	2005-2006	laboratory	biochemistry	NA
LBXSCH	Cholesterol (mg/dL)	2005-2006	laboratory	biochemistry	NA
LBXSC3SI	Bicarbonate (mmol/L)	2005-2006	laboratory	biochemistry	NA
LBXSCRINV	1/Creatinine (mg/dL)	2005-2006	laboratory	biochemistry	NA
LBXSGTSI	Gamma glutamyl transferase (U/L)	2005-2006	laboratory	biochemistry	NA
LBXSGL	Glucose, serum (mg/dL)	2005-2006	laboratory	biochemistry	NA
LBXSIR	Iron, refigerated (ug/dL)	2005-2006	laboratory	biochemistry	NA
LBXSLDSI	Lactate dehydrogenase LDH (U/L)	2005-2006	laboratory	hormone	NA
LBXSPH	Phosphorus (mg/dL)	2005-2006	laboratory	biochemistry	NA
LBXSTB	Total bilirubin (mg/dL)	2005-2006	laboratory	biochemistry	NA
LBXSTP	Total protein (g/dL)	2005-2006	laboratory	biochemistry	NA
LBXSTR	Triglycerides (mg/dL)	2005-2006	laboratory	biochemistry	NA
LBXSUA	Uric acid (mg/dL)	2005-2006	laboratory	biochemistry	NA
LBXSNASI	Sodium (mmol/L)	2005-2006	laboratory	biochemistry	NA
LBXSKSI	Potassium (mmol/L)	2005-2006	laboratory	biochemistry	NA
LBXSCLSI	Chloride (mmol/L)	2005-2006	laboratory	biochemistry	NA
LBXSOSSI	Osmolality (mmol/Kg)	2005-2006	laboratory	biochemistry	NA
LBXSGB	Globulin (g/dL)	2005-2006	laboratory	biochemistry	NA
LBXWBCSI	White blood cell count (1000 cells/uL)	2005-2006	laboratory	blood	NA
LBXLYPCT	Lymphocyte percent (%)	2005-2006	laboratory	blood	NA
LBXMOPCT	Monocyte percent (%)	2005-2006	laboratory	blood	NA
LBXNEPCT	Segmented neutrophils percent (%)	2005-2006	laboratory	blood	NA
LBXEOPCT	Eosinophils percent (%)	2005-2006	laboratory	blood	NA
LBXBAPCT	Basophils percent (%)	2005-2006	laboratory	blood	NA
LBDLYMNO	Lymphocyte number (1000 cells/uL)	2005-2006	laboratory	blood	NA
LBDMONO	Monocyte number (1000 cells/uL)	2005-2006	laboratory	blood	NA
LBDNENO	Segmented neutrophils num (1000 cell/uL)	2005-2006	laboratory	blood	NA
LBDEONO	Eosinophils number (1000 cells/uL)	2005-2006	laboratory	blood	NA
LBDBANO	Basophils number (1000 cells/uL)	2005-2006	laboratory	blood	NA
LBXRBCSI	Red blood cell count (million cells/uL)	2005-2006	laboratory	blood	NA
LBXHGB	Hemoglobin (g/dL)	2005-2006	laboratory	blood	NA
LBXHCT	Hematocrit (%)	2005-2006	laboratory	blood	NA
LBXMCVSI	Mean cell volume (fL)	2005-2006	laboratory	blood	NA
LBXMCHSI	Mean cell hemoglobin (pg)	2005-2006	laboratory	blood	NA
LBXMC	MCHC (g/dL)	2005-2006	laboratory	blood	NA
LBXRDW	Red cell distribution width (%)	2005-2006	laboratory	blood	NA
LBXPLTSI	Platelet count SI (1000 cells/uL)	2005-2006	laboratory	blood	NA
LBXMPSI	Mean platelet volume (fL)	2005-2006	laboratory	blood	NA
URXUCL	Urinary Chlamydia	2005-2006	laboratory	bacterial infection	NA
URXUGC	Urinary Gonorrhea	2005-2006	laboratory	bacterial infection	NA
LBXCOT	Cotinine (ng/mL)	2005-2006	laboratory	cotinine	NA
LBXCRP	C-reactive protein(mg/dL)	2005-2006	laboratory	biochemistry	NA
LBXEPP	Protoporphyrin(ug/dL RBC)	2005-2006	laboratory	biochemistry	NA
LBXFER	Ferritin(ng/mL)	2005-2006	laboratory	biochemistry	NA
LBXIRN	Iron, Frozen Serum (ug/dL)	2005-2006	laboratory	nutrients	NA
LBXTIB	TIBC, Frozen Serum (ug/dL)	2005-2006	laboratory	biochemistry	NA
LBDPCT	Transferrin saturation (%)	2005-2006	laboratory	biochemistry	NA
LBXRBF	Folate, RBC (ng/mL RBC)	2005-2006	laboratory	nutrients	NA
LBXFOL	Folate, serum (ng/mL)	2005-2006	laboratory	nutrients	NA
LBXGH	Glycohemoglobin (%)	2005-2006	laboratory	biochemistry	NA
LBXGLU	Fasting Glucose (mg/dL)	2005-2006	laboratory	biochemistry	NA
LBXIN	Insulin (uU/mL )	2005-2006	laboratory	hormone	NA
PHAFSTHR	Total length of food fast, hours	2005-2006	laboratory	biochemistry	NA
PHAFSTMN	Total length of food fast, minutes	2005-2006	laboratory	biochemistry	NA
LBDHDD	Direct HDL-Cholesterol (mg/dL)	2005-2006	laboratory	biochemistry	NA
LBXHA	Hepatitis A Antibody (Anti-HAV)	2005-2006	laboratory	viral infection	NA
LBXHBS	Hepatitis B Surface Antibody	2005-2006	laboratory	viral infection	NA
LBXHBC	Hepatitis B core antibody	2005-2006	laboratory	viral infection	NA
LBDHBG	Hepatitis B surface antigen	2005-2006	laboratory	viral infection	NA
LBDHD	Hepatitis D (anti-HDV)	2005-2006	laboratory	viral infection	NA
LBDHCV	Hepatitis C antibody (confirmed)	2005-2006	laboratory	viral infection	NA
LBDHI	HIV antibody test result	2005-2006	laboratory	viral infection	NA
LBXHE1	Herpes Simplex Virus I	2005-2006	laboratory	viral infection	NA
LBXHE2	Herpes Simplex Virus II	2005-2006	laboratory	viral infection	NA
LBXGLT	Two Hour Glucose(OGTT) (mg/dL)	2005-2006	laboratory	biochemistry	NA
PHAFSTHR	Total length of food fast, hours	2005-2006	laboratory	biochemistry	NA
PHAFSTMN	Total length of food fast, minutes	2005-2006	laboratory	biochemistry	NA
LBXBCD	Cadmium (ug/L)	2005-2006	laboratory	heavy metals	NA
LBXBPB	Lead (ug/dL)	2005-2006	laboratory	heavy metals	NA
LBXP1	Total prostate specific antigen (ng/mL)	2005-2006	laboratory	biochemistry	NA
LBXP2	Free prostate specific antigen (ng/mL)	2005-2006	laboratory	biochemistry	NA
LBDP3	Prostate specific antigen ratio (%)	2005-2006	laboratory	biochemistry	NA
LBXPT21	Parathyroid Hormone(Elecys method) pg/mL	2005-2006	laboratory	hormone	NA
LBXTC	Total cholesterol (mg/dL)	2005-2006	laboratory	biochemistry	NA
LBXTFR	Transferrin Receptor (mg/L)	2005-2006	laboratory	biochemistry	NA
LBXTHG	Mercury, total (ug/L)	2005-2006	laboratory	heavy metals	NA
LBXIHG	Mercury, inorganic (ug/L)	2005-2006	laboratory	heavy metals	NA
LBXTR	Triglyceride (mg/dL)	2005-2006	laboratory	biochemistry	NA
LBDLDL	LDL-cholesterol (mg/dL)	2005-2006	laboratory	biochemistry	NA
LBXAPB	Apolipoprotein (B) (mg/dL)	2005-2006	laboratory	biochemistry	NA
URXUHG	Mercury, urine (ng/mL)	2005-2006	laboratory	heavy metals	NA
CFDRIGHT	Exercises Correct	1999-2000	questionnaire	cognitive functioning	NA
URXUBA	Barium, urine (ug/L)	2005-2006	laboratory	heavy metals	NA
URXUBE	Beryllium, urine (ug/L)	2005-2006	laboratory	heavy metals	NA
URXUCD	Cadmium, urine (ug/L)	2005-2006	laboratory	heavy metals	NA
URXUCO	Cobalt, urine (ug/L)	2005-2006	laboratory	heavy metals	NA
URXUCS	Cesium, urine (ug/L)	2005-2006	laboratory	heavy metals	NA
URXUMO	Molybdenum, urine (ug/L)	2005-2006	laboratory	heavy metals	NA
URXUPB	Lead, urine (ug/L)	2005-2006	laboratory	heavy metals	NA
URXUPT	Platinum, urine (ug/L)	2005-2006	laboratory	heavy metals	NA
URXUSB	Antimony, urine (ug/L)	2005-2006	laboratory	heavy metals	NA
URXUTL	Thallium, urine (ug/L)	2005-2006	laboratory	heavy metals	NA
URXUTU	Tungsten, urine (ug/L)	2005-2006	laboratory	heavy metals	NA
URXUUR	Uranium, urinary (ug/L)	2005-2006	laboratory	heavy metals	NA
URXUIO	Iodine, urine (ng/mL)	2005-2006	laboratory	heavy metals	NA
CFDFINSH	Exercises Finished	1999-2000	questionnaire	cognitive functioning	NA
URXCCC	cis dichlorovnl-dimeth carboacid (ug/L)	1999-2000	laboratory	pesticides	NA
LBXHBC	Hepatitis B core antibody	2001-2002	laboratory	viral infection	NA
LBDHBG	Hepatitis B surface antigen	2001-2002	laboratory	viral infection	NA
LBDHCV	Hepatitis C antibody (confirmed)	2001-2002	laboratory	viral infection	NA
LBDHD	Hepatitis D (anti-HDV)	2001-2002	laboratory	viral infection	NA
LBXHBS	Hepatitis B Surface Antibody	2001-2002	laboratory	viral infection	NA
LBXHA	Hepatitis A Antibody (Anti-HAV)	2001-2002	laboratory	viral infection	NA
LBDHI	HIV antibody test result	2001-2002	laboratory	viral infection	NA
LBXCD4	CD4 counts (cells/mm3)	2001-2002	laboratory	viral infection	NA
LBXCD8	CD8 counts (cells/mm3)	2001-2002	laboratory	viral infection	NA
LBXWBF	Water Bromoform (ng/mL)	2001-2002	laboratory	volatile compounds	NA
LBXWCF	Water Chloroform (ng/mL)	2001-2002	laboratory	volatile compounds	NA
LBXWBM	Water Bromodichloromethane (ng/mL)	2001-2002	laboratory	volatile compounds	NA
LBXWCM	Water Dibromochloromethane (ng/mL)	2001-2002	laboratory	volatile compounds	NA
LBXWME	Water MTBE (ng/mL)	2001-2002	laboratory	volatile compounds	NA
LBXV4C	Blood Tetrachloroethene (ng/mL)	2001-2002	laboratory	volatile compounds	NA
LBXVBF	Blood Bromoform (pg/mL)	2001-2002	laboratory	volatile compounds	NA
LBXVBM	Blood Bromodichloromethane (pg/mL)	2001-2002	laboratory	volatile compounds	NA
LBXVBZ	Blood Benzene (ng/mL)	2001-2002	laboratory	volatile compounds	NA
LBXVCF	Blood Chloroform (pg/mL)	2001-2002	laboratory	volatile compounds	NA
LBXVCM	Blood Dibromochloromethane (pg/mL)	2001-2002	laboratory	volatile compounds	NA
LBXVCT	Blood Carbon Tetrachloride (ng/mL)	2001-2002	laboratory	volatile compounds	NA
LBXVDB	Blood 1,4-Dichlorobenzene (ng/mL)	2001-2002	laboratory	volatile compounds	NA
LBXVEB	Blood Ethylbenzene (ng/mL)	2001-2002	laboratory	volatile compounds	NA
LBXVME	Blood MTBE (pg/mL)	2001-2002	laboratory	volatile compounds	NA
LBXVOX	Blood o-Xylene (ng/mL)	2001-2002	laboratory	volatile compounds	NA
LBXVST	Blood Styrene (ng/mL)	2001-2002	laboratory	volatile compounds	NA
LBXVTC	Blood Trichloroethene (ng/mL)	2001-2002	laboratory	volatile compounds	NA
LBXVTO	Blood Toluene (ng/mL)	2001-2002	laboratory	volatile compounds	NA
LBXVXY	Blood m-/p-Xylene (ng/mL)	2001-2002	laboratory	volatile compounds	NA
URXUGC	Gonorrhea, urine	2001-2002	laboratory	bacterial infection	NA
URXUCL	Chlamydia, urine	2001-2002	laboratory	bacterial infection	NA
LBXBCD	Cadmium (ug/L)	2001-2002	laboratory	heavy metals	NA
LBXBPB	Lead (ug/dL)	2001-2002	laboratory	heavy metals	NA
LBXRBF	Folate, RBC (ng/mL RBC)	2001-2002	laboratory	nutrients	NA
LBXTHG	Mercury, total (ug/L)	2001-2002	laboratory	heavy metals	NA
LBXIHG	Mercury, inorganic (ug/L)	2001-2002	laboratory	heavy metals	NA
LBXHCY	Homocysteine (umol/L)	2001-2002	laboratory	biochemistry	NA
LBXFER	Ferritin (ng/mL)	2001-2002	laboratory	biochemistry	NA
LBXB12	Vitamin B12, serum (pg/mL)	2001-2002	laboratory	nutrients	NA
LBXFOL	Folate, serum (ng/mL)	2001-2002	laboratory	nutrients	NA
LBXMMA	Methylmalonic acid (umol/L)	2001-2002	laboratory	biochemistry	NA
LBXCOT	Cotinine (ng/mL)	2001-2002	laboratory	cotinine	NA
URXUHG	Mercury, urine (ng/mL)	2001-2002	laboratory	heavy metals	NA
URXUBA	Barium, urine (ng/mL)	2001-2002	laboratory	heavy metals	NA
URXUBE	Beryllium, urine (ng/mL)	2001-2002	laboratory	heavy metals	NA
URXUCD	Cadmium, urine (ng/mL)	2001-2002	laboratory	heavy metals	NA
URXUCO	Cobalt, urine (ng/mL)	2001-2002	laboratory	heavy metals	NA
URXUCS	Cesium, urine (ng/mL)	2001-2002	laboratory	heavy metals	NA
URXUMO	Molybdenum, urine (ng/mL)	2001-2002	laboratory	heavy metals	NA
URXUPB	Lead, urine (ng/mL)	2001-2002	laboratory	heavy metals	NA
URXUPT	Platinum, urine (ng/mL)	2001-2002	laboratory	heavy metals	NA
URXUSB	Antimony, urine (ng/mL)	2001-2002	laboratory	heavy metals	NA
URXUTL	Thallium, urine (ng/mL)	2001-2002	laboratory	heavy metals	NA
URXUTU	Tungsten, urine (ng/mL)	2001-2002	laboratory	heavy metals	NA
URXUUR	Uranium, urine (ng/mL)	2001-2002	laboratory	heavy metals	NA
URXUIO	Iodine, urine (ng/mL)	2001-2002	laboratory	heavy metals	NA
LBXVID	Vitamin D (ng/mL)	2001-2002	laboratory	nutrients	NA
LBXALC	a-carotene(ug/dL)	2001-2002	laboratory	nutrients	NA
LBXBEC	trans-b-carotene(ug/dL)	2001-2002	laboratory	nutrients	NA
LBXCBC	cis-b-carotene(ug/dL)	2001-2002	laboratory	nutrients	NA
LBXCRY	b-cryptoxanthin(ug/dL)	2001-2002	laboratory	nutrients	NA
LBXGTC	g-tocopherol(ug/dL)	2001-2002	laboratory	nutrients	NA
LBXLUZ	Combined Lutein/zeaxanthin(ug/dL)	2001-2002	laboratory	nutrients	NA
LBXLYC	trans-lycopene(ug/dL)	2001-2002	laboratory	nutrients	NA
LBXRPL	Retinyl palmitate(ug/dL)	2001-2002	laboratory	nutrients	NA
LBXRST	Retinyl stearate(ug/dL)	2001-2002	laboratory	nutrients	NA
LBXVIA	Retinol(ug/dL)	2001-2002	laboratory	nutrients	NA
LBXVIE	a-tocopherol(ug/dL)	2001-2002	laboratory	nutrients	NA
LBXHE1	Herpes I	2001-2002	laboratory	viral infection	NA
LBXHE2	Herpes II	2001-2002	laboratory	viral infection	NA
LBXGH	Glycohemoglobin (%)	2001-2002	laboratory	biochemistry	NA
LBXGLU	Glucose, plasma (mg/dL)	2001-2002	laboratory	biochemistry	NA
LBXGLUSI	Plasma glucose: SI(mmol/L)	2001-2002	laboratory	biochemistry	NA
LBXCPSI	C-peptide: SI(nmol/L)	2001-2002	laboratory	biochemistry	NA
LBXIN	Insulin (uU/mL)	2001-2002	laboratory	hormone	NA
LBXINSI	Insulin: SI(pmol/L)	2001-2002	laboratory	hormone	NA
LBXCRP	C-reactive protein(mg/dL)	2001-2002	laboratory	biochemistry	NA
LBXFB	Fibrinogen (mg/dL)	2001-2002	laboratory	biochemistry	NA
LBXBAP	Bone alkaline phosphotase (ug/L)	2001-2002	laboratory	biochemistry	NA
URXNT	N-Telopeptides (nmol BCE)	2001-2002	laboratory	biochemistry	NA
LBXP1	PSA. total (ng/mL)	2001-2002	laboratory	biochemistry	NA
LBXP2	PSA, free (ng/mL)	2001-2002	laboratory	biochemistry	NA
LBDP3	Prostate specific antigen ratio (%)	2001-2002	laboratory	biochemistry	NA
LBXTC	Total cholesterol (mg/dL)	2001-2002	laboratory	biochemistry	NA
LBDHDL	HDL-cholesterol (mg/dL)	2001-2002	laboratory	biochemistry	NA
LBXTR	Triglyceride (mg/dL)	2001-2002	laboratory	biochemistry	NA
LBDLDL	LDL-cholesterol (mg/dL)	2001-2002	laboratory	biochemistry	NA
URXUCR	Creatinine, urine (mg/dL)	2001-2002	laboratory	biochemistry	NA
URXUCRSI	Creatinine, urine (umol/L)	2001-2002	laboratory	biochemistry	NA
URXUMA	Albumin, urine (ug/mL)	2001-2002	laboratory	biochemistry	NA
URXUMASI	Albumin, urine (mg/L) SI	2001-2002	laboratory	biochemistry	NA
LBXTO1	Toxoplasma (IgG)	2001-2002	laboratory	bacterial infection	NA
LBXTO2	Toxoplasma (IgM)	2001-2002	laboratory	bacterial infection	NA
LBXTO3	Toxoplasma (Dye)	2001-2002	laboratory	bacterial infection	NA
LBXTO5	Toxoplasma (Avidity) IgG	2001-2002	laboratory	bacterial infection	NA
LBXME	Measles	2001-2002	laboratory	bacterial infection	NA
LBDRUIU	Rubella international units	2001-2002	laboratory	bacterial infection	NA
LBXVAR	Varicella	2001-2002	laboratory	bacterial infection	NA
LBXDFS	Floor, GFAAS (ug/sq.ft.)	2001-2002	laboratory	heavy metals	NA
LBXDFSF	Floor, FAAS (ug/sq. ft.)	2001-2002	laboratory	heavy metals	NA
LBDDWS	Window, FAAS (ug/sq. ft.)	2001-2002	laboratory	heavy metals	NA
LBXWBCSI	White blood cell count (SI)	2001-2002	laboratory	blood	NA
LBXLYPCT	Lymphocyte percent (%)	2001-2002	laboratory	blood	NA
LBXMOPCT	Monocyte percent (%)	2001-2002	laboratory	blood	NA
LBXNEPCT	Segmented neutrophils percent (%)	2001-2002	laboratory	blood	NA
LBXEOPCT	Eosinophils percent (%)	2001-2002	laboratory	blood	NA
LBXBAPCT	Basophils percent (%)	2001-2002	laboratory	blood	NA
LBDLYMNO	Lymphocyte number	2001-2002	laboratory	blood	NA
LBDMONO	Monocyte number	2001-2002	laboratory	blood	NA
LBDNENO	Segmented neutrophils number	2001-2002	laboratory	blood	NA
LBDEONO	Eosinophils number	2001-2002	laboratory	blood	NA
LBDBANO	Basophils number	2001-2002	laboratory	blood	NA
LBXRBCSI	Red cell count SI	2001-2002	laboratory	blood	NA
LBXHGB	Hemoglobin (g/dL)	2001-2002	laboratory	blood	NA
LBXHCT	Hematocrit (%)	2001-2002	laboratory	blood	NA
LBXMCVSI	Mean cell volume (fL)	2001-2002	laboratory	blood	NA
LBXMCHSI	Mean cell hemoglobin (pg)	2001-2002	laboratory	blood	NA
LBXMC	MCHC (g/dL)	2001-2002	laboratory	blood	NA
LBXRDW	Red cell distribution width (%)	2001-2002	laboratory	blood	NA
LBXPLTSI	Platelet count (%) SI	2001-2002	laboratory	blood	NA
LBXMPSI	Mean platelet volume (fL)	2001-2002	laboratory	blood	NA
URX14D	2,5-dichlorophenol (ug/L) result	2001-2002	laboratory	pesticides	NA
URX1TB	2,4,5-trichlorophenol (ug/L) result	2001-2002	laboratory	pesticides	NA
URX24D	2,4-D (ug/L) result	2001-2002	laboratory	pesticides	NA
URX25T	2,4,5-T (ug/L) result	2001-2002	laboratory	pesticides	NA
URX3TB	2,4,6-trichlorophenol (ug/L) result	2001-2002	laboratory	pesticides	NA
URXATZ	Atrazine mercapturate (ug/L) result	2001-2002	laboratory	pesticides	NA
URXCBF	Carbofuranphenol (ug/L) result	2001-2002	laboratory	pesticides	NA
URXDEE	DEET (ug/L)	2001-2002	laboratory	pesticides	NA
URXCPM	3,5,6-trichloropyridinol (ug/L) result	2001-2002	laboratory	pesticides	NA
URXDIZ	Oxypyrimidine (ug/L) result	2001-2002	laboratory	pesticides	NA
URXPAR	Paranitrophenol (ug/L) result	2001-2002	laboratory	pesticides	NA
URXPCP	Pentachlorophenol (ug/L) result	2001-2002	laboratory	pesticides	NA
URXPPX	2-isopropoxyphenol (ug/L) result	2001-2002	laboratory	pesticides	NA
URXOPP	O-Phenyl phenol (ug/L) result	2001-2002	laboratory	pesticides	NA
URXOP1	Dimethylphosphate (ug/L) result	2001-2002	laboratory	diakyl	NA
URXOP2	Diethylphosphate (ug/L) result	2001-2002	laboratory	diakyl	NA
URXOP3	Dimethylthiophosphate (ug/L) result	2001-2002	laboratory	diakyl	NA
URXOP4	Diethylthiophosphate (ug/L) result	2001-2002	laboratory	diakyl	NA
URXOP5	Dimethyldithiophosphate (ug/L) result	2001-2002	laboratory	diakyl	NA
URXOP6	Diethyldithiophosphate (ug/L) result	2001-2002	laboratory	diakyl	NA
URX4FP	4-fluoro-3-phenoxybenzoic (ug/L) acid	2001-2002	laboratory	pesticides	NA
URXCB3	dibromovinyl-dimeth prop carboacid(ug/L)	2001-2002	laboratory	pesticides	NA
URXCCC	dichlorovnl-dimeth prop carboacid (ug/L)	2001-2002	laboratory	pesticides	NA
URXCMH	chloro-hydro-meth-chromen-one/ol (ug/L)	2001-2002	laboratory	pesticides	NA
URXDPY	diethylaminomethylpyrimidinol/one (ug/L)	2001-2002	laboratory	pesticides	NA
URXMET	Metolachlor mercapturate (ug/L) result	2001-2002	laboratory	pesticides	NA
URXOPM	3-phenoxybenzoic (ug/L) acid result	2001-2002	laboratory	diakyl	NA
URXTCC	dichlorovnl-dimeth prop carboacid (ug/L)	2001-2002	laboratory	pesticides	NA
URXACE	Acetochlor mercapturate (ug/L) result	2001-2002	laboratory	pesticides	NA
LBX052	PCB52 (ng/g)	2001-2002	laboratory	pcbs	NA
LBX066	PCB66 (ng/g)	2001-2002	laboratory	pcbs	NA
LBX074	PCB74 (ng/g)	2001-2002	laboratory	pcbs	NA
LBX087	PCB87 (ng/g)	2001-2002	laboratory	pcbs	NA
LBX099	PCB99 (ng/g)	2001-2002	laboratory	pcbs	NA
LBX101	PCB101 (ng/g)	2001-2002	laboratory	pcbs	NA
LBX105	PCB105 (ng/g)	2001-2002	laboratory	pcbs	NA
LBX110	PCB110 (ng/g)	2001-2002	laboratory	pcbs	NA
LBX118	PCB118 (ng/g)	2001-2002	laboratory	pcbs	NA
LBX128	PCB128 (ng/g)	2001-2002	laboratory	pcbs	NA
LBX138	PCB138 (ng/g)	2001-2002	laboratory	pcbs	NA
LBX146	PCB146 (ng/g)	2001-2002	laboratory	pcbs	NA
LBX149	PCB149 (ng/g)	2001-2002	laboratory	pcbs	NA
LBX151	PCB151 (ng/g)	2001-2002	laboratory	pcbs	NA
LBX153	PCB153 (ng/g)	2001-2002	laboratory	pcbs	NA
LBX156	PCB156 (ng/g)	2001-2002	laboratory	pcbs	NA
LBX157	PCB157 (ng/g)	2001-2002	laboratory	pcbs	NA
LBX167	PCB167 (ng/g)	2001-2002	laboratory	pcbs	NA
LBX170	PCB170 (ng/g)	2001-2002	laboratory	pcbs	NA
LBX172	PCB172 (ng/g)	2001-2002	laboratory	pcbs	NA
LBX177	PCB177 (ng/g)	2001-2002	laboratory	pcbs	NA
LBX178	PCB178 (ng/g)	2001-2002	laboratory	pcbs	NA
LBX180	PCB180 (ng/g)	2001-2002	laboratory	pcbs	NA
LBX183	PCB183 (ng/g)	2001-2002	laboratory	pcbs	NA
LBX187	PCB187 (ng/g)	2001-2002	laboratory	pcbs	NA
LBX189	PCB189 (ng/g)	2001-2002	laboratory	pcbs	NA
LBX194	PCB194 (ng/g)	2001-2002	laboratory	pcbs	NA
LBX195	PCB195 (ng/g)	2001-2002	laboratory	pcbs	NA
LBX196	PCB196 (ng/g)	2001-2002	laboratory	pcbs	NA
LBD199	PCB199 (ng/g)	2001-2002	laboratory	pcbs	NA
LBX206	PCB206 (ng/g)	2001-2002	laboratory	pcbs	NA
LBXD01	1,2,3,7,8-pncdd (fg/g)	2001-2002	laboratory	dioxins	NA
LBXD02	1,2,3,4,7,8-hxcdd (fg/g)	2001-2002	laboratory	dioxins	NA
LBXD03	1,2,3,6,7,8-hxcdd (fg/g)	2001-2002	laboratory	dioxins	NA
LBXD04	1,2,3,7,8,9-hxcdd (fg/g)	2001-2002	laboratory	dioxins	NA
LBXD05	1,2,3,4,6,7,8-hpcdd (fg/g)	2001-2002	laboratory	dioxins	NA
LBXD07	1,2,3,4,6,7,8,9-ocdd (fg/g)	2001-2002	laboratory	dioxins	NA
LBXF01	2,3,7,8-tcdf (fg/g)	2001-2002	laboratory	furans	NA
LBXF02	1,2,3,7,8-pncdf (fg/g)	2001-2002	laboratory	furans	NA
LBXF03	2,3,4,7,8-pncdf (fg/g)	2001-2002	laboratory	furans	NA
LBXF04	1,2,3,4,7,8-hcxdf (fg/g)	2001-2002	laboratory	furans	NA
LBXF05	1,2,3,6,7,8-hxcdf (fg/g)	2001-2002	laboratory	furans	NA
LBXF06	1,2,3,7,8,9-hxcdf (fg/g)	2001-2002	laboratory	furans	NA
LBXF07	2,3,4,6,7,8-hxcdf (fg/g)	2001-2002	laboratory	furans	NA
LBXF08	1,2,3,4,6,7,8-hpcdf (fg/g)	2001-2002	laboratory	furans	NA
LBXF09	1,2,3,4,7,8,9-hpcdf (fg/g)	2001-2002	laboratory	furans	NA
LBXF10	1,2,3,4,6,7,8,9-ocdf (fg/g)	2001-2002	laboratory	furans	NA
LBXPCB	3,3',4,4',5-pncb (fg/g)	2001-2002	laboratory	pcbs	NA
LBXTC2	3,4,4',5-tcb (fg/g)	2001-2002	laboratory	pcbs	NA
LBXTCD	2,3,7,8-tcdd (fg/g)	2001-2002	laboratory	dioxins	NA
LBXBHC	Beta-hexachlorocyclohexane (ng/g)	2001-2002	laboratory	pesticides	NA
LBXGHC	Gamma-hexachlorocyclohexane (ng/g)	2001-2002	laboratory	pesticides	NA
LBXHCB	Hexachlorobenzene (ng/g)	2001-2002	laboratory	pesticides	NA
LBXHPE	Heptachlor Epoxide (ng/g)	2001-2002	laboratory	pesticides	NA
LBXHXC	3,3',4,4',5,5'-hxcb (fg/g)	2001-2002	laboratory	pcbs	NA
LBXMIR	Mirex (ng/g)	2001-2002	laboratory	pesticides	NA
LBXODT	o,p'-DDT (ng/g)	2001-2002	laboratory	pesticides	NA
LBXOXY	Oxychlordane (ng/g)	2001-2002	laboratory	pesticides	NA
LBXPDE	p,p'-DDE (ng/g)	2001-2002	laboratory	pesticides	NA
LBXPDT	p,p'-DDT (ng/g)	2001-2002	laboratory	pesticides	NA
LBXTNA	Trans-nonachlor (ng/g)	2001-2002	laboratory	pesticides	NA
LBXDIE	Dieldrin (ng/g)	2001-2002	laboratory	pesticides	NA
LBXALD	Aldrin (ng/g)	2001-2002	laboratory	pesticides	NA
LBXEND	Endrin (ng/g)	2001-2002	laboratory	pesticides	NA
LBXTV	Trichomonas Vaginalis	2001-2002	laboratory	bacterial infection	NA
LBXBV	Bacterial Vaginosis	2001-2002	laboratory	bacterial infection	NA
LBXMS1	S. aureus present 1	2001-2002	laboratory	bacterial infection	NA
LBXM1	MRSA 1	2001-2002	laboratory	bacterial infection	NA
LBXMT1	Tetracycline 1	2001-2002	laboratory	bacterial infection	NA
LBXMC1	Clindamycin 1	2001-2002	laboratory	bacterial infection	NA
LBXME1	Erythromycin 1	2001-2002	laboratory	bacterial infection	NA
LBXMP1	Penicillin 1	2001-2002	laboratory	bacterial infection	NA
LBXMI1	Imipenem 1	2001-2002	laboratory	bacterial infection	NA
LBXMF1	Cefazolin 1	2001-2002	laboratory	bacterial infection	NA
LBXMO1	Oxacillin 1	2001-2002	laboratory	bacterial infection	NA
LBXMG1	Gentamicin 1	2001-2002	laboratory	bacterial infection	NA
LBXMD1	Ciprofloxacin 1	2001-2002	laboratory	bacterial infection	NA
LBXML1	Levofloxacin 1	2001-2002	laboratory	bacterial infection	NA
LBXMR1	Rifampin 1	2001-2002	laboratory	bacterial infection	NA
LBXMY1	Amoxicillin/k Clavulanate 1	2001-2002	laboratory	bacterial infection	NA
LBXETA	Enterotoxin A	2001-2002	laboratory	bacterial infection	NA
LBXETB	Enterotoxin B	2001-2002	laboratory	bacterial infection	NA
LBXETC	Enterotoxin C	2001-2002	laboratory	bacterial infection	NA
LBXETD	Enterotoxin D	2001-2002	laboratory	bacterial infection	NA
LBXETH	Enterotoxin H	2001-2002	laboratory	bacterial infection	NA
LBXTSS	Toxic Shock Syndrome Toxin 1	2001-2002	laboratory	bacterial infection	NA
LBXPVL	Panton Valentine Leukocidin	2001-2002	laboratory	bacterial infection	NA
LBXSY1	Syphilis IgG EIA	2001-2002	laboratory	bacterial infection	NA
LBDSY3	Syphilis RPR Titer Level	2001-2002	laboratory	bacterial infection	NA
LBDSY4	Syphilis TP-PA	2001-2002	laboratory	bacterial infection	NA
LBXEPP	Protoporphyrin(ug/dL RBC)	2001-2002	laboratory	biochemistry	NA
LBXSAL	Albumin (g/dL)	2001-2002	laboratory	biochemistry	NA
LBXSATSI	ALT: SI (U/L)	2001-2002	laboratory	biochemistry	NA
LBXSASSI	AST: SI (U/L)	2001-2002	laboratory	biochemistry	NA
LBXSAPSI	Alkaline phosphatase (U/L)	2001-2002	laboratory	biochemistry	NA
LBXSBU	Blood urea nitrogen (mg/dL)	2001-2002	laboratory	biochemistry	NA
LBXSCA	Total Calcium (mg/dL)	2001-2002	laboratory	biochemistry	NA
LBXSCH	Cholesterol (mg/dL)	2001-2002	laboratory	biochemistry	NA
LBXSC3SI	Bicarbonate: SI (mmol/L)	2001-2002	laboratory	biochemistry	NA
LBXSGTSI	GGT: SI (U/L)	2001-2002	laboratory	biochemistry	NA
LBXSGL	Glucose, serum (mg/dL)	2001-2002	laboratory	biochemistry	NA
LBXSIR	Iron (ug/dL)	2001-2002	laboratory	biochemistry	NA
LBXSLDSI	LDH (U/L)	2001-2002	laboratory	hormone	NA
LBXSPH	Phosphorus (mg/dL)	2001-2002	laboratory	biochemistry	NA
LBXSTB	Bilirubin, total (mg/dL)	2001-2002	laboratory	biochemistry	NA
LBXSTP	Total protein (g/dL)	2001-2002	laboratory	biochemistry	NA
LBXSTR	Triglycerides (mg/dL)	2001-2002	laboratory	biochemistry	NA
LBXSUA	Uric acid (mg/dL)	2001-2002	laboratory	biochemistry	NA
LBXSCRINV	1/Creatinine (mg/dL)	2001-2002	laboratory	biochemistry	NA
LBXSNASI	Sodium: SI (mmol/L)	2001-2002	laboratory	biochemistry	NA
LBXSKSI	Potassium: SI (mmol/L)	2001-2002	laboratory	biochemistry	NA
LBXSCLSI	Chloride: SI (mmol/L)	2001-2002	laboratory	biochemistry	NA
LBXSOSSI	Osmolality: SI (mmol/Kg)	2001-2002	laboratory	biochemistry	NA
LBXSGB	Globulin (g/dL)	2001-2002	laboratory	biochemistry	NA
LBXFSH	Follicle stimulating hormone (mIU/mL)	2001-2002	laboratory	hormone	NA
LBXLH	Luteinizing hormone (mIU/mL)	2001-2002	laboratory	hormone	NA
LBXIRN	Iron, frozen (ug/dL)	2001-2002	laboratory	nutrients	NA
LBXTIB	Total iron binding capacity (ug/dL)	2001-2002	laboratory	biochemistry	NA
LBDPCT	Transferrin saturation (%)	2001-2002	laboratory	biochemistry	NA
LBXT4	Thyroxine (T4) (ug/dL)	2001-2002	laboratory	hormone	NA
LBXTSH	Thyroid stim hormone (TSH) (IU/L)	2001-2002	laboratory	hormone	NA
URXMBP	Mono-n-butyl phthalate (ng/mL)	2001-2002	laboratory	phthalates	NA
URXMCP	Mono-cyclohexyl phthalate (ng/mL)	2001-2002	laboratory	phthalates	NA
URXMEP	Mono-ethyl phthalate (ng/mL)	2001-2002	laboratory	phthalates	NA
URXMHP	Mono-(2-ethyl)-hexyl phthalate (ng/mL)	2001-2002	laboratory	phthalates	NA
URXMNP	Mono-isononyl phthalate (ng/mL)	2001-2002	laboratory	phthalates	NA
URXMOP	Mono-n-octyl phthalate (ng/mL)	2001-2002	laboratory	phthalates	NA
URXMZP	Mono-benzyl phthalate (ng/mL)	2001-2002	laboratory	phthalates	NA
URXMNM	Mono-n-methyl phthalate	2001-2002	laboratory	phthalates	NA
URXMC1	Mono-(3-carboxypropyl) phthalate	2001-2002	laboratory	phthalates	NA
URXMHH	Mono-(2-ethyl-5-hydroxyhexyl) phthalate	2001-2002	laboratory	phthalates	NA
URXMOH	Mono-(2-ethyl-5-oxohexyl) phthalate	2001-2002	laboratory	phthalates	NA
URXMIB	Mono-isobutyl pthalate	2001-2002	laboratory	phthalates	NA
URXDAZ	Daidzein (ng/mL)	2001-2002	laboratory	phytoestrogens	NA
URXDMA	o-Desmethylangolensin (O-DMA) (ng/mL)	2001-2002	laboratory	phytoestrogens	NA
URXEQU	Equol (ng/mL)	2001-2002	laboratory	phytoestrogens	NA
URXETD	Enterodiol (ng/mL)	2001-2002	laboratory	phytoestrogens	NA
URXETL	Enterolactone (ng/mL)	2001-2002	laboratory	phytoestrogens	NA
URXGNS	Genistein (ng/mL)	2001-2002	laboratory	phytoestrogens	NA
URXP01	1-napthol	2001-2002	laboratory	hydrocarbons	NA
URXP02	2-napthol	2001-2002	laboratory	hydrocarbons	NA
URXP03	3-fluorene (ng/L)	2001-2002	laboratory	hydrocarbons	NA
URXP04	2-fluorene (ng/L)	2001-2002	laboratory	hydrocarbons	NA
URXP05	3-phenanthrene (ng/L)	2001-2002	laboratory	hydrocarbons	NA
URXP06	1-phenanthrene (ng/L)	2001-2002	laboratory	hydrocarbons	NA
URXP07	2-phenanthrene (ng/L)	2001-2002	laboratory	hydrocarbons	NA
URXP08	1-benzo[c] phenanthrene (ng/L)	2001-2002	laboratory	hydrocarbons	NA
URXP09	3-fluoranthene (ng/L)	2001-2002	laboratory	hydrocarbons	NA
URXP10	1-pyrene (ng/L)	2001-2002	laboratory	hydrocarbons	NA
URXP11	2-benzo[c] phenanthrene (ng/L)	2001-2002	laboratory	hydrocarbons	NA
URXP12	1-benzo[a] anthracene (ng/L)	2001-2002	laboratory	hydrocarbons	NA
URXP13	6-chrysene (ng/L)	2001-2002	laboratory	hydrocarbons	NA
URXP14	3-benzo[c] phenanthrene (ng/L)	2001-2002	laboratory	hydrocarbons	NA
URXP15	3-chrysene (ng/L)	2001-2002	laboratory	hydrocarbons	NA
URXP16	3-benz[a] anthracene (ng/L)	2001-2002	laboratory	hydrocarbons	NA
URXP17	9-fluorene	2001-2002	laboratory	hydrocarbons	NA
URXP18	9-phenanthrene	2001-2002	laboratory	hydrocarbons	NA
URXP19	4-phenanthrene	2001-2002	laboratory	hydrocarbons	NA
URXP20	1-chrysene	2001-2002	laboratory	hydrocarbons	NA
URXP21	2-chrysene	2001-2002	laboratory	hydrocarbons	NA
URXP22	4-chrysene	2001-2002	laboratory	hydrocarbons	NA
URXP24	3-benzo(a) pyrene	2001-2002	laboratory	hydrocarbons	NA
URXCB3	dibromovinyl-dimeth prop carboacid(ug/L)	1999-2000	laboratory	pesticides	NA
URX4FP	4-fluoro-3-phenoxybenzoic acid (ug/L)	1999-2000	laboratory	pesticides	NA
URXOP6	Diethyldithiophosphate (ug/L)	1999-2000	laboratory	diakyl	NA
URXOP5	Dimethyldithiophosphate (ug/L)	1999-2000	laboratory	diakyl	NA
URXOP4	Diethylthiophosphate (ug/L)	1999-2000	laboratory	diakyl	NA
URXOP3	Dimethylthiophosphate (ug/L)	1999-2000	laboratory	diakyl	NA
URXOP2	Diethylphosphate (ug/L)	1999-2000	laboratory	diakyl	NA
URXOP1	Dimethylphosphate (ug/L)	1999-2000	laboratory	diakyl	NA
URXOPP	O-Phenyl phenol (ug/L)	1999-2000	laboratory	pesticides	NA
URXPPX	2-isopropoxyphenol (ug/L)	1999-2000	laboratory	pesticides	NA
URXPCP	Pentachlorophenol (ug/L)	1999-2000	laboratory	pesticides	NA
URXPAR	Paranitrophenol (ug/L)	1999-2000	laboratory	pesticides	NA
URXDIZ	Oxypyrimidine (ug/L)	1999-2000	laboratory	pesticides	NA
URXCPM	3,5,6-trichloropyridinol (ug/L)	1999-2000	laboratory	pesticides	NA
URXMAL	Malathion diacid (ug/L)	1999-2000	laboratory	pesticides	NA
URXDEE	DEET (ug/L)	1999-2000	laboratory	pesticides	NA
URXCBF	Carbofuranphenol (ug/L)	1999-2000	laboratory	pesticides	NA
URXATZ	Atrazine mercapturate (ug/L)	1999-2000	laboratory	pesticides	NA
URX3TB	2,4,6-trichlorophenol (ug/L)	1999-2000	laboratory	pesticides	NA
URX25T	2,4,5-T (ug/L)	1999-2000	laboratory	pesticides	NA
URX24D	2,4-D (ug/L)	1999-2000	laboratory	pesticides	NA
URX1TB	2,4,5-trichlorophenol (ug/L)	1999-2000	laboratory	pesticides	NA
URX14D	2,5-dichlorophenol (ug/L)	1999-2000	laboratory	pesticides	NA
LBXMPSI	Mean platelet volume (fL)	1999-2000	laboratory	blood	NA
LBXPLTSI	Platelet count (%) SI	1999-2000	laboratory	blood	NA
LBXRDW	Red cell distribution width (%)	1999-2000	laboratory	blood	NA
LBXMC	MCHC (g/dL)	1999-2000	laboratory	blood	NA
LBXMCHSI	Mean cell hemoglobin (pg)	1999-2000	laboratory	blood	NA
LBXMCVSI	Mean cell volume (fL)	1999-2000	laboratory	blood	NA
LBXHCT	Hematocrit (%)	1999-2000	laboratory	blood	NA
LBXHGB	Hemoglobin (g/dL)	1999-2000	laboratory	blood	NA
LBXRBCSI	Red cell count SI	1999-2000	laboratory	blood	NA
LBDBANO	Basophils number	1999-2000	laboratory	blood	NA
LBDEONO	Eosinophils number	1999-2000	laboratory	blood	NA
LBDNENO	Segmented neutrophils number	1999-2000	laboratory	blood	NA
LBDMONO	Monocyte number	1999-2000	laboratory	blood	NA
LBDLYMNO	Lymphocyte number	1999-2000	laboratory	blood	NA
LBXBAPCT	Basophils percent (%)	1999-2000	laboratory	blood	NA
LBXEOPCT	Eosinophils percent (%)	1999-2000	laboratory	blood	NA
LBXNEPCT	Segmented neutrophils percent (%)	1999-2000	laboratory	blood	NA
LBXMOPCT	Monocyte percent (%)	1999-2000	laboratory	blood	NA
LBXLYPCT	Lymphocyte percent (%)	1999-2000	laboratory	blood	NA
LBXWBCSI	White blood cell count (SI)	1999-2000	laboratory	blood	NA
HRDHG	Mercury, hair (ppm) MDL Version	1999-2000	laboratory	heavy metals	NA
HRXHG	Mercury, hair (ppm)	1999-2000	laboratory	heavy metals	NA
LBXZMB	MTBE (ug/cubic meter)	1999-2000	laboratory	volatile compounds	NA
LBXZDB	1,4-dichlorobenzene (ug/cubic meter)	1999-2000	laboratory	volatile compounds	NA
LBXZXY	m,p-Xylene (ug/cubic meter)	1999-2000	laboratory	volatile compounds	NA
LBXZOX	o-Xylene (ug/cubic meter)	1999-2000	laboratory	volatile compounds	NA
LBXZTI	Trichloroethene (ug/cubic meter)	1999-2000	laboratory	volatile compounds	NA
LBXZTO	Toluene (ug/cubic meter)	1999-2000	laboratory	volatile compounds	NA
LBXZTE	Tetrachloroethene (ug/cubic meter)	1999-2000	laboratory	volatile compounds	NA
LBXZEB	Ethylbenzene (ug/cubic meter)	1999-2000	laboratory	volatile compounds	NA
LBXZCF	Chloroform (ug/cubic meter)	1999-2000	laboratory	volatile compounds	NA
LBXZBZ	Benzene (ug/cubic meter)	1999-2000	laboratory	volatile compounds	NA
LBDDWS	Window, FAAS (ug/sq. ft.)	1999-2000	laboratory	heavy metals	NA
LBXDFSF	Floor, FAAS (ug/sq. ft.)	1999-2000	laboratory	heavy metals	NA
LBXDFS	Floor, GFAAS (ug/sq.ft.)	1999-2000	laboratory	heavy metals	NA
LBXVAR	Varicella	1999-2000	laboratory	bacterial infection	NA
LBDRUIU	Rubella International Units	1999-2000	laboratory	bacterial infection	NA
LBXME	Measles	1999-2000	laboratory	bacterial infection	NA
LBXTSH	Thyroid stim hormone (TSH) (IU/L)	1999-2000	laboratory	hormone	NA
LBXT4	Thyroxine (T4) (ug/dL)	1999-2000	laboratory	hormone	NA
LBXLH	Luteinizing hormone (mIU/mL)	1999-2000	laboratory	hormone	NA
LBXFSH	Follicle stimulating hormone (mIU/mL)	1999-2000	laboratory	hormone	NA
LBXSGB	Globulin (g/dL)	1999-2000	laboratory	biochemistry	NA
LBXSOSSI	Osmolality (mOsml/L)	1999-2000	laboratory	biochemistry	NA
LBXSCLSI	Chloride (mmol/L)	1999-2000	laboratory	biochemistry	NA
LBXSKSI	Potassium (mmol/L)	1999-2000	laboratory	biochemistry	NA
LBXSNASI	Sodium (mmol/L)	1999-2000	laboratory	biochemistry	NA
LBXSCRINV	1/Creatinine (mg/dL)	1999-2000	laboratory	biochemistry	NA
LBXSUA	Uric acid (mg/dL)	1999-2000	laboratory	biochemistry	NA
LBXSTR	Triglycerides (mg/dL)	1999-2000	laboratory	biochemistry	NA
LBXSTP	Protein, total (g/dL)	1999-2000	laboratory	biochemistry	NA
LBXSTB	Bilirubin, total (mg/dL)	1999-2000	laboratory	biochemistry	NA
LBXSPH	Phosphorus (mg/dL)	1999-2000	laboratory	biochemistry	NA
LBXSLDSI	LDH (U/L)	1999-2000	laboratory	hormone	NA
LBXSIR	Iron (ug/dL)	1999-2000	laboratory	biochemistry	NA
LBXSGL	Glucose (mg/dL)	1999-2000	laboratory	biochemistry	NA
LBXSGTSI	GGT (U/L)	1999-2000	laboratory	biochemistry	NA
LBXSC3SI	Bicarbonate (mmol/L)	1999-2000	laboratory	biochemistry	NA
LBXSCH	Cholesterol, total (mg/dL)	1999-2000	laboratory	biochemistry	NA
LBXSCA	Calcium, total (mg/dL)	1999-2000	laboratory	biochemistry	NA
LBXSBU	Blood urea nitrogen (mg/dL)	1999-2000	laboratory	biochemistry	NA
LBXSAPSI	Alkaline phosphotase (U/L)	1999-2000	laboratory	biochemistry	NA
LBXSASSI	AST (U/L)	1999-2000	laboratory	biochemistry	NA
LBXSATSI	ALT (U/L)	1999-2000	laboratory	biochemistry	NA
LBXSAL	Albumin (g/dL)	1999-2000	laboratory	biochemistry	NA
LBXTO3	Toxoplasma (Dye)	1999-2000	laboratory	bacterial infection	NA
LBXTO2	Toxoplasma (IgM)	1999-2000	laboratory	bacterial infection	NA
LBXTO1	Toxoplasma (IgG)	1999-2000	laboratory	bacterial infection	NA
LBDC2	Cryptosporidium (27kDA)	1999-2000	laboratory	bacterial infection	NA
LBDC1	Cryptosporidium (17kDA)	1999-2000	laboratory	bacterial infection	NA
URXUCRSI	Creatinine, urine (umol/L)	1999-2000	laboratory	biochemistry	NA
URXUCR	Creatinine, urine (mg/dL)	1999-2000	laboratory	biochemistry	NA
URXUMASI	Albumin, urine (mg/L) SI	1999-2000	laboratory	biochemistry	NA
URXUMA	Albumin, urine (ug/mL)	1999-2000	laboratory	biochemistry	NA
LBDLDL	LDL-cholesterol (mg/dL)	1999-2000	laboratory	biochemistry	NA
LBXTR	Triglyceride (mg/dL)	1999-2000	laboratory	biochemistry	NA
LBDHDL	HDL-cholesterol (mg/dL)	1999-2000	laboratory	biochemistry	NA
LBXTC	Total cholesterol (mg/dL)	1999-2000	laboratory	biochemistry	NA
URXNT	N-telopeptides (NTx) (nmol BCE)	1999-2000	laboratory	biochemistry	NA
LBXBAP	Bone alkaline phosphotase (ug/L)	1999-2000	laboratory	biochemistry	NA
LBXFB	Fibrinogen (mg/dL)	1999-2000	laboratory	biochemistry	NA
LBXHP1	Helicobacter pylori (ISR)	1999-2000	laboratory	bacterial infection	NA
LBXCRP	C-reactive protein(mg/dL)	1999-2000	laboratory	biochemistry	NA
LBXINSI	Insulin: SI(pmol/L)	1999-2000	laboratory	hormone	NA
LBXIN	Insulin (uU/mL)	1999-2000	laboratory	hormone	NA
LBXCPSI	C-peptide: SI(nmol/L)	1999-2000	laboratory	biochemistry	NA
LBXGLUSI	Plasma glucose: SI(mmol/L)	1999-2000	laboratory	biochemistry	NA
LBXGLU	Glucose, plasma (mg/dL)	1999-2000	laboratory	biochemistry	NA
LBXGH	Glycohemoglobin (%)	1999-2000	laboratory	biochemistry	NA
LBXHE2	Herpes II	1999-2000	laboratory	viral infection	NA
LBXHE1	Herpes I	1999-2000	laboratory	viral infection	NA
URXUTU	Tungsten, urine (ng/mL)	1999-2000	laboratory	heavy metals	NA
URXUTL	Thallium, urine (ng/mL)	1999-2000	laboratory	heavy metals	NA
URXUSB	Antimony, urine (ng/mL)	1999-2000	laboratory	heavy metals	NA
URXUPT	Platinum, urine (ng/mL)	1999-2000	laboratory	heavy metals	NA
URXUPB	Lead, urine (ng/mL)	1999-2000	laboratory	heavy metals	NA
URXUMO	Molybdenum, urine (ng/mL)	1999-2000	laboratory	heavy metals	NA
URXUCS	Cesium, urine (ng/mL)	1999-2000	laboratory	heavy metals	NA
URXUCO	Cobalt, urine (ng/mL)	1999-2000	laboratory	heavy metals	NA
URXUCD	Cadmium, urine (ng/mL)	1999-2000	laboratory	heavy metals	NA
URXUBE	Beryllium, urine (ng/mL)	1999-2000	laboratory	heavy metals	NA
URXUBA	Barium, urine (ng/mL)	1999-2000	laboratory	heavy metals	NA
URXUHG	Mercury, urine (ng/mL)	1999-2000	laboratory	heavy metals	NA
LBXVIE	Vitamin E (ug/dL)	1999-2000	laboratory	nutrients	NA
LBXVIA	Vitamin A (ug/dL)	1999-2000	laboratory	nutrients	NA
LBXRST	Retinyl stearate (ug/dL)	1999-2000	laboratory	nutrients	NA
LBXRPL	Retinyl palmitate (ug/dL)	1999-2000	laboratory	nutrients	NA
LBXGTC	Gamma tocopherol (ug/dL)	1999-2000	laboratory	nutrients	NA
LBXSEL	Selenium (ng/mL)	1999-2000	laboratory	nutrients	NA
LBXCOT	Cotinine (ng/mL)	1999-2000	laboratory	cotinine	NA
LBXRBF	Folate, RBC (ng/mL RBC)	1999-2000	laboratory	nutrients	NA
LBXIHG	Mercury, Inorganic (ug/L)	1999-2000	laboratory	heavy metals	NA
LBXTHG	Mercury, total (ug/L)	1999-2000	laboratory	heavy metals	NA
LBXMMA	Methylmalonic acid (umol/L)	1999-2000	laboratory	biochemistry	NA
LBXHCY	Homocysteine (umol/L)	1999-2000	laboratory	biochemistry	NA
LBXB12	Vitamin B12, serum (pg/mL)	1999-2000	laboratory	nutrients	NA
LBXFOL	Folate, serum (ng/mL)	1999-2000	laboratory	nutrients	NA
LBXFER	Ferritin (ng/mL)	1999-2000	laboratory	biochemistry	NA
LBDPCT	Transferrin saturation (%)	1999-2000	laboratory	biochemistry	NA
LBXTIB	TIBC (ug/dL)	1999-2000	laboratory	biochemistry	NA
LBXIRN	Iron (ug/dL)	1999-2000	laboratory	nutrients	NA
LBXEPP	Protoporphyrin (ug/dL RBC)	1999-2000	laboratory	biochemistry	NA
LBXBCD	Cadmium (ug/L)	1999-2000	laboratory	heavy metals	NA
LBXBPB	Lead (ug/dL)	1999-2000	laboratory	heavy metals	NA
URXUCL	Chlamydia, urine	1999-2000	laboratory	bacterial infection	NA
URXUGC	Gonorrhea, urine	1999-2000	laboratory	bacterial infection	NA
LBXVXY	Blood m-/p-Xylene (ng/ml)	1999-2000	laboratory	volatile compounds	NA
LBXVTO	Blood Toluene (ng/ml)	1999-2000	laboratory	volatile compounds	NA
LBXV3A	Blood 1,1,1-Trichloroethene (ng/ml)	1999-2000	laboratory	volatile compounds	NA
LBXVTC	Blood Trichloroethene (ng/ml)	1999-2000	laboratory	volatile compounds	NA
LBXVST	Blood Styrene (ng/ml)	1999-2000	laboratory	volatile compounds	NA
LBXVOX	Blood o-Xylene (ng/ml)	1999-2000	laboratory	volatile compounds	NA
LBXVME	Blood MTBE (pg/ml)	1999-2000	laboratory	volatile compounds	NA
LBXVEB	Blood Ethylbenzene (ng/ml)	1999-2000	laboratory	volatile compounds	NA
LBXVDB	Blood 1,4-Dichlorobenzene (ng/ml)	1999-2000	laboratory	volatile compounds	NA
LBXVCT	Blood Carbon Tetrachloride (ng/ml)	1999-2000	laboratory	volatile compounds	NA
LBXVCM	Blood Dibromochloromethane (pg/ml)	1999-2000	laboratory	volatile compounds	NA
LBXVCF	Blood Chloroform (pg/ml)	1999-2000	laboratory	volatile compounds	NA
LBXVBZ	Blood Benzene (ng/ml)	1999-2000	laboratory	volatile compounds	NA
LBXVBM	Blood Bromodichloromethane (pg/ml)	1999-2000	laboratory	volatile compounds	NA
LBXVBF	Blood Bromoform (pg/ml)	1999-2000	laboratory	volatile compounds	NA
LBXV4C	Blood Tetrachloroethene (ng/ml)	1999-2000	laboratory	volatile compounds	NA
LBXWME	Water MTBE (ng/ml)	1999-2000	laboratory	volatile compounds	NA
LBXWCM	Water Dibromochloromethane (ng/ml)	1999-2000	laboratory	volatile compounds	NA
LBXWBM	Water Bromodichloromethane (ng/ml)	1999-2000	laboratory	volatile compounds	NA
LBXWCF	Water Chloroform (ng/ml)	1999-2000	laboratory	volatile compounds	NA
LBXWBF	Water Bromoform (ng/ml)	1999-2000	laboratory	volatile compounds	NA
LBXCD8	CD8 counts (cells/mm3)	1999-2000	laboratory	viral infection	NA
LBXCD4	CD4 counts (cells/mm3)	1999-2000	laboratory	viral infection	NA
LBDHI	HIV antibody test result	1999-2000	laboratory	viral infection	NA
LBXHA	Hepatitis A Antibody (Anti-HAV)	1999-2000	laboratory	viral infection	NA
LBDHD	Hepatitis D (anti-HDV)	1999-2000	laboratory	viral infection	NA
LBDHCV	Hepatitis C antibody	1999-2000	laboratory	viral infection	NA
LBDHBG	Hepatitis B surface antigen	1999-2000	laboratory	viral infection	NA
LBXHBC	Hepatitis B core antibody	1999-2000	laboratory	viral infection	NA
LBXHBS	Hepatitis B Surface Antibody	1999-2000	laboratory	viral infection	NA
URXTCC	trans dichlorovnl-dimeth carboacid(ug/L)	1999-2000	laboratory	pesticides	NA
URXOPM	3-phenoxybenzoic acid (ug/L)	1999-2000	laboratory	diakyl	NA
LBX028	PCB28 (ng/g)	1999-2000	laboratory	pcbs	NA
LBX052	PCB52 (ng/g)	1999-2000	laboratory	pcbs	NA
LBX066	PCB66 (ng/g)	1999-2000	laboratory	pcbs	NA
LBX074	PCB74 (ng/g)	1999-2000	laboratory	pcbs	NA
LBX099	PCB99 (ng/g)	1999-2000	laboratory	pcbs	NA
LBX101	PCB101 (ng/g)	1999-2000	laboratory	pcbs	NA
LBX105	PCB105 (ng/g)	1999-2000	laboratory	pcbs	NA
LBX118	PCB118 (ng/g)	1999-2000	laboratory	pcbs	NA
LBX128	PCB128 (ng/g)	1999-2000	laboratory	pcbs	NA
LBX138	PCB138 (ng/g)	1999-2000	laboratory	pcbs	NA
LBX146	PCB146 (ng/g)	1999-2000	laboratory	pcbs	NA
LBX153	PCB153 (ng/g)	1999-2000	laboratory	pcbs	NA
LBX156	PCB156 (ng/g)	1999-2000	laboratory	pcbs	NA
LBX157	PCB157 (ng/g)	1999-2000	laboratory	pcbs	NA
LBX167	PCB167 (ng/g)	1999-2000	laboratory	pcbs	NA
LBX170	PCB170 (ng/g)	1999-2000	laboratory	pcbs	NA
LBX172	PCB172 (ng/g)	1999-2000	laboratory	pcbs	NA
LBX177	PCB177 (ng/g)	1999-2000	laboratory	pcbs	NA
LBX178	PCB178 (ng/g)	1999-2000	laboratory	pcbs	NA
LBX180	PCB180 (ng/g)	1999-2000	laboratory	pcbs	NA
LBX183	PCB183 (ng/g)	1999-2000	laboratory	pcbs	NA
LBX187	PCB187 (ng/g)	1999-2000	laboratory	pcbs	NA
LBXD01	1,2,3,7,8-pncdd (fg/g)	1999-2000	laboratory	dioxins	NA
LBXD03	1,2,3,6,7,8-hxcdd (fg/g)	1999-2000	laboratory	dioxins	NA
LBXD04	1,2,3,7,8,9-hxcdd (fg/g)	1999-2000	laboratory	dioxins	NA
LBXD05	1,2,3,4,6,7,8-hpcdd (fg/g)	1999-2000	laboratory	dioxins	NA
LBXD07	1,2,3,4,6,7,8,9-ocdd (fg/g)	1999-2000	laboratory	dioxins	NA
LBXF01	2,3,7,8-tcdf (fg/g)	1999-2000	laboratory	furans	NA
LBXF02	1,2,3,7,8-pncdf (fg/g)	1999-2000	laboratory	furans	NA
LBXF03	2,3,4,7,8-pncdf (fg/g)	1999-2000	laboratory	furans	NA
LBXF04	1,2,3,4,7,8-hcxdf (fg/g)	1999-2000	laboratory	furans	NA
LBXF05	1,2,3,6,7,8-hxcdf (fg/g)	1999-2000	laboratory	furans	NA
LBXF06	1,2,3,7,8,9-hxcdf (fg/g)	1999-2000	laboratory	furans	NA
LBXF07	2,3,4,6,7,8-hxcdf (fg/g)	1999-2000	laboratory	furans	NA
LBXF08	1,2,3,4,6,7,8-hpcdf (fg/g)	1999-2000	laboratory	furans	NA
LBXF10	1,2,3,4,6,7,8,9-ocdf (fg/g)	1999-2000	laboratory	furans	NA
LBXPCB	3,3',4,4',5-pncb (fg/g)	1999-2000	laboratory	pcbs	NA
LBXTC2	3,4,4',5-tcb (fg/g)	1999-2000	laboratory	pcbs	NA
LBXTCD	2,3,7,8-tcdd (fg/g)	1999-2000	laboratory	dioxins	NA
LBXBHC	Beta-hexachlorocyclohexane (ng/g)	1999-2000	laboratory	pesticides	NA
LBXGHC	Gamma-hexachlorocyclohexane (ng/g)	1999-2000	laboratory	pesticides	NA
LBXHCB	Hexachlorobenzene (ng/g)	1999-2000	laboratory	pesticides	NA
LBXHPE	Heptachlor Epoxide (ng/g)	1999-2000	laboratory	pesticides	NA
LBXHXC	3,3',4,4',5,5'-hxcb (fg/g)	1999-2000	laboratory	pcbs	NA
LBXMIR	Mirex (ng/g)	1999-2000	laboratory	pesticides	NA
LBXODT	o,p'-DDT (ng/g)	1999-2000	laboratory	pesticides	NA
LBXOXY	Oxychlordane (ng/g)	1999-2000	laboratory	pesticides	NA
LBXPDE	p,p'-DDE (ng/g)	1999-2000	laboratory	pesticides	NA
LBXPDT	p,p'-DDT (ng/g)	1999-2000	laboratory	pesticides	NA
LBXTNA	trans-Nonachlor (ng/g)	1999-2000	laboratory	pesticides	NA
URXMBP	mono-n-butyl phthalate (ng/mL)	1999-2000	laboratory	phthalates	NA
URXMCP	mono-cyclohexyl phthalate (ng/mL)	1999-2000	laboratory	phthalates	NA
URXMEP	mono-ethyl phthalate (ng/mL)	1999-2000	laboratory	phthalates	NA
URXMHP	mono-(2-ethyl)-hexyl phthalate (ng/mL)	1999-2000	laboratory	phthalates	NA
URXMNP	mono-isononyl phthalate (ng/mL)	1999-2000	laboratory	phthalates	NA
URXMOP	mono-n-octyl phthalate (ng/mL)	1999-2000	laboratory	phthalates	NA
URXMZP	mono-benzyl phthalate (ng/mL)	1999-2000	laboratory	phthalates	NA
URXDAZ	Daidzein (ng/mL)	1999-2000	laboratory	phytoestrogens	NA
URXDMA	o-Desmethylangolensin (O-DMA) (ng/mL)	1999-2000	laboratory	phytoestrogens	NA
URXEQU	Equol (ng/mL)	1999-2000	laboratory	phytoestrogens	NA
URXETD	Enterodiol (ng/mL)	1999-2000	laboratory	phytoestrogens	NA
URXETL	Enterolactone (ng/mL)	1999-2000	laboratory	phytoestrogens	NA
URXGNS	Genistein (ng/mL)	1999-2000	laboratory	phytoestrogens	NA
URXP03	3-fluorene (ng/L)	1999-2000	laboratory	hydrocarbons	NA
URXP04	2-fluorene (ng/L)	1999-2000	laboratory	hydrocarbons	NA
URXP05	3-phenanthrene (ng/L)	1999-2000	laboratory	hydrocarbons	NA
URXP06	1-phenanthrene (ng/L)	1999-2000	laboratory	hydrocarbons	NA
URXP07	2-phenanthrene (ng/L)	1999-2000	laboratory	hydrocarbons	NA
URXP08	1-benzo[c] phenanthrene (ng/L)	1999-2000	laboratory	hydrocarbons	NA
URXP09	3-fluoranthene (ng/L)	1999-2000	laboratory	hydrocarbons	NA
URXP10	1-pyrene (ng/L)	1999-2000	laboratory	hydrocarbons	NA
URXP11	2-benzo[c] phenanthrene (ng/L)	1999-2000	laboratory	hydrocarbons	NA
URXP12	1-benzo[a] anthracene (ng/L)	1999-2000	laboratory	hydrocarbons	NA
URXP13	6-chrysene (ng/L)	1999-2000	laboratory	hydrocarbons	NA
URXP14	3-benzo[c] phenanthrene (ng/L)	1999-2000	laboratory	hydrocarbons	NA
URXP15	3-chrysene (ng/L)	1999-2000	laboratory	hydrocarbons	NA
URXP16	3-benz[a] anthracene (ng/L)	1999-2000	laboratory	hydrocarbons	NA
LBXHCY	Homocysteine (umol/L)	2005-2006	laboratory	biochemistry	NA
LBXWIO	Iodide, water (ng/mL)	2005-2006	laboratory	perchlorate	NA
LBXWNO	Nitrate, water (ng/mL)	2005-2006	laboratory	perchlorate	NA
LBXWP8	Perchlorate, water (ng/mL)	2005-2006	laboratory	perchlorate	NA
LBXPFOA	Perfluorooctanoic acid	2005-2006	laboratory	polyflourochemicals	NA
LBXPFOS	Perfluorooctane sulfonic acid	2005-2006	laboratory	polyflourochemicals	NA
LBXPFHS	Perfluorohexane sulfonic acid	2005-2006	laboratory	polyflourochemicals	NA
LBXEPAH	2-(N-ethyl-PFOSA) acetate	2005-2006	laboratory	polyflourochemicals	NA
LBXMPAH	2-(N-methyl-PFOSA) acetate	2005-2006	laboratory	polyflourochemicals	NA
LBXPFDE	Perfluorodecanoic acid	2005-2006	laboratory	polyflourochemicals	NA
LBXPFBS	Perfluorobutane sulfonic acid	2005-2006	laboratory	polyflourochemicals	NA
LBXPFHP	Perfluoroheptanoic acid	2005-2006	laboratory	polyflourochemicals	NA
LBXPFNA	Perfluorononanoic acid	2005-2006	laboratory	polyflourochemicals	NA
LBXPFSA	Perfluorooctane sulfonamide	2005-2006	laboratory	polyflourochemicals	NA
LBXPFUA	Perfluoroundecanoic acid	2005-2006	laboratory	polyflourochemicals	NA
LBXPFDO	Perfluorododecanoic acid	2005-2006	laboratory	polyflourochemicals	NA
URXUAS	Urinary total arsenic (ug/L)	2005-2006	laboratory	heavy metals	NA
URXUAS3	Urinary arsenous acid (ug/L)	2005-2006	laboratory	heavy metals	NA
URXUAS5	Urinary Arsenic acid (ug/L)	2005-2006	laboratory	heavy metals	NA
URXUAB	Urinary Arsenobetaine (ug/L)	2005-2006	laboratory	heavy metals	NA
URXUAC	Urinary Arsenocholine (ug/L)	2005-2006	laboratory	heavy metals	NA
URXUDMA	Urinary Dimethylarsonic acid (ug/L)	2005-2006	laboratory	heavy metals	NA
URXUMMA	Urinary Monomethylacrsonic acid (ug/L)	2005-2006	laboratory	heavy metals	NA
URXUTM	Urinary Trimethylarsine Oxide (ug/L)	2005-2006	laboratory	heavy metals	NA
LBXALC	Alpha-carotene (ug/dL)	2005-2006	laboratory	nutrients	NA
LBXBEC	trans-Beta carotene (ug/dL)	2005-2006	laboratory	nutrients	NA
LBXCBC	cis-Beta carotene (ug/dL)	2005-2006	laboratory	nutrients	NA
LBXCRY	b-Cryptoxanthin (ug/dL)	2005-2006	laboratory	nutrients	NA
LBXGTC	g-Tocopherol (ug/dL)	2005-2006	laboratory	nutrients	NA
LBXLUZ	Lutein and zeaxanthin (ug/dL)	2005-2006	laboratory	nutrients	NA
LBXLYC	trans-Lycopene (ug/dL)	2005-2006	laboratory	nutrients	NA
LBXRPL	Retinyl Palmitate (ug/dL)	2005-2006	laboratory	nutrients	NA
LBXRST	Retinyl Stearate (ug/dL)	2005-2006	laboratory	nutrients	NA
LBXVIA	Vitamin A (ug/dL)	2005-2006	laboratory	nutrients	NA
LBXVIE	Vitamin E (ug/dL)	2005-2006	laboratory	nutrients	NA
LBDTLY	Total (cis- and trans-)Lycopene (ug/dL)	2005-2006	laboratory	nutrients	NA
LBXVIC	Vitamin C (mg/dL)	2005-2006	laboratory	nutrients	NA
LBXWBF	Water Bromoform (ng/mL)	2005-2006	laboratory	volatile compounds	NA
LBXWCF	Water Chloroform (ng/mL)	2005-2006	laboratory	volatile compounds	NA
LBXWBM	Water Bromodichloromethane (ng/mL)	2005-2006	laboratory	volatile compounds	NA
LBXWCM	Water Dibromochloromethane (ng/mL)	2005-2006	laboratory	volatile compounds	NA
LBXWME	Water MTBE (ng/mL)	2005-2006	laboratory	volatile compounds	NA
URX14D	2,5-dichlorophenol (ug/L) result	2005-2006	laboratory	pesticides	NA
URXOPP	O-Phenyl phenol (ug/L) result	2005-2006	laboratory	pesticides	NA
URXDCB	2,4-dichlorophenol (ug/L) result	2005-2006	laboratory	pesticides	NA
URX1TB	2,4,5-trichlorophenol (ug/L) result	2005-2006	laboratory	pesticides	NA
URX3TB	2,4,6-trichlorophenol (ug/L) result	2005-2006	laboratory	pesticides	NA
URXBPH	Urinary Bisphenol A (ng/mL)	2005-2006	laboratory	phenols	NA
URXBP3	Urinary Benzophenone-3 (ng/mL)	2005-2006	laboratory	phenols	NA
URX4TO	Urinary 4-tert-octylphenol (ng/mL)	2005-2006	laboratory	phenols	NA
URXTRS	Urinary Triclosan (ng/mL)	2005-2006	laboratory	pesticides	NA
URXBUP	Butyl paraben (ng/ml)	2005-2006	laboratory	phenols	NA
URXEPB	Ethyl paraben (ng/ml)	2005-2006	laboratory	phenols	NA
URXMPB	Methyl paraben (ng/ml)	2005-2006	laboratory	phenols	NA
URXPPB	Propyl paraben (ng/ml)	2005-2006	laboratory	phenols	NA
SSMEL	Melamine	2003-2004	laboratory	melamine	NA
VARICELL	Varicella antibody	2003-2004	laboratory	viral infection	NA
LBXACR	Acrylamide (pmoL/G Hb)	2003-2004	laboratory	acrylamide	NA
LBXGLY	Glycideamide (pmoL/G Hb)	2003-2004	laboratory	acrylamide	NA
URXDAZ	Daidzein (ng/mL)	2003-2004	laboratory	phytoestrogens	NA
URXDMA	o-Desmethylangolensin (O-DMA) (ng/mL)	2003-2004	laboratory	phytoestrogens	NA
URXEQU	Equol (ng/mL)	2003-2004	laboratory	phytoestrogens	NA
URXETD	Enterodiol (ng/mL)	2003-2004	laboratory	phytoestrogens	NA
URXETL	Enterolactone (ng/mL)	2003-2004	laboratory	phytoestrogens	NA
URXGNS	Genistein (ng/mL)	2003-2004	laboratory	phytoestrogens	NA
URX14D	2,5-dichlorophenol (ug/L) result	2003-2004	laboratory	pesticides	NA
URXOPP	O-Phenyl phenol (ug/L) result	2003-2004	laboratory	pesticides	NA
URXDCB	2,4-dichlorophenol (ug/L) result	2003-2004	laboratory	pesticides	NA
URX1TB	2,4,5-trichlorophenol (ug/L) result	2003-2004	laboratory	pesticides	NA
URX3TB	2,4,6-trichlorophenol (ug/L) result	2003-2004	laboratory	pesticides	NA
URXAAZ	Atrazine (ug/L)	2003-2004	laboratory	pesticides	NA
URXAPE	Acephate (ug/L)	2003-2004	laboratory	pesticides	NA
URXCBF	Carbofuranphenol (ug/L)	2003-2004	laboratory	pesticides	NA
URXDAM	Desethyl atrazine mercapturate (ug/L)	2003-2004	laboratory	pesticides	NA
URXDCZ	Diaminochloroatrazine (ug/L)	2003-2004	laboratory	pesticides	NA
URXDPY	Diethamino-methpyrimidin-ol/one (ug/L)	2003-2004	laboratory	pesticides	NA
URXDTZ	Desethyl atrazine (ug/L)	2003-2004	laboratory	pesticides	NA
URXETU	Ethylenethio urea (ug/L)	2003-2004	laboratory	pesticides	NA
URXMET	Metolachlor mercapturate (ug/L)	2003-2004	laboratory	pesticides	NA
URXMMI	Methamidaphos (ug/L)	2003-2004	laboratory	pesticides	NA
URXMTO	Dimethoate (ug/L)	2003-2004	laboratory	pesticides	NA
URXOMO	O-methoate (ug/L)	2003-2004	laboratory	pesticides	NA
URXPCP	Pentachlorophenol (ug/L)	2003-2004	laboratory	pesticides	NA
URXPPX	2-isopropoxyphenol (ug/L)	2003-2004	laboratory	pesticides	NA
URXPTU	Propylenethio urea (ug/L)	2003-2004	laboratory	pesticides	NA
URXSIS	Desisopropryl atrazine (ug/L)	2003-2004	laboratory	pesticides	NA
URXTCC	Dichlorvinyl dimeth-prop carboacid, ug/L	2003-2004	laboratory	pesticides	NA
LBXS06MK	HPV 06 (Merck competitive Luminex assay)	2003-2004	laboratory	viral infection	NA
LBXS11MK	HPV 11 (Merck competitive Luminex assay)	2003-2004	laboratory	viral infection	NA
LBXS16MK	HPV 16 (Merck competitive Luminex assay)	2003-2004	laboratory	viral infection	NA
LBXS18MK	HPV 18 (Merck competitive Luminex assay)	2003-2004	laboratory	viral infection	NA
home_painted_12mos	painted (12mo)	1999-2000	questionnaire	housing	NA
home_painted_12mos	painted (12mo)	2001-2002	questionnaire	housing	NA
home_painted_12mos	painted (12mo)	2003-2004	questionnaire	housing	NA
house_age	house age	1999-2000	questionnaire	housing	NA
house_age	house age	2001-2002	questionnaire	housing	NA
house_age	house age	2003-2004	questionnaire	housing	NA
house_age	house age	2005-2006	questionnaire	housing	NA
house_type	house type	1999-2000	questionnaire	housing	NA
house_type	house type	2001-2002	questionnaire	housing	NA
house_type	house type	2003-2004	questionnaire	housing	NA
house_type	house type	2005-2006	questionnaire	housing	NA
how_many_years_in_house	years in house	1999-2000	questionnaire	housing	NA
how_many_years_in_house	years in house	2001-2002	questionnaire	housing	NA
how_many_years_in_house	years in house	2003-2004	questionnaire	housing	NA
how_many_years_in_house	years in house	2005-2006	questionnaire	housing	NA
old_paint_scraped	old paint scraping	1999-2000	questionnaire	housing	NA
old_paint_scraped	old paint scraping	2001-2002	questionnaire	housing	NA
old_paint_scraped	old paint scraping	2003-2004	questionnaire	housing	NA
paint_chipping_inside	paint chipping inside	1999-2000	questionnaire	housing	NA
paint_chipping_inside	paint chipping inside	2001-2002	questionnaire	housing	NA
paint_chipping_inside	paint chipping inside	2003-2004	questionnaire	housing	NA
paint_chipping_outside	paint chipping outside	1999-2000	questionnaire	housing	NA
paint_chipping_outside	paint chipping outside	2003-2004	questionnaire	housing	NA
private_water_source	private water source	1999-2000	questionnaire	housing	NA
private_water_source	private water source	2001-2002	questionnaire	housing	NA
private_water_source	private water source	2003-2004	questionnaire	housing	NA
private_water_source	private water source	2005-2006	questionnaire	housing	NA
use_water_treatment	use water treatment	1999-2000	questionnaire	housing	NA
use_water_treatment	use water treatment	2001-2002	questionnaire	housing	NA
use_water_treatment	use water treatment	2003-2004	questionnaire	housing	NA
use_water_treatment	use water treatment	2005-2006	questionnaire	housing	NA
drink_five_per_day	drink 5 in a day	1999-2000	questionnaire	alcohol use	NA
drink_five_per_day	drink 5 in a day	2001-2002	questionnaire	alcohol use	NA
drink_five_per_day	drink 5 in a day	2003-2004	questionnaire	alcohol use	NA
drink_five_per_day	drink 5 in a day	2005-2006	questionnaire	alcohol use	NA
quantity_drink_per_day	drink per day	1999-2000	questionnaire	alcohol use	NA
quantity_drink_per_day	drink per day	2001-2002	questionnaire	alcohol use	NA
quantity_drink_per_day	drink per day	2003-2004	questionnaire	alcohol use	NA
quantity_drink_per_day	drink per day	2005-2006	questionnaire	alcohol use	NA
total_days_5drink_year	days 5 drinks in year	1999-2000	questionnaire	alcohol use	NA
total_days_5drink_year	days 5 drinks in year	2001-2002	questionnaire	alcohol use	NA
total_days_5drink_year	days 5 drinks in year	2003-2004	questionnaire	alcohol use	NA
total_days_5drink_year	days 5 drinks in year	2005-2006	questionnaire	alcohol use	NA
total_days_drink_year	days drink in year	1999-2000	questionnaire	alcohol use	NA
total_days_drink_year	days drink in year	2001-2002	questionnaire	alcohol use	NA
total_days_drink_year	days drink in year	2003-2004	questionnaire	alcohol use	NA
total_days_drink_year	days drink in year	2005-2006	questionnaire	alcohol use	NA
DUQ100	Ever used cocaine or other street drug	1999-2000	questionnaire	street drug	NA
DUQ100	Ever used cocaine or other street drug	2001-2002	questionnaire	street drug	NA
DUQ100	Ever used cocaine or other street drug	2003-2004	questionnaire	street drug	NA
DUQ110	#days used street drugs over past year	1999-2000	questionnaire	street drug	NA
DUQ110	#days used street drugs over past year	2001-2002	questionnaire	street drug	NA
DUQ110	#days used street drugs over past year	2003-2004	questionnaire	street drug	NA
DUQ120	Ever used a needle to take street drugs	1999-2000	questionnaire	street drug	NA
DUQ120	Ever used a needle to take street drugs	2001-2002	questionnaire	street drug	NA
DUQ120	Ever used a needle to take street drugs	2003-2004	questionnaire	street drug	NA
DUQ130	#days used needle for street drugs/year	1999-2000	questionnaire	street drug	NA
DUQ130	#days used needle for street drugs/year	2001-2002	questionnaire	street drug	NA
DUQ130	#days used needle for street drugs/year	2003-2004	questionnaire	street drug	NA
DUQ200	Ever used marijuana or hashish	2005-2006	questionnaire	street drug	NA
DUQ210	Age when first tried marijuana	2005-2006	questionnaire	street drug	NA
DUQ230	# days used marijuana or hashish/month	2005-2006	questionnaire	street drug	NA
DUQ240	Ever used cocaine/heroin/methamphetamine	2005-2006	questionnaire	street drug	NA
DUQ250	Ever use any form of cocaine	2005-2006	questionnaire	street drug	NA
DUQ260	Age first used cocaine	2005-2006	questionnaire	street drug	NA
DUQ272	# of time you used cocaine	2005-2006	questionnaire	street drug	NA
DUQ280	# of days used cocaine/month	2005-2006	questionnaire	street drug	NA
DUQ290	Ever used heroin	2005-2006	questionnaire	street drug	NA
DUQ300	Age first used heroin	2005-2006	questionnaire	street drug	NA
DUQ320	# of days used heroin/month	2005-2006	questionnaire	street drug	NA
DUQ330	Ever used methamphetamine	2005-2006	questionnaire	street drug	NA
DUQ340	Age first used methamphetamine	2005-2006	questionnaire	street drug	NA
DUQ352	# times used methamphetamine	2005-2006	questionnaire	street drug	NA
DUQ360	# days used methamphetamine/month	2005-2006	questionnaire	street drug	NA
DUQ370	Ever use a needle to inject illegal drug	2005-2006	questionnaire	street drug	NA
last_time_used_cocaine	last time (days) used cocaine	2005-2006	questionnaire	street drug	NA
last_time_used_heroin	last time (days) used heroin	2005-2006	questionnaire	street drug	NA
last_time_used_marijuana	last time (days) used marijuana	2005-2006	questionnaire	street drug	NA
last_time_used_meth	last time (days) used meth	2005-2006	questionnaire	street drug	NA
ALBUTEROL	ALBUTEROL	1999-2000	questionnaire	pharmaceutical	NA
LEVOTHYROXINE_SODIUM	LEVOTHYROXINE_SODIUM	1999-2000	questionnaire	pharmaceutical	NA
AMOXICILLIN	AMOXICILLIN	1999-2000	questionnaire	pharmaceutical	NA
METFORMIN_HYDROCHLORIDE	METFORMIN_HYDROCHLORIDE	1999-2000	questionnaire	pharmaceutical	NA
FUROSEMIDE	FUROSEMIDE	1999-2000	questionnaire	pharmaceutical	NA
ATORVASTATIN_CALCIUM	ATORVASTATIN_CALCIUM	1999-2000	questionnaire	pharmaceutical	NA
AMLODIPINE_BESYLATE	AMLODIPINE_BESYLATE	1999-2000	questionnaire	pharmaceutical	NA
LISINOPRIL	LISINOPRIL	1999-2000	questionnaire	pharmaceutical	NA
ESTROGENS__CONJUGATED	ESTROGENS__CONJUGATED	1999-2000	questionnaire	pharmaceutical	NA
LORATADINE	LORATADINE	1999-2000	questionnaire	pharmaceutical	NA
ATENOLOL	ATENOLOL	1999-2000	questionnaire	pharmaceutical	NA
OMEPRAZOLE	OMEPRAZOLE	1999-2000	questionnaire	pharmaceutical	NA
IBUPROFEN	IBUPROFEN	1999-2000	questionnaire	pharmaceutical	NA
POTASSIUM_CHLORIDE	POTASSIUM_CHLORIDE	1999-2000	questionnaire	pharmaceutical	NA
HYDROCHLOROTHIAZIDE	HYDROCHLOROTHIAZIDE	1999-2000	questionnaire	pharmaceutical	NA
SIMVASTATIN	SIMVASTATIN	1999-2000	questionnaire	pharmaceutical	NA
INSULIN	INSULIN	1999-2000	questionnaire	pharmaceutical	NA
DIGOXIN	DIGOXIN	1999-2000	questionnaire	pharmaceutical	NA
GLYBURIDE	GLYBURIDE	1999-2000	questionnaire	pharmaceutical	NA
GLIPIZIDE	GLIPIZIDE	1999-2000	questionnaire	pharmaceutical	NA
NIFEDIPINE	NIFEDIPINE	1999-2000	questionnaire	pharmaceutical	NA
DILTIAZEM_HYDROCHLORIDE	DILTIAZEM_HYDROCHLORIDE	1999-2000	questionnaire	pharmaceutical	NA
HYDROCHLOROTHIAZIDE__TRIAMTERENE	HYDROCHLOROTHIAZIDE__TRIAMTERENE	1999-2000	questionnaire	pharmaceutical	NA
FLUTICASONE_PROPIONATE	FLUTICASONE_PROPIONATE	1999-2000	questionnaire	pharmaceutical	NA
VERAPAMIL_HYDROCHLORIDE	VERAPAMIL_HYDROCHLORIDE	1999-2000	questionnaire	pharmaceutical	NA
WARFARIN_SODIUM	WARFARIN_SODIUM	1999-2000	questionnaire	pharmaceutical	NA
LANSOPRAZOLE	LANSOPRAZOLE	1999-2000	questionnaire	pharmaceutical	NA
CELECOXIB	CELECOXIB	1999-2000	questionnaire	pharmaceutical	NA
RANITIDINE_HYDROCHLORIDE	RANITIDINE_HYDROCHLORIDE	1999-2000	questionnaire	pharmaceutical	NA
PREDNISONE	PREDNISONE	1999-2000	questionnaire	pharmaceutical	NA
SERTRALINE_HYDROCHLORIDE	SERTRALINE_HYDROCHLORIDE	1999-2000	questionnaire	pharmaceutical	NA
NAPROXEN	NAPROXEN	1999-2000	questionnaire	pharmaceutical	NA
DOXAZOSIN_MESYLATE	DOXAZOSIN_MESYLATE	1999-2000	questionnaire	pharmaceutical	NA
CETIRIZINE_HYDROCHLORIDE	CETIRIZINE_HYDROCHLORIDE	1999-2000	questionnaire	pharmaceutical	NA
METOPROLOL_TARTRATE	METOPROLOL_TARTRATE	1999-2000	questionnaire	pharmaceutical	NA
QUINAPRIL_HYDROCHLORIDE	QUINAPRIL_HYDROCHLORIDE	1999-2000	questionnaire	pharmaceutical	NA
PRAVASTATIN_SODIUM	PRAVASTATIN_SODIUM	1999-2000	questionnaire	pharmaceutical	NA
ENALAPRIL_MALEATE	ENALAPRIL_MALEATE	1999-2000	questionnaire	pharmaceutical	NA
NITROGLYCERIN	NITROGLYCERIN	1999-2000	questionnaire	pharmaceutical	NA
METHYLPHENIDATE_HYDROCHLORIDE	METHYLPHENIDATE_HYDROCHLORIDE	1999-2000	questionnaire	pharmaceutical	NA
PAROXETINE_HYDROCHLORIDE	PAROXETINE_HYDROCHLORIDE	1999-2000	questionnaire	pharmaceutical	NA
METOPROLOL_SUCCINATE	METOPROLOL_SUCCINATE	1999-2000	questionnaire	pharmaceutical	NA
ACETAMINOPHEN__PROPOXYPHENE_NAPSYLATE	ACETAMINOPHEN__PROPOXYPHENE_NAPSYLATE	1999-2000	questionnaire	pharmaceutical	NA
ESTROGENS__CONJUGATED__MEDROXYPROGESTERONE_ACETATE	ESTROGENS__CONJUGATED__MEDROXYPROGESTERONE_ACETATE	1999-2000	questionnaire	pharmaceutical	NA
MEDROXYPROGESTERONE_ACETATE	MEDROXYPROGESTERONE_ACETATE	1999-2000	questionnaire	pharmaceutical	NA
ACETAMINOPHEN__HYDROCODONE_BITARTRATE	ACETAMINOPHEN__HYDROCODONE_BITARTRATE	1999-2000	questionnaire	pharmaceutical	NA
BECLOMETHASONE_DIPROPIONATE	BECLOMETHASONE_DIPROPIONATE	1999-2000	questionnaire	pharmaceutical	NA
TERAZOSIN_HYDROCHLORIDE	TERAZOSIN_HYDROCHLORIDE	1999-2000	questionnaire	pharmaceutical	NA
AMOXICILLIN_TRIHYDRATE__CLAVULANATE_POTASSIUM	AMOXICILLIN_TRIHYDRATE__CLAVULANATE_POTASSIUM	1999-2000	questionnaire	pharmaceutical	NA
FEXOFENADINE_HYDROCHLORIDE	FEXOFENADINE_HYDROCHLORIDE	1999-2000	questionnaire	pharmaceutical	NA
THEOPHYLLINE	THEOPHYLLINE	1999-2000	questionnaire	pharmaceutical	NA
BUPROPION_HYDROCHLORIDE	BUPROPION_HYDROCHLORIDE	1999-2000	questionnaire	pharmaceutical	NA
CEPHALEXIN	CEPHALEXIN	1999-2000	questionnaire	pharmaceutical	NA
PROPRANOLOL_HYDROCHLORIDE	PROPRANOLOL_HYDROCHLORIDE	1999-2000	questionnaire	pharmaceutical	NA
CLONIDINE	CLONIDINE	1999-2000	questionnaire	pharmaceutical	NA
ACETAMINOPHEN__CODEINE_PHOSPHATE	ACETAMINOPHEN__CODEINE_PHOSPHATE	1999-2000	questionnaire	pharmaceutical	NA
ASPIRIN	ASPIRIN	1999-2000	questionnaire	pharmaceutical	NA
BENAZEPRIL_HYDROCHLORIDE	BENAZEPRIL_HYDROCHLORIDE	1999-2000	questionnaire	pharmaceutical	NA
SULFAMETHOXAZOLE__TRIMETHOPRIM	SULFAMETHOXAZOLE__TRIMETHOPRIM	1999-2000	questionnaire	pharmaceutical	NA
ROFECOXIB	ROFECOXIB	1999-2000	questionnaire	pharmaceutical	NA
ALPRAZOLAM	ALPRAZOLAM	1999-2000	questionnaire	pharmaceutical	NA
FOSINOPRIL_SODIUM	FOSINOPRIL_SODIUM	1999-2000	questionnaire	pharmaceutical	NA
MECLIZINE_HYDROCHLORIDE	MECLIZINE_HYDROCHLORIDE	1999-2000	questionnaire	pharmaceutical	NA
TRIAMCINOLONE_ACETONIDE	TRIAMCINOLONE_ACETONIDE	1999-2000	questionnaire	pharmaceutical	NA
ESTRADIOL	ESTRADIOL	1999-2000	questionnaire	pharmaceutical	NA
TRAZODONE_HYDROCHLORIDE	TRAZODONE_HYDROCHLORIDE	1999-2000	questionnaire	pharmaceutical	NA
CAPTOPRIL	CAPTOPRIL	1999-2000	questionnaire	pharmaceutical	NA
AMITRIPTYLINE_HYDROCHLORIDE	AMITRIPTYLINE_HYDROCHLORIDE	1999-2000	questionnaire	pharmaceutical	NA
ALENDRONATE_SODIUM	ALENDRONATE_SODIUM	1999-2000	questionnaire	pharmaceutical	NA
HYDROXYZINE_HYDROCHLORIDE	HYDROXYZINE_HYDROCHLORIDE	1999-2000	questionnaire	pharmaceutical	NA
SALMETEROL_XINAFOATE	SALMETEROL_XINAFOATE	1999-2000	questionnaire	pharmaceutical	NA
AZITHROMYCIN_DIHYDRATE	AZITHROMYCIN_DIHYDRATE	1999-2000	questionnaire	pharmaceutical	NA
LOSARTAN_POTASSIUM	LOSARTAN_POTASSIUM	1999-2000	questionnaire	pharmaceutical	NA
TRIAMTERENE	TRIAMTERENE	1999-2000	questionnaire	pharmaceutical	NA
ALLOPURINOL	ALLOPURINOL	1999-2000	questionnaire	pharmaceutical	NA
IPRATROPIUM_BROMIDE	IPRATROPIUM_BROMIDE	1999-2000	questionnaire	pharmaceutical	NA
ISOSORBIDE_MONONITRATE	ISOSORBIDE_MONONITRATE	1999-2000	questionnaire	pharmaceutical	NA
HYDROCHLOROTHIAZIDE__LISINOPRIL	HYDROCHLOROTHIAZIDE__LISINOPRIL	1999-2000	questionnaire	pharmaceutical	NA
LORAZEPAM	LORAZEPAM	1999-2000	questionnaire	pharmaceutical	NA
GLIMEPIRIDE	GLIMEPIRIDE	1999-2000	questionnaire	pharmaceutical	NA
ISOSORBIDE_UNSPECIFIED	ISOSORBIDE_UNSPECIFIED	1999-2000	questionnaire	pharmaceutical	NA
ANTIBIOTIC_UNSPECIFIED	ANTIBIOTIC_UNSPECIFIED	1999-2000	questionnaire	pharmaceutical	NA
CLARITHROMYCIN	CLARITHROMYCIN	1999-2000	questionnaire	pharmaceutical	NA
CIMETIDINE_HYDROCHLORIDE	CIMETIDINE_HYDROCHLORIDE	1999-2000	questionnaire	pharmaceutical	NA
DIVALPROEX_SODIUM	DIVALPROEX_SODIUM	1999-2000	questionnaire	pharmaceutical	NA
VALSARTAN	VALSARTAN	1999-2000	questionnaire	pharmaceutical	NA
FLUVASTATIN_SODIUM	FLUVASTATIN_SODIUM	1999-2000	questionnaire	pharmaceutical	NA
MONTELUKAST_SODIUM	MONTELUKAST_SODIUM	1999-2000	questionnaire	pharmaceutical	NA
CROMOLYN_SODIUM	CROMOLYN_SODIUM	1999-2000	questionnaire	pharmaceutical	NA
PHENYTOIN_SODIUM	PHENYTOIN_SODIUM	1999-2000	questionnaire	pharmaceutical	NA
ETHINYL_ESTRADIOL__LEVONORGESTREL	ETHINYL_ESTRADIOL__LEVONORGESTREL	1999-2000	questionnaire	pharmaceutical	NA
LORATADINE__PSEUDOEPHEDRINE_SULFATE	LORATADINE__PSEUDOEPHEDRINE_SULFATE	1999-2000	questionnaire	pharmaceutical	NA
CYCLOBENZAPRINE_HYDROCHLORIDE	CYCLOBENZAPRINE_HYDROCHLORIDE	1999-2000	questionnaire	pharmaceutical	NA
ETHINYL_ESTRADIOL__NORGESTIMATE	ETHINYL_ESTRADIOL__NORGESTIMATE	1999-2000	questionnaire	pharmaceutical	NA
LATANOPROST	LATANOPROST	1999-2000	questionnaire	pharmaceutical	NA
SPIRONOLACTONE	SPIRONOLACTONE	1999-2000	questionnaire	pharmaceutical	NA
HYDROCODONE_UNSPECIFIED	HYDROCODONE_UNSPECIFIED	1999-2000	questionnaire	pharmaceutical	NA
GEMFIBROZIL	GEMFIBROZIL	1999-2000	questionnaire	pharmaceutical	NA
DIAZEPAM	DIAZEPAM	1999-2000	questionnaire	pharmaceutical	NA
ALBUTEROL	ALBUTEROL	2001-2002	questionnaire	pharmaceutical	NA
LEVOTHYROXINE_SODIUM	LEVOTHYROXINE_SODIUM	2001-2002	questionnaire	pharmaceutical	NA
ATORVASTATIN_CALCIUM	ATORVASTATIN_CALCIUM	2001-2002	questionnaire	pharmaceutical	NA
AMOXICILLIN	AMOXICILLIN	2001-2002	questionnaire	pharmaceutical	NA
LISINOPRIL	LISINOPRIL	2001-2002	questionnaire	pharmaceutical	NA
ESTROGENS__CONJUGATED	ESTROGENS__CONJUGATED	2001-2002	questionnaire	pharmaceutical	NA
FUROSEMIDE	FUROSEMIDE	2001-2002	questionnaire	pharmaceutical	NA
ATENOLOL	ATENOLOL	2001-2002	questionnaire	pharmaceutical	NA
SIMVASTATIN	SIMVASTATIN	2001-2002	questionnaire	pharmaceutical	NA
METFORMIN_HYDROCHLORIDE	METFORMIN_HYDROCHLORIDE	2001-2002	questionnaire	pharmaceutical	NA
HYDROCHLOROTHIAZIDE	HYDROCHLOROTHIAZIDE	2001-2002	questionnaire	pharmaceutical	NA
LORATADINE	LORATADINE	2001-2002	questionnaire	pharmaceutical	NA
POTASSIUM_CHLORIDE	POTASSIUM_CHLORIDE	2001-2002	questionnaire	pharmaceutical	NA
FLUTICASONE_PROPIONATE	FLUTICASONE_PROPIONATE	2001-2002	questionnaire	pharmaceutical	NA
INSULIN	INSULIN	2001-2002	questionnaire	pharmaceutical	NA
OMEPRAZOLE	OMEPRAZOLE	2001-2002	questionnaire	pharmaceutical	NA
FEXOFENADINE_HYDROCHLORIDE	FEXOFENADINE_HYDROCHLORIDE	2001-2002	questionnaire	pharmaceutical	NA
WARFARIN_SODIUM	WARFARIN_SODIUM	2001-2002	questionnaire	pharmaceutical	NA
AMLODIPINE_BESYLATE	AMLODIPINE_BESYLATE	2001-2002	questionnaire	pharmaceutical	NA
ROFECOXIB	ROFECOXIB	2001-2002	questionnaire	pharmaceutical	NA
CELECOXIB	CELECOXIB	2001-2002	questionnaire	pharmaceutical	NA
DILTIAZEM_HYDROCHLORIDE	DILTIAZEM_HYDROCHLORIDE	2001-2002	questionnaire	pharmaceutical	NA
CETIRIZINE_HYDROCHLORIDE	CETIRIZINE_HYDROCHLORIDE	2001-2002	questionnaire	pharmaceutical	NA
LANSOPRAZOLE	LANSOPRAZOLE	2001-2002	questionnaire	pharmaceutical	NA
DIGOXIN	DIGOXIN	2001-2002	questionnaire	pharmaceutical	NA
ACETAMINOPHEN__HYDROCODONE_BITARTRATE	ACETAMINOPHEN__HYDROCODONE_BITARTRATE	2001-2002	questionnaire	pharmaceutical	NA
SERTRALINE_HYDROCHLORIDE	SERTRALINE_HYDROCHLORIDE	2001-2002	questionnaire	pharmaceutical	NA
PAROXETINE_HYDROCHLORIDE	PAROXETINE_HYDROCHLORIDE	2001-2002	questionnaire	pharmaceutical	NA
HYDROCHLOROTHIAZIDE__TRIAMTERENE	HYDROCHLOROTHIAZIDE__TRIAMTERENE	2001-2002	questionnaire	pharmaceutical	NA
IBUPROFEN	IBUPROFEN	2001-2002	questionnaire	pharmaceutical	NA
GLYBURIDE	GLYBURIDE	2001-2002	questionnaire	pharmaceutical	NA
RANITIDINE_HYDROCHLORIDE	RANITIDINE_HYDROCHLORIDE	2001-2002	questionnaire	pharmaceutical	NA
GLIPIZIDE	GLIPIZIDE	2001-2002	questionnaire	pharmaceutical	NA
METOPROLOL_SUCCINATE	METOPROLOL_SUCCINATE	2001-2002	questionnaire	pharmaceutical	NA
VERAPAMIL_HYDROCHLORIDE	VERAPAMIL_HYDROCHLORIDE	2001-2002	questionnaire	pharmaceutical	NA
ALENDRONATE_SODIUM	ALENDRONATE_SODIUM	2001-2002	questionnaire	pharmaceutical	NA
NAPROXEN	NAPROXEN	2001-2002	questionnaire	pharmaceutical	NA
METHYLPHENIDATE_HYDROCHLORIDE	METHYLPHENIDATE_HYDROCHLORIDE	2001-2002	questionnaire	pharmaceutical	NA
PREDNISONE	PREDNISONE	2001-2002	questionnaire	pharmaceutical	NA
NIFEDIPINE	NIFEDIPINE	2001-2002	questionnaire	pharmaceutical	NA
MONTELUKAST_SODIUM	MONTELUKAST_SODIUM	2001-2002	questionnaire	pharmaceutical	NA
ACETAMINOPHEN__PROPOXYPHENE_NAPSYLATE	ACETAMINOPHEN__PROPOXYPHENE_NAPSYLATE	2001-2002	questionnaire	pharmaceutical	NA
NITROGLYCERIN	NITROGLYCERIN	2001-2002	questionnaire	pharmaceutical	NA
AMITRIPTYLINE_HYDROCHLORIDE	AMITRIPTYLINE_HYDROCHLORIDE	2001-2002	questionnaire	pharmaceutical	NA
PRAVASTATIN_SODIUM	PRAVASTATIN_SODIUM	2001-2002	questionnaire	pharmaceutical	NA
ETHINYL_ESTRADIOL__NORGESTIMATE	ETHINYL_ESTRADIOL__NORGESTIMATE	2001-2002	questionnaire	pharmaceutical	NA
ESTROGENS__CONJUGATED__MEDROXYPROGESTERONE_ACETATE	ESTROGENS__CONJUGATED__MEDROXYPROGESTERONE_ACETATE	2001-2002	questionnaire	pharmaceutical	NA
CITALOPRAM_HYDROBROMIDE	CITALOPRAM_HYDROBROMIDE	2001-2002	questionnaire	pharmaceutical	NA
AMOXICILLIN_TRIHYDRATE__CLAVULANATE_POTASSIUM	AMOXICILLIN_TRIHYDRATE__CLAVULANATE_POTASSIUM	2001-2002	questionnaire	pharmaceutical	NA
ALLOPURINOL	ALLOPURINOL	2001-2002	questionnaire	pharmaceutical	NA
CEPHALEXIN	CEPHALEXIN	2001-2002	questionnaire	pharmaceutical	NA
TERAZOSIN_HYDROCHLORIDE	TERAZOSIN_HYDROCHLORIDE	2001-2002	questionnaire	pharmaceutical	NA
VALSARTAN	VALSARTAN	2001-2002	questionnaire	pharmaceutical	NA
SALMETEROL_XINAFOATE	SALMETEROL_XINAFOATE	2001-2002	questionnaire	pharmaceutical	NA
CLOPIDOGREL_BISULFATE	CLOPIDOGREL_BISULFATE	2001-2002	questionnaire	pharmaceutical	NA
GABAPENTIN	GABAPENTIN	2001-2002	questionnaire	pharmaceutical	NA
BUDESONIDE	BUDESONIDE	2001-2002	questionnaire	pharmaceutical	NA
TRIAMCINOLONE_ACETONIDE	TRIAMCINOLONE_ACETONIDE	2001-2002	questionnaire	pharmaceutical	NA
ENALAPRIL_MALEATE	ENALAPRIL_MALEATE	2001-2002	questionnaire	pharmaceutical	NA
TRAZODONE_HYDROCHLORIDE	TRAZODONE_HYDROCHLORIDE	2001-2002	questionnaire	pharmaceutical	NA
AZITHROMYCIN_DIHYDRATE	AZITHROMYCIN_DIHYDRATE	2001-2002	questionnaire	pharmaceutical	NA
ESTRADIOL	ESTRADIOL	2001-2002	questionnaire	pharmaceutical	NA
ACETAMINOPHEN__CODEINE_PHOSPHATE	ACETAMINOPHEN__CODEINE_PHOSPHATE	2001-2002	questionnaire	pharmaceutical	NA
LORAZEPAM	LORAZEPAM	2001-2002	questionnaire	pharmaceutical	NA
MEDROXYPROGESTERONE_ACETATE	MEDROXYPROGESTERONE_ACETATE	2001-2002	questionnaire	pharmaceutical	NA
ALPRAZOLAM	ALPRAZOLAM	2001-2002	questionnaire	pharmaceutical	NA
LOSARTAN_POTASSIUM	LOSARTAN_POTASSIUM	2001-2002	questionnaire	pharmaceutical	NA
METOPROLOL_UNSPECIFIED	METOPROLOL_UNSPECIFIED	2001-2002	questionnaire	pharmaceutical	NA
PANTOPRAZOLE_SODIUM	PANTOPRAZOLE_SODIUM	2001-2002	questionnaire	pharmaceutical	NA
BENAZEPRIL_HYDROCHLORIDE	BENAZEPRIL_HYDROCHLORIDE	2001-2002	questionnaire	pharmaceutical	NA
RABEPRAZOLE_SODIUM	RABEPRAZOLE_SODIUM	2001-2002	questionnaire	pharmaceutical	NA
ROSIGLITAZONE_MALEATE	ROSIGLITAZONE_MALEATE	2001-2002	questionnaire	pharmaceutical	NA
QUINAPRIL_HYDROCHLORIDE	QUINAPRIL_HYDROCHLORIDE	2001-2002	questionnaire	pharmaceutical	NA
ISOSORBIDE_MONONITRATE	ISOSORBIDE_MONONITRATE	2001-2002	questionnaire	pharmaceutical	NA
METOPROLOL_TARTRATE	METOPROLOL_TARTRATE	2001-2002	questionnaire	pharmaceutical	NA
AMPHETAMINE_ASPARTATE	AMPHETAMINE_ASPARTATE__AMPHETAMINE_SULFATE__DEXTROAMPHETAMINE_SACCHARATE__DEXTROAMPHETAMINE_SULFATE	2001-2002	questionnaire	pharmaceutical	NA
BUPROPION_HYDROCHLORIDE	BUPROPION_HYDROCHLORIDE	2001-2002	questionnaire	pharmaceutical	NA
FELODIPINE	FELODIPINE	2001-2002	questionnaire	pharmaceutical	NA
MOMETASONE_FUROATE_MONOHYDRATE	MOMETASONE_FUROATE_MONOHYDRATE	2001-2002	questionnaire	pharmaceutical	NA
GLIMEPIRIDE	GLIMEPIRIDE	2001-2002	questionnaire	pharmaceutical	NA
ZOLPIDEM_TARTRATE	ZOLPIDEM_TARTRATE	2001-2002	questionnaire	pharmaceutical	NA
LATANOPROST	LATANOPROST	2001-2002	questionnaire	pharmaceutical	NA
CLONIDINE	CLONIDINE	2001-2002	questionnaire	pharmaceutical	NA
RAMIPRIL	RAMIPRIL	2001-2002	questionnaire	pharmaceutical	NA
ACETAMINOPHEN__OXYCODONE_HYDROCHLORIDE	ACETAMINOPHEN__OXYCODONE_HYDROCHLORIDE	2001-2002	questionnaire	pharmaceutical	NA
SULFAMETHOXAZOLE__TRIMETHOPRIM	SULFAMETHOXAZOLE__TRIMETHOPRIM	2001-2002	questionnaire	pharmaceutical	NA
DOXAZOSIN_MESYLATE	DOXAZOSIN_MESYLATE	2001-2002	questionnaire	pharmaceutical	NA
RALOXIFENE_HYDROCHLORIDE	RALOXIFENE_HYDROCHLORIDE	2001-2002	questionnaire	pharmaceutical	NA
VENLAFAXINE_HYDROCHLORIDE	VENLAFAXINE_HYDROCHLORIDE	2001-2002	questionnaire	pharmaceutical	NA
FOSINOPRIL_SODIUM	FOSINOPRIL_SODIUM	2001-2002	questionnaire	pharmaceutical	NA
GEMFIBROZIL	GEMFIBROZIL	2001-2002	questionnaire	pharmaceutical	NA
DIVALPROEX_SODIUM	DIVALPROEX_SODIUM	2001-2002	questionnaire	pharmaceutical	NA
IRBESARTAN	IRBESARTAN	2001-2002	questionnaire	pharmaceutical	NA
PHENYTOIN_SODIUM	PHENYTOIN_SODIUM	2001-2002	questionnaire	pharmaceutical	NA
PROPRANOLOL_HYDROCHLORIDE	PROPRANOLOL_HYDROCHLORIDE	2001-2002	questionnaire	pharmaceutical	NA
CYCLOBENZAPRINE_HYDROCHLORIDE	CYCLOBENZAPRINE_HYDROCHLORIDE	2001-2002	questionnaire	pharmaceutical	NA
HYDROCHLOROTHIAZIDE__LOSARTAN_POTASSIUM	HYDROCHLOROTHIAZIDE__LOSARTAN_POTASSIUM	2001-2002	questionnaire	pharmaceutical	NA
MECLIZINE_HYDROCHLORIDE	MECLIZINE_HYDROCHLORIDE	2001-2002	questionnaire	pharmaceutical	NA
TOLTERODINE_TARTRATE	TOLTERODINE_TARTRATE	2001-2002	questionnaire	pharmaceutical	NA
ALBUTEROL	ALBUTEROL	2003-2004	questionnaire	pharmaceutical	NA
LEVOTHYROXINE	LEVOTHYROXINE	2003-2004	questionnaire	pharmaceutical	NA
ATORVASTATIN	ATORVASTATIN	2003-2004	questionnaire	pharmaceutical	NA
LISINOPRIL	LISINOPRIL	2003-2004	questionnaire	pharmaceutical	NA
METOPROLOL	METOPROLOL	2003-2004	questionnaire	pharmaceutical	NA
FUROSEMIDE	FUROSEMIDE	2003-2004	questionnaire	pharmaceutical	NA
ATENOLOL	ATENOLOL	2003-2004	questionnaire	pharmaceutical	NA
HYDROCHLOROTHIAZIDE	HYDROCHLOROTHIAZIDE	2003-2004	questionnaire	pharmaceutical	NA
METFORMIN	METFORMIN	2003-2004	questionnaire	pharmaceutical	NA
SIMVASTATIN	SIMVASTATIN	2003-2004	questionnaire	pharmaceutical	NA
IBUPROFEN	IBUPROFEN	2003-2004	questionnaire	pharmaceutical	NA
AMLODIPINE	AMLODIPINE	2003-2004	questionnaire	pharmaceutical	NA
AMOXICILLIN	AMOXICILLIN	2003-2004	questionnaire	pharmaceutical	NA
ACETAMINOPHEN__HYDROCODONE	ACETAMINOPHEN__HYDROCODONE	2003-2004	questionnaire	pharmaceutical	NA
POTASSIUM_CHLORIDE	POTASSIUM_CHLORIDE	2003-2004	questionnaire	pharmaceutical	NA
CELECOXIB	CELECOXIB	2003-2004	questionnaire	pharmaceutical	NA
RANITIDINE	RANITIDINE	2003-2004	questionnaire	pharmaceutical	NA
LANSOPRAZOLE	LANSOPRAZOLE	2003-2004	questionnaire	pharmaceutical	NA
CETIRIZINE	CETIRIZINE	2003-2004	questionnaire	pharmaceutical	NA
WARFARIN	WARFARIN	2003-2004	questionnaire	pharmaceutical	NA
MONTELUKAST	MONTELUKAST	2003-2004	questionnaire	pharmaceutical	NA
SERTRALINE	SERTRALINE	2003-2004	questionnaire	pharmaceutical	NA
GLIPIZIDE	GLIPIZIDE	2003-2004	questionnaire	pharmaceutical	NA
ALENDRONATE	ALENDRONATE	2003-2004	questionnaire	pharmaceutical	NA
DIGOXIN	DIGOXIN	2003-2004	questionnaire	pharmaceutical	NA
FLUTICASONE__SALMETEROL	FLUTICASONE__SALMETEROL	2003-2004	questionnaire	pharmaceutical	NA
FEXOFENADINE	FEXOFENADINE	2003-2004	questionnaire	pharmaceutical	NA
CLOPIDOGREL	CLOPIDOGREL	2003-2004	questionnaire	pharmaceutical	NA
PANTOPRAZOLE	PANTOPRAZOLE	2003-2004	questionnaire	pharmaceutical	NA
PAROXETINE	PAROXETINE	2003-2004	questionnaire	pharmaceutical	NA
DILTIAZEM	DILTIAZEM	2003-2004	questionnaire	pharmaceutical	NA
HYDROCHLOROTHIAZIDE__TRIAMTERENE	HYDROCHLOROTHIAZIDE__TRIAMTERENE	2003-2004	questionnaire	pharmaceutical	NA
CONJUGATED_ESTROGENS	CONJUGATED_ESTROGENS	2003-2004	questionnaire	pharmaceutical	NA
ROFECOXIB	ROFECOXIB	2003-2004	questionnaire	pharmaceutical	NA
NAPROXEN	NAPROXEN	2003-2004	questionnaire	pharmaceutical	NA
99999	99999	2003-2004	questionnaire	pharmaceutical	NA
FLUTICASONE_NASAL	FLUTICASONE_NASAL	2003-2004	questionnaire	pharmaceutical	NA
GLYBURIDE	GLYBURIDE	2003-2004	questionnaire	pharmaceutical	NA
PRAVASTATIN	PRAVASTATIN	2003-2004	questionnaire	pharmaceutical	NA
ALPRAZOLAM	ALPRAZOLAM	2003-2004	questionnaire	pharmaceutical	NA
VALSARTAN	VALSARTAN	2003-2004	questionnaire	pharmaceutical	NA
ACETAMINOPHEN__PROPOXYPHENE	ACETAMINOPHEN__PROPOXYPHENE	2003-2004	questionnaire	pharmaceutical	NA
GABAPENTIN	GABAPENTIN	2003-2004	questionnaire	pharmaceutical	NA
AMLODIPINE__BENAZEPRIL	AMLODIPINE__BENAZEPRIL	2003-2004	questionnaire	pharmaceutical	NA
ENALAPRIL	ENALAPRIL	2003-2004	questionnaire	pharmaceutical	NA
QUINAPRIL	QUINAPRIL	2003-2004	questionnaire	pharmaceutical	NA
OMEPRAZOLE	OMEPRAZOLE	2003-2004	questionnaire	pharmaceutical	NA
ROSIGLITAZONE	ROSIGLITAZONE	2003-2004	questionnaire	pharmaceutical	NA
VERAPAMIL	VERAPAMIL	2003-2004	questionnaire	pharmaceutical	NA
NIFEDIPINE	NIFEDIPINE	2003-2004	questionnaire	pharmaceutical	NA
PREDNISONE	PREDNISONE	2003-2004	questionnaire	pharmaceutical	NA
FLUOXETINE	FLUOXETINE	2003-2004	questionnaire	pharmaceutical	NA
CLONIDINE	CLONIDINE	2003-2004	questionnaire	pharmaceutical	NA
METHYLPHENIDATE	METHYLPHENIDATE	2003-2004	questionnaire	pharmaceutical	NA
ESCITALOPRAM	ESCITALOPRAM	2003-2004	questionnaire	pharmaceutical	NA
RABEPRAZOLE	RABEPRAZOLE	2003-2004	questionnaire	pharmaceutical	NA
CYCLOBENZAPRINE	CYCLOBENZAPRINE	2003-2004	questionnaire	pharmaceutical	NA
VALDECOXIB	VALDECOXIB	2003-2004	questionnaire	pharmaceutical	NA
VENLAFAXINE	VENLAFAXINE	2003-2004	questionnaire	pharmaceutical	NA
LOSARTAN	LOSARTAN	2003-2004	questionnaire	pharmaceutical	NA
ALLOPURINOL	ALLOPURINOL	2003-2004	questionnaire	pharmaceutical	NA
BUPROPION	BUPROPION	2003-2004	questionnaire	pharmaceutical	NA
DESLORATADINE	DESLORATADINE	2003-2004	questionnaire	pharmaceutical	NA
AMITRIPTYLINE	AMITRIPTYLINE	2003-2004	questionnaire	pharmaceutical	NA
LOVASTATIN	LOVASTATIN	2003-2004	questionnaire	pharmaceutical	NA
CEPHALEXIN	CEPHALEXIN	2003-2004	questionnaire	pharmaceutical	NA
TAMSULOSIN	TAMSULOSIN	2003-2004	questionnaire	pharmaceutical	NA
RAMIPRIL	RAMIPRIL	2003-2004	questionnaire	pharmaceutical	NA
TERAZOSIN	TERAZOSIN	2003-2004	questionnaire	pharmaceutical	NA
NITROGLYCERIN	NITROGLYCERIN	2003-2004	questionnaire	pharmaceutical	NA
PROPRANOLOL	PROPRANOLOL	2003-2004	questionnaire	pharmaceutical	NA
ETHINYL_ESTRADIOL__NORGESTIMATE	ETHINYL_ESTRADIOL__NORGESTIMATE	2003-2004	questionnaire	pharmaceutical	NA
TRAZODONE	TRAZODONE	2003-2004	questionnaire	pharmaceutical	NA
CITALOPRAM	CITALOPRAM	2003-2004	questionnaire	pharmaceutical	NA
PIOGLITAZONE	PIOGLITAZONE	2003-2004	questionnaire	pharmaceutical	NA
ALBUTEROL__IPRATROPIUM	ALBUTEROL__IPRATROPIUM	2003-2004	questionnaire	pharmaceutical	NA
DIAZEPAM	DIAZEPAM	2003-2004	questionnaire	pharmaceutical	NA
AMOXICILLIN__CLAVULANATE	AMOXICILLIN__CLAVULANATE	2003-2004	questionnaire	pharmaceutical	NA
TRAMADOL	TRAMADOL	2003-2004	questionnaire	pharmaceutical	NA
FLUTICASONE	FLUTICASONE	2003-2004	questionnaire	pharmaceutical	NA
ZOLPIDEM	ZOLPIDEM	2003-2004	questionnaire	pharmaceutical	NA
FOSINOPRIL	FOSINOPRIL	2003-2004	questionnaire	pharmaceutical	NA
BENAZEPRIL	BENAZEPRIL	2003-2004	questionnaire	pharmaceutical	NA
ESTRADIOL	ESTRADIOL	2003-2004	questionnaire	pharmaceutical	NA
LORAZEPAM	LORAZEPAM	2003-2004	questionnaire	pharmaceutical	NA
DOXAZOSIN	DOXAZOSIN	2003-2004	questionnaire	pharmaceutical	NA
GLIMEPIRIDE	GLIMEPIRIDE	2003-2004	questionnaire	pharmaceutical	NA
CARVEDILOL	CARVEDILOL	2003-2004	questionnaire	pharmaceutical	NA
SULFAMETHOXAZOLE__TRIMETHOPRIM	SULFAMETHOXAZOLE__TRIMETHOPRIM	2003-2004	questionnaire	pharmaceutical	NA
HYDROXYZINE	HYDROXYZINE	2003-2004	questionnaire	pharmaceutical	NA
ACETAMINOPHEN__CODEINE	ACETAMINOPHEN__CODEINE	2003-2004	questionnaire	pharmaceutical	NA
ISOSORBIDE_MONONITRATE	ISOSORBIDE_MONONITRATE	2003-2004	questionnaire	pharmaceutical	NA
HYDROCHLOROTHIAZIDE__LOSARTAN	HYDROCHLOROTHIAZIDE__LOSARTAN	2003-2004	questionnaire	pharmaceutical	NA
FENOFIBRATE	FENOFIBRATE	2003-2004	questionnaire	pharmaceutical	NA
ETHINYL_ESTRADIOL__NORETHINDRONE	ETHINYL_ESTRADIOL__NORETHINDRONE	2003-2004	questionnaire	pharmaceutical	NA
GLYBURIDE__METFORMIN	GLYBURIDE__METFORMIN	2003-2004	questionnaire	pharmaceutical	NA
INSULIN_ISOPHANE	INSULIN_ISOPHANE	2003-2004	questionnaire	pharmaceutical	NA
AZITHROMYCIN	AZITHROMYCIN	2003-2004	questionnaire	pharmaceutical	NA
LATANOPROST_OPHTHALMIC	LATANOPROST_OPHTHALMIC	2003-2004	questionnaire	pharmaceutical	NA
ALBUTEROL	ALBUTEROL	2005-2006	questionnaire	pharmaceutical	NA
LEVOTHYROXINE	LEVOTHYROXINE	2005-2006	questionnaire	pharmaceutical	NA
ATORVASTATIN	ATORVASTATIN	2005-2006	questionnaire	pharmaceutical	NA
METOPROLOL	METOPROLOL	2005-2006	questionnaire	pharmaceutical	NA
LISINOPRIL	LISINOPRIL	2005-2006	questionnaire	pharmaceutical	NA
HYDROCHLOROTHIAZIDE	HYDROCHLOROTHIAZIDE	2005-2006	questionnaire	pharmaceutical	NA
SIMVASTATIN	SIMVASTATIN	2005-2006	questionnaire	pharmaceutical	NA
METFORMIN	METFORMIN	2005-2006	questionnaire	pharmaceutical	NA
ATENOLOL	ATENOLOL	2005-2006	questionnaire	pharmaceutical	NA
FUROSEMIDE	FUROSEMIDE	2005-2006	questionnaire	pharmaceutical	NA
AMLODIPINE	AMLODIPINE	2005-2006	questionnaire	pharmaceutical	NA
AMOXICILLIN	AMOXICILLIN	2005-2006	questionnaire	pharmaceutical	NA
CETIRIZINE	CETIRIZINE	2005-2006	questionnaire	pharmaceutical	NA
ACETAMINOPHEN__HYDROCODONE	ACETAMINOPHEN__HYDROCODONE	2005-2006	questionnaire	pharmaceutical	NA
MONTELUKAST	MONTELUKAST	2005-2006	questionnaire	pharmaceutical	NA
POTASSIUM_CHLORIDE	POTASSIUM_CHLORIDE	2005-2006	questionnaire	pharmaceutical	NA
WARFARIN	WARFARIN	2005-2006	questionnaire	pharmaceutical	NA
IBUPROFEN	IBUPROFEN	2005-2006	questionnaire	pharmaceutical	NA
ESOMEPRAZOLE	ESOMEPRAZOLE	2005-2006	questionnaire	pharmaceutical	NA
FLUTICASONE__SALMETEROL	FLUTICASONE__SALMETEROL	2005-2006	questionnaire	pharmaceutical	NA
OMEPRAZOLE	OMEPRAZOLE	2005-2006	questionnaire	pharmaceutical	NA
CLOPIDOGREL	CLOPIDOGREL	2005-2006	questionnaire	pharmaceutical	NA
LANSOPRAZOLE	LANSOPRAZOLE	2005-2006	questionnaire	pharmaceutical	NA
SERTRALINE	SERTRALINE	2005-2006	questionnaire	pharmaceutical	NA
GLIPIZIDE	GLIPIZIDE	2005-2006	questionnaire	pharmaceutical	NA
ASPIRIN	ASPIRIN	2005-2006	questionnaire	pharmaceutical	NA
LOVASTATIN	LOVASTATIN	2005-2006	questionnaire	pharmaceutical	NA
FLUTICASONE_NASAL	FLUTICASONE_NASAL	2005-2006	questionnaire	pharmaceutical	NA
ESCITALOPRAM	ESCITALOPRAM	2005-2006	questionnaire	pharmaceutical	NA
FEXOFENADINE	FEXOFENADINE	2005-2006	questionnaire	pharmaceutical	NA
HYDROCHLOROTHIAZIDE__TRIAMTERENE	HYDROCHLOROTHIAZIDE__TRIAMTERENE	2005-2006	questionnaire	pharmaceutical	NA
DILTIAZEM	DILTIAZEM	2005-2006	questionnaire	pharmaceutical	NA
VALSARTAN	VALSARTAN	2005-2006	questionnaire	pharmaceutical	NA
FLUOXETINE	FLUOXETINE	2005-2006	questionnaire	pharmaceutical	NA
DIGOXIN	DIGOXIN	2005-2006	questionnaire	pharmaceutical	NA
NAPROXEN	NAPROXEN	2005-2006	questionnaire	pharmaceutical	NA
METHYLPHENIDATE	METHYLPHENIDATE	2005-2006	questionnaire	pharmaceutical	NA
PIOGLITAZONE	PIOGLITAZONE	2005-2006	questionnaire	pharmaceutical	NA
ALENDRONATE	ALENDRONATE	2005-2006	questionnaire	pharmaceutical	NA
GLYBURIDE	GLYBURIDE	2005-2006	questionnaire	pharmaceutical	NA
PANTOPRAZOLE	PANTOPRAZOLE	2005-2006	questionnaire	pharmaceutical	NA
99999	99999	2005-2006	questionnaire	pharmaceutical	NA
CELECOXIB	CELECOXIB	2005-2006	questionnaire	pharmaceutical	NA
PREDNISONE	PREDNISONE	2005-2006	questionnaire	pharmaceutical	NA
BUPROPION	BUPROPION	2005-2006	questionnaire	pharmaceutical	NA
AMLODIPINE__BENAZEPRIL	AMLODIPINE__BENAZEPRIL	2005-2006	questionnaire	pharmaceutical	NA
MOMETASONE_NASAL	MOMETASONE_NASAL	2005-2006	questionnaire	pharmaceutical	NA
GABAPENTIN	GABAPENTIN	2005-2006	questionnaire	pharmaceutical	NA
TRAZODONE	TRAZODONE	2005-2006	questionnaire	pharmaceutical	NA
AZITHROMYCIN	AZITHROMYCIN	2005-2006	questionnaire	pharmaceutical	NA
ZOLPIDEM	ZOLPIDEM	2005-2006	questionnaire	pharmaceutical	NA
VENLAFAXINE	VENLAFAXINE	2005-2006	questionnaire	pharmaceutical	NA
TAMSULOSIN	TAMSULOSIN	2005-2006	questionnaire	pharmaceutical	NA
NIFEDIPINE	NIFEDIPINE	2005-2006	questionnaire	pharmaceutical	NA
PAROXETINE	PAROXETINE	2005-2006	questionnaire	pharmaceutical	NA
ALLOPURINOL	ALLOPURINOL	2005-2006	questionnaire	pharmaceutical	NA
ALPRAZOLAM	ALPRAZOLAM	2005-2006	questionnaire	pharmaceutical	NA
CARVEDILOL	CARVEDILOL	2005-2006	questionnaire	pharmaceutical	NA
ENALAPRIL	ENALAPRIL	2005-2006	questionnaire	pharmaceutical	NA
CONJUGATED_ESTROGENS	CONJUGATED_ESTROGENS	2005-2006	questionnaire	pharmaceutical	NA
BUDESONIDE	BUDESONIDE	2005-2006	questionnaire	pharmaceutical	NA
FLUTICASONE	FLUTICASONE	2005-2006	questionnaire	pharmaceutical	NA
AMOXICILLIN__CLAVULANATE	AMOXICILLIN__CLAVULANATE	2005-2006	questionnaire	pharmaceutical	NA
INSULIN_GLARGINE	INSULIN_GLARGINE	2005-2006	questionnaire	pharmaceutical	NA
NITROGLYCERIN	NITROGLYCERIN	2005-2006	questionnaire	pharmaceutical	NA
LOSARTAN	LOSARTAN	2005-2006	questionnaire	pharmaceutical	NA
ETHINYL_ESTRADIOL__NORGESTIMATE	ETHINYL_ESTRADIOL__NORGESTIMATE	2005-2006	questionnaire	pharmaceutical	NA
VERAPAMIL	VERAPAMIL	2005-2006	questionnaire	pharmaceutical	NA
PENICILLIN	PENICILLIN	2005-2006	questionnaire	pharmaceutical	NA
EZETIMIBE	EZETIMIBE	2005-2006	questionnaire	pharmaceutical	NA
CYCLOBENZAPRINE	CYCLOBENZAPRINE	2005-2006	questionnaire	pharmaceutical	NA
RAMIPRIL	RAMIPRIL	2005-2006	questionnaire	pharmaceutical	NA
AMITRIPTYLINE	AMITRIPTYLINE	2005-2006	questionnaire	pharmaceutical	NA
AMPHETAMINE__DEXTROAMPHETAMINE	AMPHETAMINE__DEXTROAMPHETAMINE	2005-2006	questionnaire	pharmaceutical	NA
BENAZEPRIL	BENAZEPRIL	2005-2006	questionnaire	pharmaceutical	NA
ROSIGLITAZONE	ROSIGLITAZONE	2005-2006	questionnaire	pharmaceutical	NA
EZETIMIBE__SIMVASTATIN	EZETIMIBE__SIMVASTATIN	2005-2006	questionnaire	pharmaceutical	NA
LORAZEPAM	LORAZEPAM	2005-2006	questionnaire	pharmaceutical	NA
PROPRANOLOL	PROPRANOLOL	2005-2006	questionnaire	pharmaceutical	NA
HYDROCHLOROTHIAZIDE__VALSARTAN	HYDROCHLOROTHIAZIDE__VALSARTAN	2005-2006	questionnaire	pharmaceutical	NA
ESTRADIOL	ESTRADIOL	2005-2006	questionnaire	pharmaceutical	NA
PRAVASTATIN	PRAVASTATIN	2005-2006	questionnaire	pharmaceutical	NA
ETHINYL_ESTRADIOL__NORETHINDRONE	ETHINYL_ESTRADIOL__NORETHINDRONE	2005-2006	questionnaire	pharmaceutical	NA
ACETAMINOPHEN__PROPOXYPHENE	ACETAMINOPHEN__PROPOXYPHENE	2005-2006	questionnaire	pharmaceutical	NA
SPIRONOLACTONE	SPIRONOLACTONE	2005-2006	questionnaire	pharmaceutical	NA
QUINAPRIL	QUINAPRIL	2005-2006	questionnaire	pharmaceutical	NA
CITALOPRAM	CITALOPRAM	2005-2006	questionnaire	pharmaceutical	NA
CEPHALEXIN	CEPHALEXIN	2005-2006	questionnaire	pharmaceutical	NA
POLYETHYLENE_GLYCOL_3350	POLYETHYLENE_GLYCOL_3350	2005-2006	questionnaire	pharmaceutical	NA
TRAMADOL	TRAMADOL	2005-2006	questionnaire	pharmaceutical	NA
ACETAMINOPHEN__CODEINE	ACETAMINOPHEN__CODEINE	2005-2006	questionnaire	pharmaceutical	NA
CLONAZEPAM	CLONAZEPAM	2005-2006	questionnaire	pharmaceutical	NA
ISOSORBIDE_MONONITRATE	ISOSORBIDE_MONONITRATE	2005-2006	questionnaire	pharmaceutical	NA
DOXAZOSIN	DOXAZOSIN	2005-2006	questionnaire	pharmaceutical	NA
OXYBUTYNIN	OXYBUTYNIN	2005-2006	questionnaire	pharmaceutical	NA
ACETAMINOPHEN__OXYCODONE	ACETAMINOPHEN__OXYCODONE	2005-2006	questionnaire	pharmaceutical	NA
RABEPRAZOLE	RABEPRAZOLE	2005-2006	questionnaire	pharmaceutical	NA
FELODIPINE	FELODIPINE	2005-2006	questionnaire	pharmaceutical	NA
GLIMEPIRIDE	GLIMEPIRIDE	2005-2006	questionnaire	pharmaceutical	NA
taking_birth_control	taking_birth_control	2005-2006	questionnaire	pharmaceutical	NA
taking_birth_control	taking_birth_control	2003-2004	questionnaire	pharmaceutical	NA
taking_birth_control	taking_birth_control	2001-2002	questionnaire	pharmaceutical	NA
SMD450	Total # of pipes smoked in home	2003-2004	questionnaire	smoking family	NA
SMD450	Total # of pipes smoked in home	2001-2002	questionnaire	smoking family	NA
SMD450	Total # of pipes smoked in home	1999-2000	questionnaire	smoking family	NA
SMD440	Total # cigars smoked in home	2003-2004	questionnaire	smoking family	NA
SMD440	Total # cigars smoked in home	2001-2002	questionnaire	smoking family	NA
SMD440	Total # cigars smoked in home	1999-2000	questionnaire	smoking family	NA
SMD430	Total # of cigarettes smoked in home	2005-2006	questionnaire	smoking family	NA
SMD430	Total # of cigarettes smoked in home	2003-2004	questionnaire	smoking family	NA
SMD430	Total # of cigarettes smoked in home	2001-2002	questionnaire	smoking family	NA
SMD430	Total # of cigarettes smoked in home	1999-2000	questionnaire	smoking family	NA
SMD415C	Total # of pipe smokers in home	2003-2004	questionnaire	smoking family	NA
SMD415C	Total # of pipe smokers in home	2001-2002	questionnaire	smoking family	NA
SMD415C	Total # of pipe smokers in home	1999-2000	questionnaire	smoking family	NA
SMD415B	Total # of cigar smokers in home	2003-2004	questionnaire	smoking family	NA
SMD415B	Total # of cigar smokers in home	1999-2000	questionnaire	smoking family	NA
SMD415A	Total # of cigarette smokers in home	2005-2006	questionnaire	smoking family	NA
SMD415A	Total # of cigarette smokers in home	2003-2004	questionnaire	smoking family	NA
SMD415A	Total # of cigarette smokers in home	2001-2002	questionnaire	smoking family	NA
SMD415A	Total # of cigarette smokers in home	1999-2000	questionnaire	smoking family	NA
SMD415	Total number of smokers in home	2005-2006	questionnaire	smoking family	NA
SMD415	Total number of smokers in home	2003-2004	questionnaire	smoking family	NA
SMD415	Total number of smokers in home	2001-2002	questionnaire	smoking family	NA
SMD415	Total number of smokers in home	1999-2000	questionnaire	smoking family	NA
SMD410	Does anyone smoke in home?	2005-2006	questionnaire	smoking family	NA
SMD410	Does anyone smoke in home?	2003-2004	questionnaire	smoking family	NA
SMD410	Does anyone smoke in home?	2001-2002	questionnaire	smoking family	NA
SMD410	Does anyone smoke in home?	1999-2000	questionnaire	smoking family	NA
DED038Q	# of times in past yr you had a sunburn	2003-2004	questionnaire	sun exposure	NA
DED038Q	# of times in past yr you had a sunburn	2005-2006	questionnaire	sun exposure	NA
hepa	hepatitis a	1999-2000	questionnaire	immunization	NA
hepa	hepatitis a	2001-2002	questionnaire	immunization	NA
hepa	hepatitis a	2003-2004	questionnaire	immunization	NA
hepa	hepatitis a	2005-2006	questionnaire	immunization	NA
hepb	hepatitis b	1999-2000	questionnaire	immunization	NA
hepb	hepatitis b	2001-2002	questionnaire	immunization	NA
hepb	hepatitis b	2003-2004	questionnaire	immunization	NA
hepb	hepatitis b	2005-2006	questionnaire	immunization	NA
pneu	pneumonia	1999-2000	questionnaire	immunization	NA
pneu	pneumonia	2001-2002	questionnaire	immunization	NA
pneu	pneumonia	2003-2004	questionnaire	immunization	NA
current_loud_noise	current_loud_noise	1999-2000	questionnaire	occupation	NA
current_loud_noise	current_loud_noise	2001-2002	questionnaire	occupation	NA
current_loud_noise	current_loud_noise	2003-2004	questionnaire	occupation	NA
ever_loud_noise_gt3	ever_loud_noise_gt3	1999-2000	questionnaire	occupation	NA
ever_loud_noise_gt3	ever_loud_noise_gt3	2001-2002	questionnaire	occupation	NA
ever_loud_noise_gt3	ever_loud_noise_gt3	2003-2004	questionnaire	occupation	NA
ever_loud_noise_gt3_2	ever_loud_noise_gt3_2	1999-2000	questionnaire	occupation	NA
ever_loud_noise_gt3_2	ever_loud_noise_gt3_2	2001-2002	questionnaire	occupation	NA
ever_loud_noise_gt3_2	ever_loud_noise_gt3_2	2003-2004	questionnaire	occupation	NA
industry_agriculture	industry_agriculture	1999-2000	questionnaire	occupation	NA
industry_agriculture	industry_agriculture	2001-2002	questionnaire	occupation	NA
industry_agriculture	industry_agriculture	2003-2004	questionnaire	occupation	NA
industry_construction	industry_construction	1999-2000	questionnaire	occupation	NA
industry_construction	industry_construction	2001-2002	questionnaire	occupation	NA
industry_construction	industry_construction	2003-2004	questionnaire	occupation	NA
industry_manufacturing	industry_manufacturing	1999-2000	questionnaire	occupation	NA
industry_manufacturing	industry_manufacturing	2001-2002	questionnaire	occupation	NA
industry_manufacturing	industry_manufacturing	2003-2004	questionnaire	occupation	NA
industry_mining	industry_mining	1999-2000	questionnaire	occupation	NA
industry_mining	industry_mining	2001-2002	questionnaire	occupation	NA
industry_mining	industry_mining	2003-2004	questionnaire	occupation	NA
industry_other	industry_other	1999-2000	questionnaire	occupation	NA
industry_other	industry_other	2001-2002	questionnaire	occupation	NA
industry_other	industry_other	2003-2004	questionnaire	occupation	NA
industry_transport	industry_transport	1999-2000	questionnaire	occupation	NA
industry_transport	industry_transport	2001-2002	questionnaire	occupation	NA
industry_transport	industry_transport	2003-2004	questionnaire	occupation	NA
num_months_longest_job	num_months_longest_job	1999-2000	questionnaire	occupation	NA
num_months_longest_job	num_months_longest_job	2001-2002	questionnaire	occupation	NA
num_months_longest_job	num_months_longest_job	2003-2004	questionnaire	occupation	NA
num_months_main_job	num_months_main_job	1999-2000	questionnaire	occupation	NA
num_months_main_job	num_months_main_job	2001-2002	questionnaire	occupation	NA
num_months_main_job	num_months_main_job	2003-2004	questionnaire	occupation	NA
occupation_construction	occupation_construction	1999-2000	questionnaire	occupation	NA
occupation_construction	occupation_construction	2001-2002	questionnaire	occupation	NA
occupation_construction	occupation_construction	2003-2004	questionnaire	occupation	NA
occupation_farm	occupation_farm	1999-2000	questionnaire	occupation	NA
occupation_farm	occupation_farm	2001-2002	questionnaire	occupation	NA
occupation_farm	occupation_farm	2003-2004	questionnaire	occupation	NA
occupation_household	occupation_household	1999-2000	questionnaire	occupation	NA
occupation_household	occupation_household	2001-2002	questionnaire	occupation	NA
occupation_household	occupation_household	2003-2004	questionnaire	occupation	NA
occupation_laborer	occupation_laborer	1999-2000	questionnaire	occupation	NA
occupation_laborer	occupation_laborer	2001-2002	questionnaire	occupation	NA
occupation_laborer	occupation_laborer	2003-2004	questionnaire	occupation	NA
occupation_midmanage	occupation_midmanage	1999-2000	questionnaire	occupation	NA
occupation_midmanage	occupation_midmanage	2001-2002	questionnaire	occupation	NA
occupation_midmanage	occupation_midmanage	2003-2004	questionnaire	occupation	NA
occupation_military	occupation_military	1999-2000	questionnaire	occupation	NA
occupation_military	occupation_military	2001-2002	questionnaire	occupation	NA
occupation_military	occupation_military	2003-2004	questionnaire	occupation	NA
occupation_repair	occupation_repair	1999-2000	questionnaire	occupation	NA
occupation_repair	occupation_repair	2001-2002	questionnaire	occupation	NA
occupation_repair	occupation_repair	2003-2004	questionnaire	occupation	NA
occupation_transport	occupation_transport	1999-2000	questionnaire	occupation	NA
occupation_transport	occupation_transport	2001-2002	questionnaire	occupation	NA
occupation_transport	occupation_transport	2003-2004	questionnaire	occupation	NA
smell_tobacco	smell_tobacco	1999-2000	questionnaire	occupation	NA
smell_tobacco	smell_tobacco	2001-2002	questionnaire	occupation	NA
smell_tobacco	smell_tobacco	2003-2004	questionnaire	occupation	NA
taking_birth_control	taking_birth_control	1999-2000	questionnaire	pharmaceutical	NA
RHQ600	Using estrogen/progestin patches now?	2005-2006	questionnaire	pharmaceutical	NA
RHQ600	Using estrogen/progestin patches now?	2003-2004	questionnaire	pharmaceutical	NA
RHQ600	Using estrogen/progestin patches now?	2001-2002	questionnaire	pharmaceutical	NA
RHQ600	Using estrogen/progestin patches now?	1999-2000	questionnaire	pharmaceutical	NA
RHQ598	Age started estrogen/progestin patches	2005-2006	questionnaire	pharmaceutical	NA
RHQ598	Age started estrogen/progestin patches	2003-2004	questionnaire	pharmaceutical	NA
RHQ598	Age started estrogen/progestin patches	2001-2002	questionnaire	pharmaceutical	NA
RHQ598	Age started estrogen/progestin patches	1999-2000	questionnaire	pharmaceutical	NA
RHQ596	Used estrogen/progestin combo patches?	1999-2000	questionnaire	pharmaceutical	NA
RHQ596	Used estrogen/progestin combo patches?	2005-2006	questionnaire	pharmaceutical	NA
RHQ596	Used estrogen/progestin combo patches?	2003-2004	questionnaire	pharmaceutical	NA
RHQ596	Used estrogen/progestin combo patches?	2001-2002	questionnaire	pharmaceutical	NA
RHQ584	Using estrogen-only patches now?	2001-2002	questionnaire	pharmaceutical	NA
RHQ584	Using estrogen-only patches now?	1999-2000	questionnaire	pharmaceutical	NA
RHQ584	Using estrogen-only patches now?	2005-2006	questionnaire	pharmaceutical	NA
RHQ582	Age started estrogen-only patches	1999-2000	questionnaire	pharmaceutical	NA
RHQ584	Using estrogen-only patches now?	2003-2004	questionnaire	pharmaceutical	NA
RHQ582	Age started estrogen-only patches	2005-2006	questionnaire	pharmaceutical	NA
RHQ582	Age started estrogen-only patches	2003-2004	questionnaire	pharmaceutical	NA
RHQ582	Age started estrogen-only patches	2001-2002	questionnaire	pharmaceutical	NA
RHQ580	Used estrogen-only patches?	2003-2004	questionnaire	pharmaceutical	NA
RHQ580	Used estrogen-only patches?	2001-2002	questionnaire	pharmaceutical	NA
RHQ580	Used estrogen-only patches?	1999-2000	questionnaire	pharmaceutical	NA
RHQ580	Used estrogen-only patches?	2005-2006	questionnaire	pharmaceutical	NA
RHQ574	Taking estrogen/progestin now?	2005-2006	questionnaire	pharmaceutical	NA
RHQ574	Taking estrogen/progestin now?	2003-2004	questionnaire	pharmaceutical	NA
RHQ574	Taking estrogen/progestin now?	2001-2002	questionnaire	pharmaceutical	NA
RHQ574	Taking estrogen/progestin now?	1999-2000	questionnaire	pharmaceutical	NA
RHQ572	Age started estrogen/progestin pills	2001-2002	questionnaire	pharmaceutical	NA
RHQ572	Age started estrogen/progestin pills	1999-2000	questionnaire	pharmaceutical	NA
RHQ572	Age started estrogen/progestin pills	2005-2006	questionnaire	pharmaceutical	NA
RHQ572	Age started estrogen/progestin pills	2003-2004	questionnaire	pharmaceutical	NA
RHQ570	Used estrogen/progestin combo pills	2005-2006	questionnaire	pharmaceutical	NA
RHQ570	Used estrogen/progestin combo pills	2003-2004	questionnaire	pharmaceutical	NA
RHQ570	Used estrogen/progestin combo pills	2001-2002	questionnaire	pharmaceutical	NA
RHQ566	Taking progestin only pills now?	2001-2002	questionnaire	pharmaceutical	NA
RHQ566	Taking progestin only pills now?	1999-2000	questionnaire	pharmaceutical	NA
RHQ566	Taking progestin only pills now?	2005-2006	questionnaire	pharmaceutical	NA
RHQ566	Taking progestin only pills now?	2003-2004	questionnaire	pharmaceutical	NA
RHQ564	Age started progestin-only pills	2003-2004	questionnaire	pharmaceutical	NA
RHQ564	Age started progestin-only pills	2001-2002	questionnaire	pharmaceutical	NA
RHQ564	Age started progestin-only pills	1999-2000	questionnaire	pharmaceutical	NA
RHQ564	Age started progestin-only pills	2005-2006	questionnaire	pharmaceutical	NA
RHQ562	Used hormone pills w/progestin only	2003-2004	questionnaire	pharmaceutical	NA
RHQ562	Used hormone pills w/progestin only	2001-2002	questionnaire	pharmaceutical	NA
RHQ562	Used hormone pills w/progestin only	1999-2000	questionnaire	pharmaceutical	NA
RHQ562	Used hormone pills w/progestin only	2005-2006	questionnaire	pharmaceutical	NA
RHQ558	Taking estrogen-only pills now?	2005-2006	questionnaire	pharmaceutical	NA
RHQ558	Taking estrogen-only pills now?	2003-2004	questionnaire	pharmaceutical	NA
RHQ558	Taking estrogen-only pills now?	2001-2002	questionnaire	pharmaceutical	NA
RHQ558	Taking estrogen-only pills now?	1999-2000	questionnaire	pharmaceutical	NA
RHQ556	Age started estrogen-only pills	2005-2006	questionnaire	pharmaceutical	NA
RHQ556	Age started estrogen-only pills	2003-2004	questionnaire	pharmaceutical	NA
RHQ556	Age started estrogen-only pills	2001-2002	questionnaire	pharmaceutical	NA
RHQ556	Age started estrogen-only pills	1999-2000	questionnaire	pharmaceutical	NA
RHQ554	Use hormone pills w/estrogen only	1999-2000	questionnaire	pharmaceutical	NA
RHQ554	Use hormone pills w/estrogen only	2005-2006	questionnaire	pharmaceutical	NA
RHQ554	Use hormone pills w/estrogen only	2003-2004	questionnaire	pharmaceutical	NA
RHQ554	Use hormone pills w/estrogen only	2001-2002	questionnaire	pharmaceutical	NA
RHQ540	Ever use female hormones?	2001-2002	questionnaire	pharmaceutical	NA
RHQ540	Ever use female hormones?	1999-2000	questionnaire	pharmaceutical	NA
RHQ540	Ever use female hormones?	2005-2006	questionnaire	pharmaceutical	NA
RHQ540	Ever use female hormones?	2003-2004	questionnaire	pharmaceutical	NA
RHQ520	Now use DepoProvera or injectables?	2003-2004	questionnaire	pharmaceutical	NA
RHQ520	Now use DepoProvera or injectables?	2001-2002	questionnaire	pharmaceutical	NA
RHQ520	Now use DepoProvera or injectables?	1999-2000	questionnaire	pharmaceutical	NA
RHQ520	Now use DepoProvera or injectables?	2005-2006	questionnaire	pharmaceutical	NA
RHQ510	Used DepoProvera or injectables?	2005-2006	questionnaire	pharmaceutical	NA
RHQ510	Used DepoProvera or injectables?	2003-2004	questionnaire	pharmaceutical	NA
RHQ510	Used DepoProvera or injectables?	2001-2002	questionnaire	pharmaceutical	NA
RHQ510	Used DepoProvera or injectables?	1999-2000	questionnaire	pharmaceutical	NA
how_long_progestin	how_long_progestin	2005-2006	questionnaire	pharmaceutical	NA
how_long_progestin	how_long_progestin	2003-2004	questionnaire	pharmaceutical	NA
how_long_progestin	how_long_progestin	2001-2002	questionnaire	pharmaceutical	NA
how_long_progestin	how_long_progestin	1999-2000	questionnaire	pharmaceutical	NA
how_long_estrogen_progestin_patch	how_long_estrogen_progestin_patch	2005-2006	questionnaire	pharmaceutical	NA
how_long_estrogen_progestin_patch	how_long_estrogen_progestin_patch	2003-2004	questionnaire	pharmaceutical	NA
how_long_estrogen_progestin_patch	how_long_estrogen_progestin_patch	2001-2002	questionnaire	pharmaceutical	NA
how_long_estrogen_progestin_patch	how_long_estrogen_progestin_patch	1999-2000	questionnaire	pharmaceutical	NA
how_long_estrogen_progestin	how_long_estrogen_progestin	2005-2006	questionnaire	pharmaceutical	NA
how_long_estrogen_progestin	how_long_estrogen_progestin	2003-2004	questionnaire	pharmaceutical	NA
how_long_estrogen_progestin	how_long_estrogen_progestin	2001-2002	questionnaire	pharmaceutical	NA
how_long_estrogen_progestin	how_long_estrogen_progestin	1999-2000	questionnaire	pharmaceutical	NA
how_long_estrogen_patch	how_long_estrogen_patch	2005-2006	questionnaire	pharmaceutical	NA
how_long_estrogen_patch	how_long_estrogen_patch	2003-2004	questionnaire	pharmaceutical	NA
how_long_estrogen_patch	how_long_estrogen_patch	2001-2002	questionnaire	pharmaceutical	NA
how_long_estrogen_patch	how_long_estrogen_patch	1999-2000	questionnaire	pharmaceutical	NA
how_long_estrogen	how_long_estrogen	2005-2006	questionnaire	pharmaceutical	NA
how_long_estrogen	how_long_estrogen	2003-2004	questionnaire	pharmaceutical	NA
how_long_estrogen	how_long_estrogen	2001-2002	questionnaire	pharmaceutical	NA
how_long_estrogen	how_long_estrogen	1999-2000	questionnaire	pharmaceutical	NA
age_stopped_birth_control	age_stopped_birth_control	2005-2006	questionnaire	pharmaceutical	NA
age_stopped_birth_control	age_stopped_birth_control	2003-2004	questionnaire	pharmaceutical	NA
age_stopped_birth_control	age_stopped_birth_control	2001-2002	questionnaire	pharmaceutical	NA
age_stopped_birth_control	age_stopped_birth_control	1999-2000	questionnaire	pharmaceutical	NA
age_started_birth_control	age_started_birth_control	2005-2006	questionnaire	pharmaceutical	NA
age_started_birth_control	age_started_birth_control	2003-2004	questionnaire	pharmaceutical	NA
age_started_birth_control	age_started_birth_control	2001-2002	questionnaire	pharmaceutical	NA
age_started_birth_control	age_started_birth_control	1999-2000	questionnaire	pharmaceutical	NA
SMD650	Avg # cigarettes/day during past 30 days	2003-2004	questionnaire	smoking behavior	NA
SMD650	Avg # cigarettes/day during past 30 days	2005-2006	questionnaire	smoking behavior	NA
SMD641	# days smoked cigs during past 30 days	2003-2004	questionnaire	smoking behavior	NA
SMD641	# days smoked cigs during past 30 days	2005-2006	questionnaire	smoking behavior	NA
SMQ077	How soon after waking do you smoke	2005-2006	questionnaire	smoking behavior	NA
SMQ077	How soon after waking do you smoke	2003-2004	questionnaire	smoking behavior	NA
SMQ077	How soon after waking do you smoke	2001-2002	questionnaire	smoking behavior	NA
SMQ230	Do you now use chewing tobacco	2003-2004	questionnaire	smoking behavior	NA
SMQ230	Do you now use chewing tobacco	2001-2002	questionnaire	smoking behavior	NA
SMQ230	Do you now use chewing tobacco	1999-2000	questionnaire	smoking behavior	NA
SMQ210	Used chewing tobacco 20 times in life	2001-2002	questionnaire	smoking behavior	NA
SMQ210	Used chewing tobacco 20 times in life	1999-2000	questionnaire	smoking behavior	NA
SMQ210	Used chewing tobacco 20 times in life	2003-2004	questionnaire	smoking behavior	NA
SMQ180	Used snuff at least 20 times in life	2001-2002	questionnaire	smoking behavior	NA
SMQ180	Used snuff at least 20 times in life	1999-2000	questionnaire	smoking behavior	NA
SMQ180	Used snuff at least 20 times in life	2003-2004	questionnaire	smoking behavior	NA
SMQ150	Smoked cigars at least 20 times in life	2001-2002	questionnaire	smoking behavior	NA
SMQ150	Smoked cigars at least 20 times in life	1999-2000	questionnaire	smoking behavior	NA
SMQ150	Smoked cigars at least 20 times in life	2003-2004	questionnaire	smoking behavior	NA
SMQ120	Smoked a pipe at least 20 times in life	2003-2004	questionnaire	smoking behavior	NA
SMQ120	Smoked a pipe at least 20 times in life	2001-2002	questionnaire	smoking behavior	NA
SMQ120	Smoked a pipe at least 20 times in life	1999-2000	questionnaire	smoking behavior	NA
SMQ050	number of days since quit	1999-2000	questionnaire	smoking behavior	NA
SMQ050	number of days since quit	2003-2004	questionnaire	smoking behavior	NA
SMQ050	number of days since quit	2001-2002	questionnaire	smoking behavior	NA
SMQ040	Do you now smoke cigarettes...	2005-2006	questionnaire	smoking behavior	NA
SMQ040	Do you now smoke cigarettes...	2001-2002	questionnaire	smoking behavior	NA
SMQ040	Do you now smoke cigarettes...	2003-2004	questionnaire	smoking behavior	NA
SMQ040	Do you now smoke cigarettes...	1999-2000	questionnaire	smoking behavior	NA
SMQ020	Smoked at least 100 cigarettes in life	2003-2004	questionnaire	smoking behavior	NA
SMQ020	Smoked at least 100 cigarettes in life	2001-2002	questionnaire	smoking behavior	NA
SMQ020	Smoked at least 100 cigarettes in life	1999-2000	questionnaire	smoking behavior	NA
SMQ020	Smoked at least 100 cigarettes in life	2005-2006	questionnaire	smoking behavior	NA
SMD235	# years used this chewing tobacco amount	2003-2004	questionnaire	smoking behavior	NA
SMD235	# years used this chewing tobacco amount	2001-2002	questionnaire	smoking behavior	NA
SMD235	# years used this chewing tobacco amount	1999-2000	questionnaire	smoking behavior	NA
SMD220	Age started chewing tobacco regularly	2001-2002	questionnaire	smoking behavior	NA
SMD220	Age started chewing tobacco regularly	1999-2000	questionnaire	smoking behavior	NA
SMD220	Age started chewing tobacco regularly	2003-2004	questionnaire	smoking behavior	NA
SMD190	Age started using snuff regularly	2001-2002	questionnaire	smoking behavior	NA
SMD190	Age started using snuff regularly	1999-2000	questionnaire	smoking behavior	NA
SMD190	Age started using snuff regularly	2003-2004	questionnaire	smoking behavior	NA
SMD160	Age started cigar smoking regularly	2001-2002	questionnaire	smoking behavior	NA
SMD160	Age started cigar smoking regularly	1999-2000	questionnaire	smoking behavior	NA
SMD160	Age started cigar smoking regularly	2003-2004	questionnaire	smoking behavior	NA
SMD130	Age started pipe smoking regularly	2003-2004	questionnaire	smoking behavior	NA
SMD130	Age started pipe smoking regularly	2001-2002	questionnaire	smoking behavior	NA
SMD130	Age started pipe smoking regularly	1999-2000	questionnaire	smoking behavior	NA
SMD100TR	FTC Tar Content	2005-2006	questionnaire	smoking behavior	NA
SMD100TR	FTC Tar Content	2003-2004	questionnaire	smoking behavior	NA
SMD100TR	FTC Tar Content	2001-2002	questionnaire	smoking behavior	NA
SMD100TR	FTC Tar Content	1999-2000	questionnaire	smoking behavior	NA
SMD100NI	FTC Nicotine Content	2003-2004	questionnaire	smoking behavior	NA
SMD100NI	FTC Nicotine Content	2005-2006	questionnaire	smoking behavior	NA
SMD100NI	FTC Nicotine Content	2001-2002	questionnaire	smoking behavior	NA
SMD100NI	FTC Nicotine Content	1999-2000	questionnaire	smoking behavior	NA
SMD100MN	Menthol indicator	2003-2004	questionnaire	smoking behavior	NA
SMD100MN	Menthol indicator	2001-2002	questionnaire	smoking behavior	NA
SMD100MN	Menthol indicator	2005-2006	questionnaire	smoking behavior	NA
SMD100MN	Menthol indicator	1999-2000	questionnaire	smoking behavior	NA
SMD100FL	Filter type	2003-2004	questionnaire	smoking behavior	NA
SMD100FL	Filter type	2001-2002	questionnaire	smoking behavior	NA
SMD100FL	Filter type	2005-2006	questionnaire	smoking behavior	NA
SMD100FL	Filter type	1999-2000	questionnaire	smoking behavior	NA
SMD100CO	FTC Carbon Monoxide Content	2003-2004	questionnaire	smoking behavior	NA
SMD100CO	FTC Carbon Monoxide Content	2001-2002	questionnaire	smoking behavior	NA
SMD100CO	FTC Carbon Monoxide Content	1999-2000	questionnaire	smoking behavior	NA
SMD100CO	FTC Carbon Monoxide Content	2005-2006	questionnaire	smoking behavior	NA
SMD090	Avg # cigarettes/day during past 30 days	2005-2006	questionnaire	smoking behavior	NA
SMD090	Avg # cigarettes/day during past 30 days	2003-2004	questionnaire	smoking behavior	NA
SMD090	Avg # cigarettes/day during past 30 days	2001-2002	questionnaire	smoking behavior	NA
SMD090	Avg # cigarettes/day during past 30 days	1999-2000	questionnaire	smoking behavior	NA
SMD080	# days smoked cigs during past 30 days	1999-2000	questionnaire	smoking behavior	NA
SMD080	# days smoked cigs during past 30 days	2003-2004	questionnaire	smoking behavior	NA
SMD080	# days smoked cigs during past 30 days	2005-2006	questionnaire	smoking behavior	NA
SMD080	# days smoked cigs during past 30 days	2001-2002	questionnaire	smoking behavior	NA
SMD075	How many years smoked this amount	2005-2006	questionnaire	smoking behavior	NA
SMD075	How many years smoked this amount	2003-2004	questionnaire	smoking behavior	NA
SMD075	How many years smoked this amount	2001-2002	questionnaire	smoking behavior	NA
SMD075	How many years smoked this amount	1999-2000	questionnaire	smoking behavior	NA
SMD070	# cigarettes smoked per day now	2001-2002	questionnaire	smoking behavior	NA
SMD070	# cigarettes smoked per day now	2005-2006	questionnaire	smoking behavior	NA
SMD070	# cigarettes smoked per day now	1999-2000	questionnaire	smoking behavior	NA
SMD070	# cigarettes smoked per day now	2003-2004	questionnaire	smoking behavior	NA
SMD057	# cigarettes smoked per day when quit	2003-2004	questionnaire	smoking behavior	NA
SMD057	# cigarettes smoked per day when quit	1999-2000	questionnaire	smoking behavior	NA
SMD057	# cigarettes smoked per day when quit	2001-2002	questionnaire	smoking behavior	NA
SMD057	# cigarettes smoked per day when quit	2005-2006	questionnaire	smoking behavior	NA
SMD055	Age last smoked cigarettes regularly	2001-2002	questionnaire	smoking behavior	NA
SMD055	Age last smoked cigarettes regularly	1999-2000	questionnaire	smoking behavior	NA
SMD055	Age last smoked cigarettes regularly	2003-2004	questionnaire	smoking behavior	NA
SMD030	Age started smoking cigarettes regularly	1999-2000	questionnaire	smoking behavior	NA
SMD030	Age started smoking cigarettes regularly	2003-2004	questionnaire	smoking behavior	NA
SMD030	Age started smoking cigarettes regularly	2001-2002	questionnaire	smoking behavior	NA
SMD030	Age started smoking cigarettes regularly	2005-2006	questionnaire	smoking behavior	NA
anyone_to_help_social	anyone_to_help_social	2001-2002	questionnaire	social support	NA
anyone_to_help_social	anyone_to_help_social	2003-2004	questionnaire	social support	NA
anyone_to_help_social	anyone_to_help_social	2005-2006	questionnaire	social support	NA
first_degree_support	first_degree_support	1999-2000	questionnaire	social support	NA
first_degree_support	first_degree_support	2001-2002	questionnaire	social support	NA
first_degree_support	first_degree_support	2003-2004	questionnaire	social support	NA
first_degree_support	first_degree_support	2005-2006	questionnaire	social support	NA
number_close_friends	number_close_friends	1999-2000	questionnaire	social support	NA
number_close_friends	number_close_friends	2001-2002	questionnaire	social support	NA
number_close_friends	number_close_friends	2003-2004	questionnaire	social support	NA
number_close_friends	number_close_friends	2005-2006	questionnaire	social support	NA
SXQ020	Ever had sexual intercourse	1999-2000	questionnaire	sexual behavior	NA
SXQ020	Ever had sexual intercourse	2001-2002	questionnaire	sexual behavior	NA
SXQ020	Ever had sexual intercourse	2003-2004	questionnaire	sexual behavior	NA
SXQ260	Doctor ever told you had genital herpes	1999-2000	questionnaire	viral infection	NA
SXQ260	Doctor ever told you had genital herpes	2001-2002	questionnaire	viral infection	NA
SXQ260	Doctor ever told you had genital herpes	2003-2004	questionnaire	viral infection	NA
SXQ260	Doctor ever told you had genital herpes	2005-2006	questionnaire	viral infection	NA
SXQ265	Doctor ever told you had genital warts	2005-2006	questionnaire	viral infection	NA
SXQ265	Doctor ever told you had genital warts	1999-2000	questionnaire	viral infection	NA
SXQ265	Doctor ever told you had genital warts	2001-2002	questionnaire	viral infection	NA
SXQ265	Doctor ever told you had genital warts	2003-2004	questionnaire	viral infection	NA
SXQ270	Doctor ever told you had gonorrhea	2003-2004	questionnaire	bacterial infection	NA
SXQ270	Doctor ever told you had gonorrhea	2005-2006	questionnaire	bacterial infection	NA
SXQ270	Doctor ever told you had gonorrhea	1999-2000	questionnaire	bacterial infection	NA
SXQ270	Doctor ever told you had gonorrhea	2001-2002	questionnaire	bacterial infection	NA
SXQ280	Are you circumcised or uncircumcised	2001-2002	questionnaire	sexual behavior	NA
SXQ280	Are you circumcised or uncircumcised	2003-2004	questionnaire	sexual behavior	NA
SXQ280	Are you circumcised or uncircumcised	2005-2006	questionnaire	sexual behavior	NA
SXQ280	Are you circumcised or uncircumcised	1999-2000	questionnaire	sexual behavior	NA
TBQ020	Ever told had positive TB skin test	1999-2000	questionnaire	bacterial infection	NA
TBQ030	Prescribed medicine for preventing TB	1999-2000	questionnaire	bacterial infection	NA
TBQ040	Ever told you had active TB	1999-2000	questionnaire	bacterial infection	NA
TBQ050	Prescribed medicine for active TB	1999-2000	questionnaire	bacterial infection	NA
TBQ060	Lived in household TB sick person	1999-2000	questionnaire	bacterial infection	NA
DR1TALCO	Alcohol (gm)	2005-2006	questionnaire	food component recall	NA
DR1TALCO	Alcohol (gm)	1999-2000	questionnaire	food component recall	NA
DR1TALCO	Alcohol (gm)	2001-2002	questionnaire	food component recall	NA
DR1TALCO	Alcohol (gm)	2003-2004	questionnaire	food component recall	NA
DR1TCAFF	Caffeine (mg)	1999-2000	questionnaire	food component recall	NA
DR1TCAFF	Caffeine (mg)	2001-2002	questionnaire	food component recall	NA
DR1TCAFF	Caffeine (mg)	2003-2004	questionnaire	food component recall	NA
DR1TCAFF	Caffeine (mg)	2005-2006	questionnaire	food component recall	NA
DR1TCALC	Calcium (mg)	2001-2002	questionnaire	food component recall	NA
DR1TCALC	Calcium (mg)	1999-2000	questionnaire	food component recall	NA
DR1TCALC	Calcium (mg)	2003-2004	questionnaire	food component recall	NA
DR1TCALC	Calcium (mg)	2005-2006	questionnaire	food component recall	NA
DR1TCARB	Carbohydrate (gm)	2001-2002	questionnaire	food component recall	NA
DR1TCARB	Carbohydrate (gm)	2005-2006	questionnaire	food component recall	NA
DR1TCARB	Carbohydrate (gm)	2003-2004	questionnaire	food component recall	NA
DR1TCARB	Carbohydrate (gm)	1999-2000	questionnaire	food component recall	NA
DR1TCARO	Carotene	1999-2000	questionnaire	food component recall	NA
DR1TCHOL	Cholesterol (mg)	2005-2006	questionnaire	food component recall	NA
DR1TCHOL	Cholesterol (mg)	2003-2004	questionnaire	food component recall	NA
DR1TCHOL	Cholesterol (mg)	1999-2000	questionnaire	food component recall	NA
DR1TCHOL	Cholesterol (mg)	2001-2002	questionnaire	food component recall	NA
DR1TCOPP	Copper (mg)	1999-2000	questionnaire	food component recall	NA
DR1TCOPP	Copper (mg)	2003-2004	questionnaire	food component recall	NA
DR1TCOPP	Copper (mg)	2001-2002	questionnaire	food component recall	NA
DR1TCOPP	Copper (mg)	2005-2006	questionnaire	food component recall	NA
DR1TFIBE	Dietary fiber (gm)	2001-2002	questionnaire	food component recall	NA
DR1TFIBE	Dietary fiber (gm)	2005-2006	questionnaire	food component recall	NA
DR1TFIBE	Dietary fiber (gm)	2003-2004	questionnaire	food component recall	NA
DR1TFIBE	Dietary fiber (gm)	1999-2000	questionnaire	food component recall	NA
DR1TIRON	Iron (mg)	2001-2002	questionnaire	food component recall	NA
DR1TIRON	Iron (mg)	2005-2006	questionnaire	food component recall	NA
DR1TIRON	Iron (mg)	1999-2000	questionnaire	food component recall	NA
DR1TIRON	Iron (mg)	2003-2004	questionnaire	food component recall	NA
DR1TKCAL	Energy (kcal)	1999-2000	questionnaire	food component recall	NA
DR1TKCAL	Energy (kcal)	2003-2004	questionnaire	food component recall	NA
DR1TKCAL	Energy (kcal)	2005-2006	questionnaire	food component recall	NA
DR1TKCAL	Energy (kcal)	2001-2002	questionnaire	food component recall	NA
DR1TM161	MFA 16:1 (Hexadecenoic) (gm)	1999-2000	questionnaire	food component recall	NA
DR1TM161	MFA 16:1 (Hexadecenoic) (gm)	2001-2002	questionnaire	food component recall	NA
DR1TM161	MFA 16:1 (Hexadecenoic) (gm)	2005-2006	questionnaire	food component recall	NA
DR1TM161	MFA 16:1 (Hexadecenoic) (gm)	2003-2004	questionnaire	food component recall	NA
DR1TM181	MFA 18:1 (Octadecenoic) (gm)	2003-2004	questionnaire	food component recall	NA
DR1TM181	MFA 18:1 (Octadecenoic) (gm)	2005-2006	questionnaire	food component recall	NA
DR1TM181	MFA 18:1 (Octadecenoic) (gm)	2001-2002	questionnaire	food component recall	NA
DR1TM181	MFA 18:1 (Octadecenoic) (gm)	1999-2000	questionnaire	food component recall	NA
DR1TM201	MFA 20:1 (Eicosenoic) (gm)	2005-2006	questionnaire	food component recall	NA
DR1TM201	MFA 20:1 (Eicosenoic) (gm)	1999-2000	questionnaire	food component recall	NA
DR1TM201	MFA 20:1 (Eicosenoic) (gm)	2001-2002	questionnaire	food component recall	NA
DR1TM201	MFA 20:1 (Eicosenoic) (gm)	2003-2004	questionnaire	food component recall	NA
DR1TM221	MFA 22:1 (Docosenoic) (gm)	2005-2006	questionnaire	food component recall	NA
DR1TM221	MFA 22:1 (Docosenoic) (gm)	1999-2000	questionnaire	food component recall	NA
DR1TM221	MFA 22:1 (Docosenoic) (gm)	2003-2004	questionnaire	food component recall	NA
DR1TM221	MFA 22:1 (Docosenoic) (gm)	2001-2002	questionnaire	food component recall	NA
DR1TMAGN	Magnesium (mg)	2001-2002	questionnaire	food component recall	NA
DR1TMAGN	Magnesium (mg)	2005-2006	questionnaire	food component recall	NA
DR1TMAGN	Magnesium (mg)	1999-2000	questionnaire	food component recall	NA
DR1TMAGN	Magnesium (mg)	2003-2004	questionnaire	food component recall	NA
DR1TMFAT	Total monounsaturated fatty acids (gm)	2001-2002	questionnaire	food component recall	NA
DR1TMFAT	Total monounsaturated fatty acids (gm)	2005-2006	questionnaire	food component recall	NA
DR1TMFAT	Total monounsaturated fatty acids (gm)	2003-2004	questionnaire	food component recall	NA
DR1TMFAT	Total monounsaturated fatty acids (gm)	1999-2000	questionnaire	food component recall	NA
DR1TNIAC	Niacin (mg)	1999-2000	questionnaire	food component recall	NA
DR1TNIAC	Niacin (mg)	2001-2002	questionnaire	food component recall	NA
DR1TNIAC	Niacin (mg)	2005-2006	questionnaire	food component recall	NA
DR1TNIAC	Niacin (mg)	2003-2004	questionnaire	food component recall	NA
DR1TP182	PFA 18:2 (Octadecadienoic) (gm)	2003-2004	questionnaire	food component recall	NA
DR1TP182	PFA 18:2 (Octadecadienoic) (gm)	2001-2002	questionnaire	food component recall	NA
DR1TP182	PFA 18:2 (Octadecadienoic) (gm)	2005-2006	questionnaire	food component recall	NA
DR1TP182	PFA 18:2 (Octadecadienoic) (gm)	1999-2000	questionnaire	food component recall	NA
DR1TP183	PFA 18:3 (Octadecatrienoic) (gm)	2003-2004	questionnaire	food component recall	NA
DR1TP183	PFA 18:3 (Octadecatrienoic) (gm)	2005-2006	questionnaire	food component recall	NA
DR1TP183	PFA 18:3 (Octadecatrienoic) (gm)	2001-2002	questionnaire	food component recall	NA
DR1TP183	PFA 18:3 (Octadecatrienoic) (gm)	1999-2000	questionnaire	food component recall	NA
DR1TP184	PFA 18:4 (Octadecatetraenoic) (gm)	1999-2000	questionnaire	food component recall	NA
DR1TP184	PFA 18:4 (Octadecatetraenoic) (gm)	2003-2004	questionnaire	food component recall	NA
DR1TP184	PFA 18:4 (Octadecatetraenoic) (gm)	2005-2006	questionnaire	food component recall	NA
DR1TP184	PFA 18:4 (Octadecatetraenoic) (gm)	2001-2002	questionnaire	food component recall	NA
DR1TP204	PFA 20:4 (Eicosatetraenoic) (gm)	2003-2004	questionnaire	food component recall	NA
DR1TP204	PFA 20:4 (Eicosatetraenoic) (gm)	2005-2006	questionnaire	food component recall	NA
DR1TP204	PFA 20:4 (Eicosatetraenoic) (gm)	1999-2000	questionnaire	food component recall	NA
DR1TP204	PFA 20:4 (Eicosatetraenoic) (gm)	2001-2002	questionnaire	food component recall	NA
DR1TP205	PFA 20:5 (Eicosapentaenoic) (gm)	2001-2002	questionnaire	food component recall	NA
DR1TP205	PFA 20:5 (Eicosapentaenoic) (gm)	2005-2006	questionnaire	food component recall	NA
DR1TP205	PFA 20:5 (Eicosapentaenoic) (gm)	2003-2004	questionnaire	food component recall	NA
DR1TP205	PFA 20:5 (Eicosapentaenoic) (gm)	1999-2000	questionnaire	food component recall	NA
DR1TP225	PFA 22:5 (Docosapentaenoic) (gm)	1999-2000	questionnaire	food component recall	NA
DR1TP225	PFA 22:5 (Docosapentaenoic) (gm)	2005-2006	questionnaire	food component recall	NA
DR1TP225	PFA 22:5 (Docosapentaenoic) (gm)	2001-2002	questionnaire	food component recall	NA
DR1TP225	PFA 22:5 (Docosapentaenoic) (gm)	2003-2004	questionnaire	food component recall	NA
DR1TP226	PFA 22:6 (Docosahexaenoic) (gm)	2001-2002	questionnaire	food component recall	NA
DR1TP226	PFA 22:6 (Docosahexaenoic) (gm)	2005-2006	questionnaire	food component recall	NA
DR1TP226	PFA 22:6 (Docosahexaenoic) (gm)	1999-2000	questionnaire	food component recall	NA
DR1TP226	PFA 22:6 (Docosahexaenoic) (gm)	2003-2004	questionnaire	food component recall	NA
DR1TPFAT	Total polyunsaturated fatty acids (gm)	1999-2000	questionnaire	food component recall	NA
DR1TPFAT	Total polyunsaturated fatty acids (gm)	2001-2002	questionnaire	food component recall	NA
DR1TPFAT	Total polyunsaturated fatty acids (gm)	2005-2006	questionnaire	food component recall	NA
DR1TPFAT	Total polyunsaturated fatty acids (gm)	2003-2004	questionnaire	food component recall	NA
DR1TPHOS	Phosphorus (mg)	2001-2002	questionnaire	food component recall	NA
DR1TPHOS	Phosphorus (mg)	2005-2006	questionnaire	food component recall	NA
DR1TPHOS	Phosphorus (mg)	2003-2004	questionnaire	food component recall	NA
DR1TPHOS	Phosphorus (mg)	1999-2000	questionnaire	food component recall	NA
DR1TPOTA	Potassium (mg)	2005-2006	questionnaire	food component recall	NA
DR1TPOTA	Potassium (mg)	2003-2004	questionnaire	food component recall	NA
DR1TPOTA	Potassium (mg)	1999-2000	questionnaire	food component recall	NA
DR1TPOTA	Potassium (mg)	2001-2002	questionnaire	food component recall	NA
DR1TPROT	Protein (gm)	2003-2004	questionnaire	food component recall	NA
DR1TPROT	Protein (gm)	1999-2000	questionnaire	food component recall	NA
DR1TPROT	Protein (gm)	2005-2006	questionnaire	food component recall	NA
DR1TPROT	Protein (gm)	2001-2002	questionnaire	food component recall	NA
DR1TS040	SFA 4:0 (Butanoic) (gm)	2003-2004	questionnaire	food component recall	NA
DR1TS040	SFA 4:0 (Butanoic) (gm)	1999-2000	questionnaire	food component recall	NA
DR1TS040	SFA 4:0 (Butanoic) (gm)	2001-2002	questionnaire	food component recall	NA
DR1TS040	SFA 4:0 (Butanoic) (gm)	2005-2006	questionnaire	food component recall	NA
DR1TS060	SFA 6:0 (Hexanoic) (gm)	2003-2004	questionnaire	food component recall	NA
DR1TS060	SFA 6:0 (Hexanoic) (gm)	1999-2000	questionnaire	food component recall	NA
DR1TS060	SFA 6:0 (Hexanoic) (gm)	2001-2002	questionnaire	food component recall	NA
DR1TS060	SFA 6:0 (Hexanoic) (gm)	2005-2006	questionnaire	food component recall	NA
DR1TS080	SFA 8:0 (Octanoic) (gm)	1999-2000	questionnaire	food component recall	NA
DR1TS080	SFA 8:0 (Octanoic) (gm)	2001-2002	questionnaire	food component recall	NA
DR1TS080	SFA 8:0 (Octanoic) (gm)	2003-2004	questionnaire	food component recall	NA
DR1TS080	SFA 8:0 (Octanoic) (gm)	2005-2006	questionnaire	food component recall	NA
DR1TS100	SFA 10:0 (Decanoic) (gm)	2005-2006	questionnaire	food component recall	NA
DR1TS100	SFA 10:0 (Decanoic) (gm)	2003-2004	questionnaire	food component recall	NA
DR1TS100	SFA 10:0 (Decanoic) (gm)	2001-2002	questionnaire	food component recall	NA
DR1TS100	SFA 10:0 (Decanoic) (gm)	1999-2000	questionnaire	food component recall	NA
DR1TS120	SFA 12:0 (Dodecanoic) (gm)	2003-2004	questionnaire	food component recall	NA
DR1TS120	SFA 12:0 (Dodecanoic) (gm)	2001-2002	questionnaire	food component recall	NA
DR1TS120	SFA 12:0 (Dodecanoic) (gm)	1999-2000	questionnaire	food component recall	NA
DR1TS120	SFA 12:0 (Dodecanoic) (gm)	2005-2006	questionnaire	food component recall	NA
DR1TS140	SFA 14:0 (Tetradecanoic) (gm)	2005-2006	questionnaire	food component recall	NA
DR1TS140	SFA 14:0 (Tetradecanoic) (gm)	1999-2000	questionnaire	food component recall	NA
DR1TS140	SFA 14:0 (Tetradecanoic) (gm)	2001-2002	questionnaire	food component recall	NA
DR1TS140	SFA 14:0 (Tetradecanoic) (gm)	2003-2004	questionnaire	food component recall	NA
DR1TS160	SFA 16:0 (Hexadecanoic) (gm)	1999-2000	questionnaire	food component recall	NA
DR1TS160	SFA 16:0 (Hexadecanoic) (gm)	2001-2002	questionnaire	food component recall	NA
DR1TS160	SFA 16:0 (Hexadecanoic) (gm)	2003-2004	questionnaire	food component recall	NA
DR1TS160	SFA 16:0 (Hexadecanoic) (gm)	2005-2006	questionnaire	food component recall	NA
DR1TS180	SFA 18:0 (Octadecanoic) (gm)	2005-2006	questionnaire	food component recall	NA
DR1TS180	SFA 18:0 (Octadecanoic) (gm)	2003-2004	questionnaire	food component recall	NA
DR1TS180	SFA 18:0 (Octadecanoic) (gm)	1999-2000	questionnaire	food component recall	NA
DR1TS180	SFA 18:0 (Octadecanoic) (gm)	2001-2002	questionnaire	food component recall	NA
DR1TSELE	Selenium (mcg)	1999-2000	questionnaire	food component recall	NA
DR1TSELE	Selenium (mcg)	2001-2002	questionnaire	food component recall	NA
DR1TSELE	Selenium (mcg)	2003-2004	questionnaire	food component recall	NA
DR1TSELE	Selenium (mcg)	2005-2006	questionnaire	food component recall	NA
DR1TSFAT	Total saturated fatty acids (gm)	2001-2002	questionnaire	food component recall	NA
DR1TSFAT	Total saturated fatty acids (gm)	2005-2006	questionnaire	food component recall	NA
DR1TSFAT	Total saturated fatty acids (gm)	1999-2000	questionnaire	food component recall	NA
DR1TSFAT	Total saturated fatty acids (gm)	2003-2004	questionnaire	food component recall	NA
DR1TTFAT	Total fat (gm)	2005-2006	questionnaire	food component recall	NA
DR1TTFAT	Total fat (gm)	2001-2002	questionnaire	food component recall	NA
DR1TTFAT	Total fat (gm)	2003-2004	questionnaire	food component recall	NA
DR1TTFAT	Total fat (gm)	1999-2000	questionnaire	food component recall	NA
DR1TTHEO	Theobromine (mg)	2003-2004	questionnaire	food component recall	NA
DR1TTHEO	Theobromine (mg)	2001-2002	questionnaire	food component recall	NA
DR1TTHEO	Theobromine (mg)	2005-2006	questionnaire	food component recall	NA
DR1TTHEO	Theobromine (mg)	1999-2000	questionnaire	food component recall	NA
DR1TVAIU	Vitamin A	1999-2000	questionnaire	food component recall	NA
DR1TVARE	Retinol	1999-2000	questionnaire	food component recall	NA
DR1TVB1	Thiamin (Vitamin B1) (mg)	2005-2006	questionnaire	food component recall	NA
DR1TVB1	Thiamin (Vitamin B1) (mg)	2003-2004	questionnaire	food component recall	NA
DR1TVB1	Thiamin (Vitamin B1) (mg)	1999-2000	questionnaire	food component recall	NA
DR1TVB1	Thiamin (Vitamin B1) (mg)	2001-2002	questionnaire	food component recall	NA
DR1TVB12	Vitamin B12 (mcg)	2003-2004	questionnaire	food component recall	NA
DR1TVB12	Vitamin B12 (mcg)	2001-2002	questionnaire	food component recall	NA
DR1TVB12	Vitamin B12 (mcg)	2005-2006	questionnaire	food component recall	NA
DR1TVB12	Vitamin B12 (mcg)	1999-2000	questionnaire	food component recall	NA
DR1TVB2	Riboflavin (Vitamin B2) (mg)	1999-2000	questionnaire	food component recall	NA
DR1TVB2	Riboflavin (Vitamin B2) (mg)	2005-2006	questionnaire	food component recall	NA
DR1TVB2	Riboflavin (Vitamin B2) (mg)	2001-2002	questionnaire	food component recall	NA
DR1TVB2	Riboflavin (Vitamin B2) (mg)	2003-2004	questionnaire	food component recall	NA
DR1TVB6	Vitamin B6 (mg)	1999-2000	questionnaire	food component recall	NA
DR1TVB6	Vitamin B6 (mg)	2005-2006	questionnaire	food component recall	NA
DR1TVB6	Vitamin B6 (mg)	2003-2004	questionnaire	food component recall	NA
DR1TVB6	Vitamin B6 (mg)	2001-2002	questionnaire	food component recall	NA
DR1TVC	Vitamin C (mg)	2001-2002	questionnaire	food component recall	NA
DR1TVC	Vitamin C (mg)	2003-2004	questionnaire	food component recall	NA
DR1TVC	Vitamin C (mg)	1999-2000	questionnaire	food component recall	NA
DR1TVC	Vitamin C (mg)	2005-2006	questionnaire	food component recall	NA
DR1TVE	Vitamin E	1999-2000	questionnaire	food component recall	NA
DR1TWATE	Water	1999-2000	questionnaire	food component recall	NA
DR1TZINC	Zinc (mg)	1999-2000	questionnaire	food component recall	NA
DR1TZINC	Zinc (mg)	2001-2002	questionnaire	food component recall	NA
DR1TZINC	Zinc (mg)	2005-2006	questionnaire	food component recall	NA
DRD320GW	Total plain water drank yesterday (gm)	2001-2002	questionnaire	food component recall	NA
DRD320GW	Total plain water drank yesterday (gm)	1999-2000	questionnaire	food component recall	NA
DRD330GW	Total home tap water drank yesterday(gm)	2001-2002	questionnaire	food component recall	NA
DRD330GW	Total home tap water drank yesterday(gm)	1999-2000	questionnaire	food component recall	NA
DRD340	Shellfish eaten during past 30 days	1999-2000	questionnaire	food component recall	NA
DRD340	Shellfish eaten during past 30 days	2005-2006	questionnaire	food component recall	NA
DRD340	Shellfish eaten during past 30 days	2001-2002	questionnaire	food component recall	NA
DRD340	Shellfish eaten during past 30 days	2003-2004	questionnaire	food component recall	NA
DRD350A	Clams eaten during past 30 days	2005-2006	questionnaire	food component recall	NA
DRD350A	Clams eaten during past 30 days	2003-2004	questionnaire	food component recall	NA
DRD350A	Clams eaten during past 30 days	2001-2002	questionnaire	food component recall	NA
DRD350A	Clams eaten during past 30 days	1999-2000	questionnaire	food component recall	NA
DRD350AQ	# of times clams eaten in past 30 days	2005-2006	questionnaire	food component recall	NA
DRD350AQ	# of times clams eaten in past 30 days	1999-2000	questionnaire	food component recall	NA
DRD350AQ	# of times clams eaten in past 30 days	2003-2004	questionnaire	food component recall	NA
DRD350AQ	# of times clams eaten in past 30 days	2001-2002	questionnaire	food component recall	NA
DRD350B	Crabs eaten during past 30 days	2005-2006	questionnaire	food component recall	NA
DRD350B	Crabs eaten during past 30 days	2001-2002	questionnaire	food component recall	NA
DRD350B	Crabs eaten during past 30 days	2003-2004	questionnaire	food component recall	NA
DRD350B	Crabs eaten during past 30 days	1999-2000	questionnaire	food component recall	NA
DRD350BQ	# of times crabs eaten in past 30 days	1999-2000	questionnaire	food component recall	NA
DRD350BQ	# of times crabs eaten in past 30 days	2003-2004	questionnaire	food component recall	NA
DRD350BQ	# of times crabs eaten in past 30 days	2001-2002	questionnaire	food component recall	NA
DRD350BQ	# of times crabs eaten in past 30 days	2005-2006	questionnaire	food component recall	NA
DRD350C	Crayfish eaten during past 30 days	2001-2002	questionnaire	food component recall	NA
DRD350C	Crayfish eaten during past 30 days	2005-2006	questionnaire	food component recall	NA
DRD350C	Crayfish eaten during past 30 days	2003-2004	questionnaire	food component recall	NA
DRD350C	Crayfish eaten during past 30 days	1999-2000	questionnaire	food component recall	NA
DRD350CQ	# of times crayfish eaten past 30 days	2001-2002	questionnaire	food component recall	NA
DRD350CQ	# of times crayfish eaten past 30 days	2003-2004	questionnaire	food component recall	NA
DRD350CQ	# of times crayfish eaten past 30 days	1999-2000	questionnaire	food component recall	NA
DRD350CQ	# of times crayfish eaten past 30 days	2005-2006	questionnaire	food component recall	NA
DRD350D	Lobsters eaten during past 30 days	2005-2006	questionnaire	food component recall	NA
DRD350D	Lobsters eaten during past 30 days	1999-2000	questionnaire	food component recall	NA
DRD350D	Lobsters eaten during past 30 days	2001-2002	questionnaire	food component recall	NA
DRD350D	Lobsters eaten during past 30 days	2003-2004	questionnaire	food component recall	NA
DRD350DQ	# of times lobsters eaten past 30 days	2001-2002	questionnaire	food component recall	NA
DRD350DQ	# of times lobsters eaten past 30 days	1999-2000	questionnaire	food component recall	NA
DRD350DQ	# of times lobsters eaten past 30 days	2005-2006	questionnaire	food component recall	NA
DRD350DQ	# of times lobsters eaten past 30 days	2003-2004	questionnaire	food component recall	NA
DRD350E	Mussels eaten during past 30 days	2001-2002	questionnaire	food component recall	NA
DRD350E	Mussels eaten during past 30 days	2005-2006	questionnaire	food component recall	NA
DRD350E	Mussels eaten during past 30 days	1999-2000	questionnaire	food component recall	NA
DRD350E	Mussels eaten during past 30 days	2003-2004	questionnaire	food component recall	NA
DRD350EQ	# of times mussels eaten in past 30 days	1999-2000	questionnaire	food component recall	NA
DRD350EQ	# of times mussels eaten in past 30 days	2003-2004	questionnaire	food component recall	NA
DRD350EQ	# of times mussels eaten in past 30 days	2005-2006	questionnaire	food component recall	NA
DRD350EQ	# of times mussels eaten in past 30 days	2001-2002	questionnaire	food component recall	NA
DRD350F	Oysters eaten during past 30 days	2005-2006	questionnaire	food component recall	NA
DRD350F	Oysters eaten during past 30 days	2001-2002	questionnaire	food component recall	NA
DRD350F	Oysters eaten during past 30 days	2003-2004	questionnaire	food component recall	NA
DRD350F	Oysters eaten during past 30 days	1999-2000	questionnaire	food component recall	NA
DRD350FQ	# of times oysters eaten in past 30 days	2001-2002	questionnaire	food component recall	NA
DRD350FQ	# of times oysters eaten in past 30 days	1999-2000	questionnaire	food component recall	NA
DRD350FQ	# of times oysters eaten in past 30 days	2005-2006	questionnaire	food component recall	NA
DRD350FQ	# of times oysters eaten in past 30 days	2003-2004	questionnaire	food component recall	NA
DRD350G	Scallops eaten during past 30 days	1999-2000	questionnaire	food component recall	NA
DRD350G	Scallops eaten during past 30 days	2001-2002	questionnaire	food component recall	NA
DRD350G	Scallops eaten during past 30 days	2005-2006	questionnaire	food component recall	NA
DRD350G	Scallops eaten during past 30 days	2003-2004	questionnaire	food component recall	NA
DRD350GQ	# of times scallops eaten past 30 days	2001-2002	questionnaire	food component recall	NA
DRD350GQ	# of times scallops eaten past 30 days	2003-2004	questionnaire	food component recall	NA
DRD350GQ	# of times scallops eaten past 30 days	1999-2000	questionnaire	food component recall	NA
DRD350GQ	# of times scallops eaten past 30 days	2005-2006	questionnaire	food component recall	NA
DRD350H	Shrimp eaten during past 30 days	1999-2000	questionnaire	food component recall	NA
DRD350H	Shrimp eaten during past 30 days	2001-2002	questionnaire	food component recall	NA
DRD350H	Shrimp eaten during past 30 days	2005-2006	questionnaire	food component recall	NA
DRD350H	Shrimp eaten during past 30 days	2003-2004	questionnaire	food component recall	NA
DRD350HQ	# of times shrimp eaten in past 30 days	2001-2002	questionnaire	food component recall	NA
DRD350HQ	# of times shrimp eaten in past 30 days	1999-2000	questionnaire	food component recall	NA
DRD350HQ	# of times shrimp eaten in past 30 days	2005-2006	questionnaire	food component recall	NA
DRD350HQ	# of times shrimp eaten in past 30 days	2003-2004	questionnaire	food component recall	NA
DRD350I	Other shellfish eaten past 30 days	1999-2000	questionnaire	food component recall	NA
DRD350I	Other shellfish eaten past 30 days	2005-2006	questionnaire	food component recall	NA
DRD350I	Other shellfish eaten past 30 days	2003-2004	questionnaire	food component recall	NA
DRD350I	Other shellfish eaten past 30 days	2001-2002	questionnaire	food component recall	NA
DRD350IQ	# of times other shellfish eaten	2001-2002	questionnaire	food component recall	NA
DRD350IQ	# of times other shellfish eaten	1999-2000	questionnaire	food component recall	NA
DRD350IQ	# of times other shellfish eaten	2005-2006	questionnaire	food component recall	NA
DRD350IQ	# of times other shellfish eaten	2003-2004	questionnaire	food component recall	NA
DRD350J	Other unknown shellfish eaten past 30 d	2005-2006	questionnaire	food component recall	NA
DRD350J	Other unknown shellfish eaten past 30 d	1999-2000	questionnaire	food component recall	NA
DRD350J	Other unknown shellfish eaten past 30 d	2001-2002	questionnaire	food component recall	NA
DRD350J	Other unknown shellfish eaten past 30 d	2003-2004	questionnaire	food component recall	NA
DRD350JQ	# of times other unknown shellfish eaten	2003-2004	questionnaire	food component recall	NA
DRD350JQ	# of times other unknown shellfish eaten	2001-2002	questionnaire	food component recall	NA
DRD350JQ	# of times other unknown shellfish eaten	2005-2006	questionnaire	food component recall	NA
DRD350K	Refused on shellfish eaten past 30 days	1999-2000	questionnaire	food component recall	NA
DRD350K	Refused on shellfish eaten past 30 days	2001-2002	questionnaire	food component recall	NA
DRD360	Fish eaten during past 30 days	2005-2006	questionnaire	food component recall	NA
DRD360	Fish eaten during past 30 days	2001-2002	questionnaire	food component recall	NA
DRD360	Fish eaten during past 30 days	1999-2000	questionnaire	food component recall	NA
DRD360	Fish eaten during past 30 days	2003-2004	questionnaire	food component recall	NA
DRD370A	Breaded fish products eaten past 30 days	2001-2002	questionnaire	food component recall	NA
DRD370A	Breaded fish products eaten past 30 days	1999-2000	questionnaire	food component recall	NA
DRD370A	Breaded fish products eaten past 30 days	2003-2004	questionnaire	food component recall	NA
DRD370A	Breaded fish products eaten past 30 days	2005-2006	questionnaire	food component recall	NA
DRD370AQ	# of times breaded fish products eaten	2003-2004	questionnaire	food component recall	NA
DRD370AQ	# of times breaded fish products eaten	1999-2000	questionnaire	food component recall	NA
DRD370AQ	# of times breaded fish products eaten	2005-2006	questionnaire	food component recall	NA
DRD370AQ	# of times breaded fish products eaten	2001-2002	questionnaire	food component recall	NA
DRD370B	Tuna eaten during past 30 days	2005-2006	questionnaire	food component recall	NA
DRD370B	Tuna eaten during past 30 days	1999-2000	questionnaire	food component recall	NA
DRD370B	Tuna eaten during past 30 days	2001-2002	questionnaire	food component recall	NA
DRD370B	Tuna eaten during past 30 days	2003-2004	questionnaire	food component recall	NA
DRD370BQ	# of times tuna eaten in past 30 days	2003-2004	questionnaire	food component recall	NA
DRD370BQ	# of times tuna eaten in past 30 days	2005-2006	questionnaire	food component recall	NA
DRD370BQ	# of times tuna eaten in past 30 days	1999-2000	questionnaire	food component recall	NA
DRD370BQ	# of times tuna eaten in past 30 days	2001-2002	questionnaire	food component recall	NA
DRD370C	Bass eaten during past 30 days	2003-2004	questionnaire	food component recall	NA
DRD370C	Bass eaten during past 30 days	2001-2002	questionnaire	food component recall	NA
DRD370C	Bass eaten during past 30 days	1999-2000	questionnaire	food component recall	NA
DRD370C	Bass eaten during past 30 days	2005-2006	questionnaire	food component recall	NA
DRD370CQ	# of times bass eaten in past 30 days	2005-2006	questionnaire	food component recall	NA
DRD370CQ	# of times bass eaten in past 30 days	2001-2002	questionnaire	food component recall	NA
DRD370CQ	# of times bass eaten in past 30 days	1999-2000	questionnaire	food component recall	NA
DRD370CQ	# of times bass eaten in past 30 days	2003-2004	questionnaire	food component recall	NA
DRD370D	Catfish eaten during past 30 days	2001-2002	questionnaire	food component recall	NA
DRD370D	Catfish eaten during past 30 days	1999-2000	questionnaire	food component recall	NA
DRD370D	Catfish eaten during past 30 days	2005-2006	questionnaire	food component recall	NA
DRD370D	Catfish eaten during past 30 days	2003-2004	questionnaire	food component recall	NA
DRD370DQ	# of times catfish eaten in past 30 days	2001-2002	questionnaire	food component recall	NA
DRD370DQ	# of times catfish eaten in past 30 days	2005-2006	questionnaire	food component recall	NA
DRD370DQ	# of times catfish eaten in past 30 days	1999-2000	questionnaire	food component recall	NA
DRD370DQ	# of times catfish eaten in past 30 days	2003-2004	questionnaire	food component recall	NA
DRD370E	Cod eaten during past 30 days	1999-2000	questionnaire	food component recall	NA
DRD370E	Cod eaten during past 30 days	2001-2002	questionnaire	food component recall	NA
DRD370E	Cod eaten during past 30 days	2003-2004	questionnaire	food component recall	NA
DRD370E	Cod eaten during past 30 days	2005-2006	questionnaire	food component recall	NA
DRD370EQ	# of times cod eaten in past 30 days	2003-2004	questionnaire	food component recall	NA
DRD370EQ	# of times cod eaten in past 30 days	2001-2002	questionnaire	food component recall	NA
DRD370EQ	# of times cod eaten in past 30 days	1999-2000	questionnaire	food component recall	NA
DRD370EQ	# of times cod eaten in past 30 days	2005-2006	questionnaire	food component recall	NA
DRD370F	Flatfish eaten during past 30 days	1999-2000	questionnaire	food component recall	NA
DRD370F	Flatfish eaten during past 30 days	2001-2002	questionnaire	food component recall	NA
DRD370F	Flatfish eaten during past 30 days	2005-2006	questionnaire	food component recall	NA
DRD370FQ	# of times flatfish eaten past 30 days	2005-2006	questionnaire	food component recall	NA
DRD370FQ	# of times flatfish eaten past 30 days	1999-2000	questionnaire	food component recall	NA
DRD370FQ	# of times flatfish eaten past 30 days	2003-2004	questionnaire	food component recall	NA
DRD370FQ	# of times flatfish eaten past 30 days	2001-2002	questionnaire	food component recall	NA
DRD370G	Haddock eaten during past 30 days	2001-2002	questionnaire	food component recall	NA
DRD370G	Haddock eaten during past 30 days	1999-2000	questionnaire	food component recall	NA
DRD370G	Haddock eaten during past 30 days	2005-2006	questionnaire	food component recall	NA
DRD370G	Haddock eaten during past 30 days	2003-2004	questionnaire	food component recall	NA
DRD370GQ	# of times haddock eaten in past 30 days	2003-2004	questionnaire	food component recall	NA
DRD370GQ	# of times haddock eaten in past 30 days	2001-2002	questionnaire	food component recall	NA
DRD370GQ	# of times haddock eaten in past 30 days	1999-2000	questionnaire	food component recall	NA
DRD370GQ	# of times haddock eaten in past 30 days	2005-2006	questionnaire	food component recall	NA
DRD370H	Mackerel eaten during past 30 days	2001-2002	questionnaire	food component recall	NA
DRD370H	Mackerel eaten during past 30 days	2005-2006	questionnaire	food component recall	NA
DRD370H	Mackerel eaten during past 30 days	1999-2000	questionnaire	food component recall	NA
DRD370H	Mackerel eaten during past 30 days	2003-2004	questionnaire	food component recall	NA
DRD370HQ	# of times mackerel eaten past 30 days	2005-2006	questionnaire	food component recall	NA
DRD370HQ	# of times mackerel eaten past 30 days	2001-2002	questionnaire	food component recall	NA
DRD370HQ	# of times mackerel eaten past 30 days	1999-2000	questionnaire	food component recall	NA
DRD370HQ	# of times mackerel eaten past 30 days	2003-2004	questionnaire	food component recall	NA
DRD370I	Perch eaten during past 30 days	2003-2004	questionnaire	food component recall	NA
DRD370I	Perch eaten during past 30 days	2005-2006	questionnaire	food component recall	NA
DRD370I	Perch eaten during past 30 days	2001-2002	questionnaire	food component recall	NA
DRD370I	Perch eaten during past 30 days	1999-2000	questionnaire	food component recall	NA
DRD370IQ	# of times perch eaten in past 30 days	2001-2002	questionnaire	food component recall	NA
DRD370IQ	# of times perch eaten in past 30 days	1999-2000	questionnaire	food component recall	NA
DRD370IQ	# of times perch eaten in past 30 days	2003-2004	questionnaire	food component recall	NA
DRD370IQ	# of times perch eaten in past 30 days	2005-2006	questionnaire	food component recall	NA
DRD370J	Pike eaten during past 30 days	2003-2004	questionnaire	food component recall	NA
DRD370J	Pike eaten during past 30 days	2005-2006	questionnaire	food component recall	NA
DRD370J	Pike eaten during past 30 days	1999-2000	questionnaire	food component recall	NA
DRD370J	Pike eaten during past 30 days	2001-2002	questionnaire	food component recall	NA
DRD370JQ	# of times pike eaten in past 30 days	1999-2000	questionnaire	food component recall	NA
DRD370JQ	# of times pike eaten in past 30 days	2005-2006	questionnaire	food component recall	NA
DRD370JQ	# of times pike eaten in past 30 days	2003-2004	questionnaire	food component recall	NA
DRD370JQ	# of times pike eaten in past 30 days	2001-2002	questionnaire	food component recall	NA
DRD370K	Pollock eaten during past 30 days	2001-2002	questionnaire	food component recall	NA
DRD370K	Pollock eaten during past 30 days	2003-2004	questionnaire	food component recall	NA
DRD370K	Pollock eaten during past 30 days	1999-2000	questionnaire	food component recall	NA
DRD370K	Pollock eaten during past 30 days	2005-2006	questionnaire	food component recall	NA
DRD370KQ	# of times pollock eaten in past 30 days	2005-2006	questionnaire	food component recall	NA
DRD370KQ	# of times pollock eaten in past 30 days	1999-2000	questionnaire	food component recall	NA
DRD370KQ	# of times pollock eaten in past 30 days	2003-2004	questionnaire	food component recall	NA
DRD370KQ	# of times pollock eaten in past 30 days	2001-2002	questionnaire	food component recall	NA
DRD370L	Porgy eaten during past 30 days	2001-2002	questionnaire	food component recall	NA
DRD370L	Porgy eaten during past 30 days	2003-2004	questionnaire	food component recall	NA
DRD370L	Porgy eaten during past 30 days	1999-2000	questionnaire	food component recall	NA
DRD370LQ	# of times porgy eaten in past 30 days	1999-2000	questionnaire	food component recall	NA
DRD370LQ	# of times porgy eaten in past 30 days	2005-2006	questionnaire	food component recall	NA
DRD370LQ	# of times porgy eaten in past 30 days	2001-2002	questionnaire	food component recall	NA
DRD370LQ	# of times porgy eaten in past 30 days	2003-2004	questionnaire	food component recall	NA
DRD370M	Salmon eaten during past 30 days	2005-2006	questionnaire	food component recall	NA
DRD370M	Salmon eaten during past 30 days	1999-2000	questionnaire	food component recall	NA
DRD370M	Salmon eaten during past 30 days	2003-2004	questionnaire	food component recall	NA
DRD370M	Salmon eaten during past 30 days	2001-2002	questionnaire	food component recall	NA
DRD370MQ	# of times salmon eaten in past 30 days	2001-2002	questionnaire	food component recall	NA
DRD370MQ	# of times salmon eaten in past 30 days	1999-2000	questionnaire	food component recall	NA
DRD370MQ	# of times salmon eaten in past 30 days	2005-2006	questionnaire	food component recall	NA
DRD370MQ	# of times salmon eaten in past 30 days	2003-2004	questionnaire	food component recall	NA
DRD370N	Sardines eaten during past 30 days	2003-2004	questionnaire	food component recall	NA
DRD370N	Sardines eaten during past 30 days	2005-2006	questionnaire	food component recall	NA
DRD370N	Sardines eaten during past 30 days	1999-2000	questionnaire	food component recall	NA
DRD370N	Sardines eaten during past 30 days	2001-2002	questionnaire	food component recall	NA
DRD370NQ	# of times sardines eaten past 30 days	2001-2002	questionnaire	food component recall	NA
DRD370NQ	# of times sardines eaten past 30 days	1999-2000	questionnaire	food component recall	NA
DRD370NQ	# of times sardines eaten past 30 days	2003-2004	questionnaire	food component recall	NA
DRD370NQ	# of times sardines eaten past 30 days	2005-2006	questionnaire	food component recall	NA
DRD370O	Sea bass eaten during past 30 days	2001-2002	questionnaire	food component recall	NA
DRD370O	Sea bass eaten during past 30 days	1999-2000	questionnaire	food component recall	NA
DRD370O	Sea bass eaten during past 30 days	2005-2006	questionnaire	food component recall	NA
DRD370O	Sea bass eaten during past 30 days	2003-2004	questionnaire	food component recall	NA
DRD370OQ	# of times sea bass eaten past 30 days	2005-2006	questionnaire	food component recall	NA
DRD370OQ	# of times sea bass eaten past 30 days	2003-2004	questionnaire	food component recall	NA
DRD370OQ	# of times sea bass eaten past 30 days	1999-2000	questionnaire	food component recall	NA
DRD370OQ	# of times sea bass eaten past 30 days	2001-2002	questionnaire	food component recall	NA
DRD370P	Shark eaten during past 30 days	1999-2000	questionnaire	food component recall	NA
DRD370P	Shark eaten during past 30 days	2001-2002	questionnaire	food component recall	NA
DRD370P	Shark eaten during past 30 days	2003-2004	questionnaire	food component recall	NA
DRD370P	Shark eaten during past 30 days	2005-2006	questionnaire	food component recall	NA
DRD370PQ	# of times shark eaten in past 30 days	2003-2004	questionnaire	food component recall	NA
DRD370PQ	# of times shark eaten in past 30 days	2001-2002	questionnaire	food component recall	NA
DRD370PQ	# of times shark eaten in past 30 days	2005-2006	questionnaire	food component recall	NA
DRD370PQ	# of times shark eaten in past 30 days	1999-2000	questionnaire	food component recall	NA
DRD370Q	Swordfish eaten during past 30 days	2003-2004	questionnaire	food component recall	NA
DRD370Q	Swordfish eaten during past 30 days	2001-2002	questionnaire	food component recall	NA
DRD370Q	Swordfish eaten during past 30 days	1999-2000	questionnaire	food component recall	NA
DRD370Q	Swordfish eaten during past 30 days	2005-2006	questionnaire	food component recall	NA
DRD370QQ	# of times swordfish eaten past 30 days	2005-2006	questionnaire	food component recall	NA
DRD370QQ	# of times swordfish eaten past 30 days	2003-2004	questionnaire	food component recall	NA
DRD370QQ	# of times swordfish eaten past 30 days	1999-2000	questionnaire	food component recall	NA
DRD370QQ	# of times swordfish eaten past 30 days	2001-2002	questionnaire	food component recall	NA
DRD370R	Trout eaten during past 30 days	2005-2006	questionnaire	food component recall	NA
DRD370R	Trout eaten during past 30 days	2003-2004	questionnaire	food component recall	NA
DRD370R	Trout eaten during past 30 days	2001-2002	questionnaire	food component recall	NA
DRD370R	Trout eaten during past 30 days	1999-2000	questionnaire	food component recall	NA
DRD370RQ	# of times trout eaten in past 30 days	2005-2006	questionnaire	food component recall	NA
DRD370RQ	# of times trout eaten in past 30 days	2003-2004	questionnaire	food component recall	NA
DRD370RQ	# of times trout eaten in past 30 days	2001-2002	questionnaire	food component recall	NA
DRD370RQ	# of times trout eaten in past 30 days	1999-2000	questionnaire	food component recall	NA
DRD370S	Walleye eaten during past 30 days	2005-2006	questionnaire	food component recall	NA
DRD370S	Walleye eaten during past 30 days	1999-2000	questionnaire	food component recall	NA
DRD370S	Walleye eaten during past 30 days	2003-2004	questionnaire	food component recall	NA
DRD370S	Walleye eaten during past 30 days	2001-2002	questionnaire	food component recall	NA
DRD370SQ	# of times walleye eaten in past 30 days	1999-2000	questionnaire	food component recall	NA
DRD370SQ	# of times walleye eaten in past 30 days	2001-2002	questionnaire	food component recall	NA
DRD370SQ	# of times walleye eaten in past 30 days	2005-2006	questionnaire	food component recall	NA
DRD370SQ	# of times walleye eaten in past 30 days	2003-2004	questionnaire	food component recall	NA
DRD370T	Other fish eaten during past 30 days	1999-2000	questionnaire	food component recall	NA
DRD370T	Other fish eaten during past 30 days	2003-2004	questionnaire	food component recall	NA
DRD370T	Other fish eaten during past 30 days	2001-2002	questionnaire	food component recall	NA
DRD370T	Other fish eaten during past 30 days	2005-2006	questionnaire	food component recall	NA
DRD370TQ	# of times other fish eaten past 30 days	1999-2000	questionnaire	food component recall	NA
DRD370TQ	# of times other fish eaten past 30 days	2001-2002	questionnaire	food component recall	NA
DRD370TQ	# of times other fish eaten past 30 days	2003-2004	questionnaire	food component recall	NA
DRD370TQ	# of times other fish eaten past 30 days	2005-2006	questionnaire	food component recall	NA
DRD370U	Other unknown fish eaten in past 30 days	2001-2002	questionnaire	food component recall	NA
DRD370U	Other unknown fish eaten in past 30 days	2005-2006	questionnaire	food component recall	NA
DRD370U	Other unknown fish eaten in past 30 days	2003-2004	questionnaire	food component recall	NA
DRD370U	Other unknown fish eaten in past 30 days	1999-2000	questionnaire	food component recall	NA
DRD370UQ	# of times other unknown fish eaten	1999-2000	questionnaire	food component recall	NA
DRD370UQ	# of times other unknown fish eaten	2005-2006	questionnaire	food component recall	NA
DRD370UQ	# of times other unknown fish eaten	2003-2004	questionnaire	food component recall	NA
DRD370UQ	# of times other unknown fish eaten	2001-2002	questionnaire	food component recall	NA
DRD370V	Refused on fish eaten past 30 days	2001-2002	questionnaire	food component recall	NA
DRD370V	Refused on fish eaten past 30 days	1999-2000	questionnaire	food component recall	NA
DRDTSODI	Sodium (mg)	1999-2000	questionnaire	food component recall	NA
DRDTSODI	Sodium (mg)	2001-2002	questionnaire	food component recall	NA
DBD100	How often add salt to food at table	2001-2002	questionnaire	food component recall	NA
DBD100	How often add salt to food at table	2005-2006	questionnaire	food component recall	NA
DBD100	How often add salt to food at table	2003-2004	questionnaire	food component recall	NA
DBQ095	Type of salt used at table	2001-2002	questionnaire	food component recall	NA
DR1TACAR	Alpha-carotene (mcg)	2001-2002	questionnaire	food component recall	NA
DR1TACAR	Alpha-carotene (mcg)	2003-2004	questionnaire	food component recall	NA
DR1TACAR	Alpha-carotene (mcg)	2005-2006	questionnaire	food component recall	NA
DR1TATOC	Vitamin E as alpha-tocopherol (mg)	2001-2002	questionnaire	food component recall	NA
DR1TATOC	Vitamin E as alpha-tocopherol (mg)	2005-2006	questionnaire	food component recall	NA
DR1TATOC	Vitamin E as alpha-tocopherol (mg)	2003-2004	questionnaire	food component recall	NA
DR1TBCAR	Beta-carotene (mcg)	2001-2002	questionnaire	food component recall	NA
DR1TBCAR	Beta-carotene (mcg)	2003-2004	questionnaire	food component recall	NA
DR1TBCAR	Beta-carotene (mcg)	2005-2006	questionnaire	food component recall	NA
DR1TCRYP	Beta-cryptoxanthin (mcg)	2001-2002	questionnaire	food component recall	NA
DR1TCRYP	Beta-cryptoxanthin (mcg)	2003-2004	questionnaire	food component recall	NA
DR1TCRYP	Beta-cryptoxanthin (mcg)	2005-2006	questionnaire	food component recall	NA
DR1TFA	Folic acid (mcg)	2003-2004	questionnaire	food component recall	NA
DR1TFA	Folic acid (mcg)	2001-2002	questionnaire	food component recall	NA
DR1TFA	Folic acid (mcg)	2005-2006	questionnaire	food component recall	NA
DR1TFDFE	Folate, DFE (mcg)	2003-2004	questionnaire	food component recall	NA
DR1TFDFE	Folate, DFE (mcg)	2001-2002	questionnaire	food component recall	NA
DR1TFDFE	Folate, DFE (mcg)	2005-2006	questionnaire	food component recall	NA
DR1TFF	Food folate (mcg)	2005-2006	questionnaire	food component recall	NA
DR1TFF	Food folate (mcg)	2003-2004	questionnaire	food component recall	NA
DR1TFF	Food folate (mcg)	2001-2002	questionnaire	food component recall	NA
DR1TLYCO	Lycopene (mcg)	2001-2002	questionnaire	food component recall	NA
DR1TLYCO	Lycopene (mcg)	2003-2004	questionnaire	food component recall	NA
DR1TLYCO	Lycopene (mcg)	2005-2006	questionnaire	food component recall	NA
DR1TLZ	Lutein + zeaxanthin (mcg)	2005-2006	questionnaire	food component recall	NA
DR1TLZ	Lutein + zeaxanthin (mcg)	2001-2002	questionnaire	food component recall	NA
DR1TLZ	Lutein + zeaxanthin (mcg)	2003-2004	questionnaire	food component recall	NA
DR1TMOIS	Moisture (gm)	2003-2004	questionnaire	food component recall	NA
DR1TMOIS	Moisture (gm)	2005-2006	questionnaire	food component recall	NA
DR1TRET	Retinol (mcg)	2003-2004	questionnaire	food component recall	NA
DR1TRET	Retinol (mcg)	2001-2002	questionnaire	food component recall	NA
DR1TRET	Retinol (mcg)	2005-2006	questionnaire	food component recall	NA
DR1TSUGR	Total sugars (gm)	2001-2002	questionnaire	food component recall	NA
DR1TSUGR	Total sugars (gm)	2003-2004	questionnaire	food component recall	NA
DR1TSUGR	Total sugars (gm)	2005-2006	questionnaire	food component recall	NA
DR1TVARA	Vitamin A, RAE (mcg)	2003-2004	questionnaire	food component recall	NA
DR1TVARA	Vitamin A, RAE (mcg)	2005-2006	questionnaire	food component recall	NA
DR1TVARA	Vitamin A, RAE (mcg)	2001-2002	questionnaire	food component recall	NA
DR1TVK	Vitamin K (mcg)	2005-2006	questionnaire	food component recall	NA
DR1TVK	Vitamin K (mcg)	2001-2002	questionnaire	food component recall	NA
DR1TVK	Vitamin K (mcg)	2003-2004	questionnaire	food component recall	NA
DRDCWATR	Plain carbonated water (gm)	2001-2002	questionnaire	food component recall	NA
DRDDRSTZ	Dietary recall status	2001-2002	questionnaire	food component recall	NA
DRDRESP	Main respondent for the interview	2001-2002	questionnaire	food component recall	NA
lite_salt	Light Salt	2005-2006	questionnaire	food component recall	NA
lite_salt	Light Salt	2001-2002	questionnaire	food component recall	NA
lite_salt	Light Salt	2003-2004	questionnaire	food component recall	NA
no_salt	No Salt	2005-2006	questionnaire	food component recall	NA
no_salt	No Salt	2001-2002	questionnaire	food component recall	NA
no_salt	No Salt	2003-2004	questionnaire	food component recall	NA
ordinary_salt	Ordinary Salt	2005-2006	questionnaire	food component recall	NA
ordinary_salt	Ordinary Salt	2001-2002	questionnaire	food component recall	NA
ordinary_salt	Ordinary Salt	2003-2004	questionnaire	food component recall	NA
salt_substitute	Salt Substitute	2005-2006	questionnaire	food component recall	NA
salt_substitute	Salt Substitute	2003-2004	questionnaire	food component recall	NA
salt_substitute	Salt Substitute	2001-2002	questionnaire	food component recall	NA
community_supply	Community Water Supply	2005-2006	questionnaire	food component recall	NA
community_supply	Community Water Supply	2003-2004	questionnaire	food component recall	NA
DR1_320	Total plain water drank yesterday (gm)	2003-2004	questionnaire	food component recall	NA
DR1_320	Total plain water drank yesterday (gm)	2005-2006	questionnaire	food component recall	NA
DR1_330	Total tap water drank yesterday (gm)	2003-2004	questionnaire	food component recall	NA
DR1BWATR	Total bottled water drank yesterday (gm)	2005-2006	questionnaire	food component recall	NA
DR1BWATR	Total bottled water drank yesterday (gm)	2003-2004	questionnaire	food component recall	NA
DR1CWATR	Plain carbonated water (gm)	2003-2004	questionnaire	food component recall	NA
DR1TATOA	Added alpha-tocopherol (Vitamin E) (mg)	2003-2004	questionnaire	food component recall	NA
DR1TATOA	Added alpha-tocopherol (Vitamin E) (mg)	2005-2006	questionnaire	food component recall	NA
DR1TB12A	Added vitamin B12 (mcg)	2003-2004	questionnaire	food component recall	NA
DR1TB12A	Added vitamin B12 (mcg)	2005-2006	questionnaire	food component recall	NA
DR1TSODI	Sodium (mg)	2005-2006	questionnaire	food component recall	NA
DR1TSODI	Sodium (mg)	2003-2004	questionnaire	food component recall	NA
DRDSDT1	Weight Loss Diet	2005-2006	questionnaire	food component recall	NA
DRDSDT1	Weight Loss Diet	2003-2004	questionnaire	food component recall	NA
DRDSDT2	Low fat Diet	2005-2006	questionnaire	food component recall	NA
DRDSDT2	Low fat Diet	2003-2004	questionnaire	food component recall	NA
DRDSDT3	Low salt Diet	2003-2004	questionnaire	food component recall	NA
DRDSDT3	Low salt Diet	2005-2006	questionnaire	food component recall	NA
DRDSDT4	Sugarfree Diet	2005-2006	questionnaire	food component recall	NA
DRDSDT4	Sugarfree Diet	2003-2004	questionnaire	food component recall	NA
DRDSDT5	Low fiber Diet	2005-2006	questionnaire	food component recall	NA
DRDSDT5	Low fiber Diet	2003-2004	questionnaire	food component recall	NA
DRDSDT6	High fiber Diet	2005-2006	questionnaire	food component recall	NA
DRDSDT7	Diabetic Diet	2005-2006	questionnaire	food component recall	NA
DRDSDT7	Diabetic Diet	2003-2004	questionnaire	food component recall	NA
DRDSDT8	Weight-gain Diet	2005-2006	questionnaire	food component recall	NA
DRDSDT8	Weight-gain Diet	2003-2004	questionnaire	food component recall	NA
spring_water	Spring Water	2005-2006	questionnaire	food component recall	NA
spring_water	Spring Water	2003-2004	questionnaire	food component recall	NA
well_water	Well Water	2005-2006	questionnaire	food component recall	NA
well_water	Well Water	2003-2004	questionnaire	food component recall	NA
DR1_330Z	Total tap water drank yesterday (gm)	2005-2006	questionnaire	food component recall	NA
DR1TCHL	Total choline (mg)	2005-2006	questionnaire	food component recall	NA
LBXSCR	Creatinine (mg/dL)	1999-2000	laboratory	biochemistry	NA
LBXSCR	Creatinine (mg/dL)	2001-2002	laboratory	biochemistry	NA
LBXSCR	Creatinine (mg/dL)	2005-2006	laboratory	biochemistry	NA
LBXSCR	Creatinine (mg/dL)	2003-2004	laboratory	biochemistry	NA
BMXWT	Weight (kg)	2003-2004	examination	body measures	NA
BMXRECUM	Recumbent Length (cm)	2003-2004	examination	body measures	NA
BMXHEAD	Head Circumference (cm)	2003-2004	examination	body measures	NA
BMXHT	Standing Height (cm)	2003-2004	examination	body measures	NA
BMXBMI	Body Mass Index (kg/m**2)	2003-2004	examination	body measures	NA
BMXLEG	Upper Leg Length (cm)	2003-2004	examination	body measures	NA
BMXCALF	Maximal Calf Circumference (cm)	2003-2004	examination	body measures	NA
BMXWAIST	Waist Circumference (cm)	2003-2004	examination	body measures	NA
BMXTHICR	Thigh Circumference (cm)	2003-2004	examination	body measures	NA
BMXTRI	Triceps Skinfold (mm)	2003-2004	examination	body measures	NA
BMXSUB	Subscapular Skinfold (mm)	2003-2004	examination	body measures	NA
BPXCHR	60 sec HR (30 sec HR * 2)	2003-2004	examination	blood pressure	NA
BMXWT	Weight (kg)	2005-2006	examination	body measures	NA
BMXRECUM	Recumbent Length (cm)	2005-2006	examination	body measures	NA
BMXHEAD	Head Circumference (cm)	2005-2006	examination	body measures	NA
BMXHT	Standing Height (cm)	2005-2006	examination	body measures	NA
BMXBMI	Body Mass Index (kg/m**2)	2005-2006	examination	body measures	NA
BMXLEG	Upper Leg Length (cm)	2005-2006	examination	body measures	NA
BMXCALF	Maximal Calf Circumference (cm)	2005-2006	examination	body measures	NA
BMXWAIST	Waist Circumference (cm)	2005-2006	examination	body measures	NA
BMXTHICR	Thigh Circumference (cm)	2005-2006	examination	body measures	NA
BMXTRI	Triceps Skinfold (mm)	2005-2006	examination	body measures	NA
BMXSUB	Subscapular Skinfold (mm)	2005-2006	examination	body measures	NA
BPXCHR	60 sec HR (30 sec HR * 2)	2005-2006	examination	blood pressure	NA
BMXWT	Weight (kg)	2001-2002	examination	body measures	NA
BMXRECUM	Recumbent Length (cm)	2001-2002	examination	body measures	NA
BMXHEAD	Head Circumference (cm)	2001-2002	examination	body measures	NA
BMXHT	Standing Height (cm)	2001-2002	examination	body measures	NA
BMXBMI	Body Mass Index (kg/m**2)	2001-2002	examination	body measures	NA
BMXLEG	Upper Leg Length (cm)	2001-2002	examination	body measures	NA
BMXCALF	Maximal Calf Circumference (cm)	2001-2002	examination	body measures	NA
BMXWAIST	Waist Circumference (cm)	2001-2002	examination	body measures	NA
BMXTHICR	Thigh Circumference (cm)	2001-2002	examination	body measures	NA
BMXTRI	Triceps Skinfold (mm)	2001-2002	examination	body measures	NA
BMXSUB	Subscapular Skinfold (mm)	2001-2002	examination	body measures	NA
BPXCHR	60 sec HR (30 sec HR * 2)	2001-2002	examination	blood pressure	NA
CVDVOMAX	Predicted VO2max (ml/kg/min)	2001-2002	examination	physical fitness	NA
CVDESVO2	Estimated VO2max (ml/kg/min)	2001-2002	examination	physical fitness	NA
BMXRECUM	Recumbent Length (cm)	1999-2000	examination	body measures	NA
BMXHEAD	Head Circumference (cm)	1999-2000	examination	body measures	NA
BMXHT	Standing Height (cm)	1999-2000	examination	body measures	NA
BMXBMI	Body Mass Index (kg/m**2)	1999-2000	examination	body measures	NA
BMXLEG	Upper Leg Length (cm)	1999-2000	examination	body measures	NA
BMXCALF	Maximal Calf Circumference (cm)	1999-2000	examination	body measures	NA
BMXWAIST	Waist Circumference (cm)	1999-2000	examination	body measures	NA
BMXTHICR	Thigh Circumference (cm)	1999-2000	examination	body measures	NA
BMXTRI	Triceps Skinfold (mm)	1999-2000	examination	body measures	NA
BMXSUB	Subscapular Skinfold (mm)	1999-2000	examination	body measures	NA
BPXCHR	60 sec HR (30 sec HR * 2)	1999-2000	examination	blood pressure	NA
CVDVOMAX	Predicted VO2max (ml/kg/min)	1999-2000	examination	physical fitness	NA
CVDESVO2	Estimated VO2max (ml/kg/min)	1999-2000	examination	physical fitness	NA
CVDVOMAX	Predicted VO2max (ml/kg/min)	2003-2004	examination	physical fitness	NA
CVDESVO2	Estimated VO2max (ml/kg/min)	2003-2004	examination	physical fitness	NA
MSYS	mean systolic	1999-2000	examination	blood pressure	NA
MSYS	mean systolic	2001-2002	examination	blood pressure	NA
MSYS	mean systolic	2003-2004	examination	blood pressure	NA
MSYS	mean systolic	2005-2006	examination	blood pressure	NA
MDIS	mean diastolic	1999-2000	examination	blood pressure	NA
MDIS	mean diastolic	2001-2002	examination	blood pressure	NA
MDIS	mean diastolic	2003-2004	examination	blood pressure	NA
MDIS	mean diastolic	2005-2006	examination	blood pressure	NA
CVDS1HR	Stage 1 heart rate (per min)	2001-2002	examination	physical fitness	NA
CVDS1SY	Stage 1 systolic BP (mm Hg)	2001-2002	examination	physical fitness	NA
CVDS1DI	Stage 1 diastolic BP (mm Hg)	2001-2002	examination	physical fitness	NA
CVDS2HR	Stage 2 heart rate (per min)	2001-2002	examination	physical fitness	NA
CVDS2SY	Stage 2 systolic BP (mm Hg)	2001-2002	examination	physical fitness	NA
CVDS2DI	Stage 2 diastolic BP (mm Hg)	2001-2002	examination	physical fitness	NA
CVDR1HR	Recovery 1 heart rate (per min)	2001-2002	examination	physical fitness	NA
CVDR1SY	Recovery 1 systolic BP (mm Hg)	2001-2002	examination	physical fitness	NA
CVDR1DI	Recovery 1 diastolic BP (mm Hg)	2001-2002	examination	physical fitness	NA
CVDR2HR	Recovery 2 heart rate (per min)	2001-2002	examination	physical fitness	NA
CVDR2SY	Recovery 2 systolic BP (mm Hg)	2001-2002	examination	physical fitness	NA
CVDR2DI	Recovery 2 diastolic BP (mm Hg)	2001-2002	examination	physical fitness	NA
CVDS1HR	Stage 1 heart rate (per min)	1999-2000	examination	physical fitness	NA
CVDS1SY	Stage 1 systolic BP (mm Hg)	1999-2000	examination	physical fitness	NA
CVDS1DI	Stage 1 diastolic BP (mm Hg)	1999-2000	examination	physical fitness	NA
CVDS2HR	Stage 2 heart rate (per min)	1999-2000	examination	physical fitness	NA
CVDS2SY	Stage 2 systolic BP (mm Hg)	1999-2000	examination	physical fitness	NA
CVDS2DI	Stage 2 diastolic BP (mm Hg)	1999-2000	examination	physical fitness	NA
CVDR1HR	Recovery 1 heart rate (per min)	1999-2000	examination	physical fitness	NA
CVDR1SY	Recovery 1 systolic BP (mm Hg)	1999-2000	examination	physical fitness	NA
CVDR1DI	Recovery 1 diastolic BP (mm Hg)	1999-2000	examination	physical fitness	NA
CVDR2HR	Recovery 2 heart rate (per min)	1999-2000	examination	physical fitness	NA
CVDR2SY	Recovery 2 systolic BP (mm Hg)	1999-2000	examination	physical fitness	NA
CVDR2DI	Recovery 2 diastolic BP (mm Hg)	1999-2000	examination	physical fitness	NA
CVDS1DI	Stage 1 diastolic BP (mm Hg)	2003-2004	examination	physical fitness	NA
CVDS1SY	Stage 1 systolic BP (mm Hg)	2003-2004	examination	physical fitness	NA
CVDS1HR	Stage 1 heart rate (per min)	2003-2004	examination	physical fitness	NA
CVDS2HR	Stage 2 heart rate (per min)	2003-2004	examination	physical fitness	NA
CVDS2SY	Stage 2 systolic BP (mm Hg)	2003-2004	examination	physical fitness	NA
CVDR1HR	Recovery 1 heart rate (per min)	2003-2004	examination	physical fitness	NA
CVDR1SY	Recovery 1 systolic BP (mm Hg)	2003-2004	examination	physical fitness	NA
CVDR1DI	Recovery 1 diastolic BP (mm Hg)	2003-2004	examination	physical fitness	NA
CVDR2HR	Recovery 2 heart rate (per min)	2003-2004	examination	physical fitness	NA
CVDR2SY	Recovery 2 systolic BP (mm Hg)	2003-2004	examination	physical fitness	NA
CVDR2DI	Recovery 2 diastolic BP (mm Hg)	2003-2004	examination	physical fitness	NA
physical_activity	Physical Activity (MET-based rank)	1999-2000	questionnaire	physical fitness	NA
physical_activity	Physical Activity (MET-based rank)	2001-2002	questionnaire	physical fitness	NA
physical_activity	Physical Activity (MET-based rank)	2003-2004	questionnaire	physical fitness	NA
physical_activity	Physical Activity (MET-based rank)	2005-2006	questionnaire	physical fitness	NA
LBXPFDE	Perfluorodecanoic acid	1999-2000	laboratory	polyflourochemicals	NA
LBXEPAH	2-(N-ethyl-PFOSA) acetate	1999-2000	laboratory	polyflourochemicals	NA
LBXPFDO	Perfluorododecanoic acid	1999-2000	laboratory	polyflourochemicals	NA
LBXPFHP	Perfluoroheptanoic acid	1999-2000	laboratory	polyflourochemicals	NA
LBXPFHS	Perfluorohexane sulfonic acid	1999-2000	laboratory	polyflourochemicals	NA
LBXPFNA	Perfluorononanoic acid	1999-2000	laboratory	polyflourochemicals	NA
LBXPFOA	Perfluorooctanoic acid	1999-2000	laboratory	polyflourochemicals	NA
LBXPFOS	Perfluorooctane sulfonic acid	1999-2000	laboratory	polyflourochemicals	NA
LBXPFSA	Perfluorooctane sulfonamide	1999-2000	laboratory	polyflourochemicals	NA
LBXPFUA	Perfluoroundecanoic acid	1999-2000	laboratory	polyflourochemicals	NA
LBXMPAH	2-(N-methyl-PFOSA) acetate	1999-2000	laboratory	polyflourochemicals	NA
BPXPLS	60 sec. pulse (30 sec. pulse * 2):	2003-2004	examination	blood pressure	NA
BPXPLS	60 sec. pulse (30 sec. pulse * 2):	2005-2006	examination	blood pressure	NA
BPXPLS	60 sec. pulse (30 sec. pulse * 2):	2001-2002	examination	blood pressure	NA
BPXPLS	60 sec. pulse (30 sec. pulse * 2):	1999-2000	examination	blood pressure	NA
supplement_count	total supplement count	1999-2000	questionnaire	supplement use	NA
supplement_count	total supplement count	2001-2002	questionnaire	supplement use	NA
supplement_count	total supplement count	2003-2004	questionnaire	supplement use	NA
supplement_count	total supplement count	2005-2006	questionnaire	supplement use	NA
DSDCOUNT	DSDCOUNT	1999-2000	questionnaire	supplement use	NA
VITAMIN_B_6_mg	VITAMIN_B_6_mg	1999-2000	questionnaire	supplement use	NA
VITAMIN_B_12_mcg	VITAMIN_B_12_mcg	1999-2000	questionnaire	supplement use	NA
VITAMIN_C_mg	VITAMIN_C_mg	1999-2000	questionnaire	supplement use	NA
VITAMIN_A_IU	VITAMIN_A_IU	1999-2000	questionnaire	supplement use	NA
FOLIC_ACID_mcg	FOLIC_ACID_mcg	1999-2000	questionnaire	supplement use	NA
OMEGA_6_FATTY_ACIDS_NA	OMEGA_6_FATTY_ACIDS_NA	1999-2000	questionnaire	supplement use	NA
OMEGA_3_FATTY_ACIDS_mg	OMEGA_3_FATTY_ACIDS_mg	1999-2000	questionnaire	supplement use	NA
OMEGA_9_FATTY_ACIDS_NA	OMEGA_9_FATTY_ACIDS_NA	1999-2000	questionnaire	supplement use	NA
OTHER_FATTY_ACIDS_NA	OTHER_FATTY_ACIDS_NA	1999-2000	questionnaire	supplement use	NA
OTHER_OMEGA_3_FATTY_ACIDS_NA	OTHER_OMEGA_3_FATTY_ACIDS_NA	1999-2000	questionnaire	supplement use	NA
ALANINE_mg	ALANINE_mg	1999-2000	questionnaire	supplement use	NA
ALCOHOL_PERCENT	ALCOHOL_PERCENT	1999-2000	questionnaire	supplement use	NA
ARGININE_mg	ARGININE_mg	1999-2000	questionnaire	supplement use	NA
L_ASPARTIC_ACID_mg	L_ASPARTIC_ACID_mg	1999-2000	questionnaire	supplement use	NA
L_ASPARTIC_ACID_gm	L_ASPARTIC_ACID_gm	1999-2000	questionnaire	supplement use	NA
BETA_CAROTENE_IU	BETA_CAROTENE_IU	1999-2000	questionnaire	supplement use	NA
BETA_CAROTENE_mg	BETA_CAROTENE_mg	1999-2000	questionnaire	supplement use	NA
BETA_CAROTENE_mcg	BETA_CAROTENE_mcg	1999-2000	questionnaire	supplement use	NA
CAFFEINE_mg	CAFFEINE_mg	1999-2000	questionnaire	supplement use	NA
CALCIUM_mg	CALCIUM_mg	1999-2000	questionnaire	supplement use	NA
CALCIUM_PPM	CALCIUM_PPM	1999-2000	questionnaire	supplement use	NA
CHOLESTEROL_mg	CHOLESTEROL_mg	1999-2000	questionnaire	supplement use	NA
CYSTINE_mg	CYSTINE_mg	1999-2000	questionnaire	supplement use	NA
L_GLUTAMINE_mg	L_GLUTAMINE_mg	1999-2000	questionnaire	supplement use	NA
L_GLUTAMINE_gm	L_GLUTAMINE_gm	1999-2000	questionnaire	supplement use	NA
GLYCINE_mg	GLYCINE_mg	1999-2000	questionnaire	supplement use	NA
GLYCINE_gm	GLYCINE_gm	1999-2000	questionnaire	supplement use	NA
HISTIDINE_mg	HISTIDINE_mg	1999-2000	questionnaire	supplement use	NA
IRON_mg	IRON_mg	1999-2000	questionnaire	supplement use	NA
ISOLEUCINE_mg	ISOLEUCINE_mg	1999-2000	questionnaire	supplement use	NA
LEUCINE_mg	LEUCINE_mg	1999-2000	questionnaire	supplement use	NA
LYSINE_mg	LYSINE_mg	1999-2000	questionnaire	supplement use	NA
MAGNESIUM_mg	MAGNESIUM_mg	1999-2000	questionnaire	supplement use	NA
MAGNESIUM_PPM	MAGNESIUM_PPM	1999-2000	questionnaire	supplement use	NA
METHIONINE_mg	METHIONINE_mg	1999-2000	questionnaire	supplement use	NA
PHENYLALANINE_mg	PHENYLALANINE_mg	1999-2000	questionnaire	supplement use	NA
PHOSPHORUS_mg	PHOSPHORUS_mg	1999-2000	questionnaire	supplement use	NA
POTASSIUM_mg	POTASSIUM_mg	1999-2000	questionnaire	supplement use	NA
PROLINE_mg	PROLINE_mg	1999-2000	questionnaire	supplement use	NA
PROTEIN_gm	PROTEIN_gm	1999-2000	questionnaire	supplement use	NA
RIBOFLAVIN_mg	RIBOFLAVIN_mg	1999-2000	questionnaire	supplement use	NA
SELENIUM_mcg	SELENIUM_mcg	1999-2000	questionnaire	supplement use	NA
SERINE_mg	SERINE_mg	1999-2000	questionnaire	supplement use	NA
SODIUM_mg	SODIUM_mg	1999-2000	questionnaire	supplement use	NA
THIAMIN_mg	THIAMIN_mg	1999-2000	questionnaire	supplement use	NA
THREONINE_mg	THREONINE_mg	1999-2000	questionnaire	supplement use	NA
ALPHA_TOCOPHEROL_NA	ALPHA_TOCOPHEROL_NA	1999-2000	questionnaire	supplement use	NA
TOTAL_CARBOHYDRATE_gm	TOTAL_CARBOHYDRATE_gm	1999-2000	questionnaire	supplement use	NA
TRYPTOPHAN_mg	TRYPTOPHAN_mg	1999-2000	questionnaire	supplement use	NA
TYROSINE_mg	TYROSINE_mg	1999-2000	questionnaire	supplement use	NA
VALINE_mg	VALINE_mg	1999-2000	questionnaire	supplement use	NA
DSDCOUNT	DSDCOUNT	2001-2002	questionnaire	supplement use	NA
VITAMIN_B_6_mg	VITAMIN_B_6_mg	2001-2002	questionnaire	supplement use	NA
VITAMIN_B_6_Unknown	VITAMIN_B_6_Unknown	2001-2002	questionnaire	supplement use	NA
VITAMIN_B_12_mcg	VITAMIN_B_12_mcg	2001-2002	questionnaire	supplement use	NA
VITAMIN_B_12_Unknown	VITAMIN_B_12_Unknown	2001-2002	questionnaire	supplement use	NA
VITAMIN_C_mg	VITAMIN_C_mg	2001-2002	questionnaire	supplement use	NA
VITAMIN_C_Unknown	VITAMIN_C_Unknown	2001-2002	questionnaire	supplement use	NA
VITAMIN_A_IU	VITAMIN_A_IU	2001-2002	questionnaire	supplement use	NA
VITAMIN_A_mg	VITAMIN_A_mg	2001-2002	questionnaire	supplement use	NA
VITAMIN_A_Unknown	VITAMIN_A_Unknown	2001-2002	questionnaire	supplement use	NA
VITAMIN_A_mcg	VITAMIN_A_mcg	2001-2002	questionnaire	supplement use	NA
FOLIC_ACID_mcg	FOLIC_ACID_mcg	2001-2002	questionnaire	supplement use	NA
FOLIC_ACID_Unknown	FOLIC_ACID_Unknown	2001-2002	questionnaire	supplement use	NA
OMEGA_6_FATTY_ACIDS_NA	OMEGA_6_FATTY_ACIDS_NA	2001-2002	questionnaire	supplement use	NA
OMEGA_3_FATTY_ACIDS_mg	OMEGA_3_FATTY_ACIDS_mg	2001-2002	questionnaire	supplement use	NA
OMEGA_3_FATTY_ACIDS_Unknown	OMEGA_3_FATTY_ACIDS_Unknown	2001-2002	questionnaire	supplement use	NA
OMEGA_9_FATTY_ACIDS_NA	OMEGA_9_FATTY_ACIDS_NA	2001-2002	questionnaire	supplement use	NA
OTHER_FATTY_ACIDS_mg	OTHER_FATTY_ACIDS_mg	2001-2002	questionnaire	supplement use	NA
OTHER_OMEGA_3_FATTY_ACIDS_NA	OTHER_OMEGA_3_FATTY_ACIDS_NA	2001-2002	questionnaire	supplement use	NA
ALANINE_mg	ALANINE_mg	2001-2002	questionnaire	supplement use	NA
ALCOHOL_PERCENT	ALCOHOL_PERCENT	2001-2002	questionnaire	supplement use	NA
ARGININE_gm	ARGININE_gm	2001-2002	questionnaire	supplement use	NA
ARGININE_mg	ARGININE_mg	2001-2002	questionnaire	supplement use	NA
BETA_CAROTENE_mg	BETA_CAROTENE_mg	2001-2002	questionnaire	supplement use	NA
BETA_CAROTENE_IU	BETA_CAROTENE_IU	2001-2002	questionnaire	supplement use	NA
BETA_CAROTENE_Unknown	BETA_CAROTENE_Unknown	2001-2002	questionnaire	supplement use	NA
CAFFEINE_mg	CAFFEINE_mg	2001-2002	questionnaire	supplement use	NA
CALCIUM_mg	CALCIUM_mg	2001-2002	questionnaire	supplement use	NA
CALCIUM_Trace	CALCIUM_Trace	2001-2002	questionnaire	supplement use	NA
CALCIUM_Unknown	CALCIUM_Unknown	2001-2002	questionnaire	supplement use	NA
CHOLESTEROL_mg	CHOLESTEROL_mg	2001-2002	questionnaire	supplement use	NA
COPPER_mg	COPPER_mg	2001-2002	questionnaire	supplement use	NA
COPPER_Trace	COPPER_Trace	2001-2002	questionnaire	supplement use	NA
COPPER_Unknown	COPPER_Unknown	2001-2002	questionnaire	supplement use	NA
CYSTINE_mg	CYSTINE_mg	2001-2002	questionnaire	supplement use	NA
L_GLUTAMINE_mg	L_GLUTAMINE_mg	2001-2002	questionnaire	supplement use	NA
L_GLUTAMINE_gm	L_GLUTAMINE_gm	2001-2002	questionnaire	supplement use	NA
GLYCINE_mg	GLYCINE_mg	2001-2002	questionnaire	supplement use	NA
HISTIDINE_mg	HISTIDINE_mg	2001-2002	questionnaire	supplement use	NA
IRON_mg	IRON_mg	2001-2002	questionnaire	supplement use	NA
IRON_Trace	IRON_Trace	2001-2002	questionnaire	supplement use	NA
IRON_Unknown	IRON_Unknown	2001-2002	questionnaire	supplement use	NA
ISOLEUCINE_mg	ISOLEUCINE_mg	2001-2002	questionnaire	supplement use	NA
LEUCINE_mg	LEUCINE_mg	2001-2002	questionnaire	supplement use	NA
LYSINE_mg	LYSINE_mg	2001-2002	questionnaire	supplement use	NA
MAGNESIUM_mg	MAGNESIUM_mg	2001-2002	questionnaire	supplement use	NA
MAGNESIUM_Unknown	MAGNESIUM_Unknown	2001-2002	questionnaire	supplement use	NA
METHIONINE_mg	METHIONINE_mg	2001-2002	questionnaire	supplement use	NA
PHENYLALANINE_mg	PHENYLALANINE_mg	2001-2002	questionnaire	supplement use	NA
PHOSPHORUS_mg	PHOSPHORUS_mg	2001-2002	questionnaire	supplement use	NA
PHOSPHORUS_Trace	PHOSPHORUS_Trace	2001-2002	questionnaire	supplement use	NA
PHOSPHORUS_Unknown	PHOSPHORUS_Unknown	2001-2002	questionnaire	supplement use	NA
POTASSIUM_mg	POTASSIUM_mg	2001-2002	questionnaire	supplement use	NA
PROLINE_mg	PROLINE_mg	2001-2002	questionnaire	supplement use	NA
PROTEIN_gm	PROTEIN_gm	2001-2002	questionnaire	supplement use	NA
RIBOFLAVIN_mg	RIBOFLAVIN_mg	2001-2002	questionnaire	supplement use	NA
RIBOFLAVIN_Unknown	RIBOFLAVIN_Unknown	2001-2002	questionnaire	supplement use	NA
SELENIUM_mcg	SELENIUM_mcg	2001-2002	questionnaire	supplement use	NA
SELENIUM_Trace	SELENIUM_Trace	2001-2002	questionnaire	supplement use	NA
SELENIUM_Unknown	SELENIUM_Unknown	2001-2002	questionnaire	supplement use	NA
SERINE_mg	SERINE_mg	2001-2002	questionnaire	supplement use	NA
SODIUM_mg	SODIUM_mg	2001-2002	questionnaire	supplement use	NA
THIAMIN_mg	THIAMIN_mg	2001-2002	questionnaire	supplement use	NA
THIAMIN_Unknown	THIAMIN_Unknown	2001-2002	questionnaire	supplement use	NA
THREONINE_mg	THREONINE_mg	2001-2002	questionnaire	supplement use	NA
ALPHA_TOCOPHEROL_IU	ALPHA_TOCOPHEROL_IU	2001-2002	questionnaire	supplement use	NA
TOTAL_CARBOHYDRATE_gm	TOTAL_CARBOHYDRATE_gm	2001-2002	questionnaire	supplement use	NA
TRYPTOPHAN_mg	TRYPTOPHAN_mg	2001-2002	questionnaire	supplement use	NA
TYROSINE_mg	TYROSINE_mg	2001-2002	questionnaire	supplement use	NA
VALINE_mg	VALINE_mg	2001-2002	questionnaire	supplement use	NA
DSDCOUNT	DSDCOUNT	2003-2004	questionnaire	supplement use	NA
VITAMIN_B_6_mg	VITAMIN_B_6_mg	2003-2004	questionnaire	supplement use	NA
VITAMIN_B_6_Unknown	VITAMIN_B_6_Unknown	2003-2004	questionnaire	supplement use	NA
VITAMIN_B_12_mcg	VITAMIN_B_12_mcg	2003-2004	questionnaire	supplement use	NA
VITAMIN_B_12_Unknown	VITAMIN_B_12_Unknown	2003-2004	questionnaire	supplement use	NA
VITAMIN_C_Unknown	VITAMIN_C_Unknown	2003-2004	questionnaire	supplement use	NA
VITAMIN_A_IU	VITAMIN_A_IU	2003-2004	questionnaire	supplement use	NA
VITAMIN_A_Unknown	VITAMIN_A_Unknown	2003-2004	questionnaire	supplement use	NA
VITAMIN_A_mcg	VITAMIN_A_mcg	2003-2004	questionnaire	supplement use	NA
FOLIC_ACID_mcg	FOLIC_ACID_mcg	2003-2004	questionnaire	supplement use	NA
FOLIC_ACID_Unknown	FOLIC_ACID_Unknown	2003-2004	questionnaire	supplement use	NA
OMEGA_6_FATTY_ACIDS_mg	OMEGA_6_FATTY_ACIDS_mg	2003-2004	questionnaire	supplement use	NA
OMEGA_3_FATTY_ACIDS_mg	OMEGA_3_FATTY_ACIDS_mg	2003-2004	questionnaire	supplement use	NA
OMEGA_9_FATTY_ACIDS_mg	OMEGA_9_FATTY_ACIDS_mg	2003-2004	questionnaire	supplement use	NA
OTHER_FATTY_ACIDS_mg	OTHER_FATTY_ACIDS_mg	2003-2004	questionnaire	supplement use	NA
OTHER_OMEGA_3_FATTY_ACIDS_NA	OTHER_OMEGA_3_FATTY_ACIDS_NA	2003-2004	questionnaire	supplement use	NA
ALANINE_mg	ALANINE_mg	2003-2004	questionnaire	supplement use	NA
ALCOHOL_PERCENT	ALCOHOL_PERCENT	2003-2004	questionnaire	supplement use	NA
ARGININE_mg	ARGININE_mg	2003-2004	questionnaire	supplement use	NA
L_ASPARTIC_ACID_mg	L_ASPARTIC_ACID_mg	2003-2004	questionnaire	supplement use	NA
BETA_CAROTENE_IU	BETA_CAROTENE_IU	2003-2004	questionnaire	supplement use	NA
BETA_CAROTENE_mg	BETA_CAROTENE_mg	2003-2004	questionnaire	supplement use	NA
BETA_CAROTENE__	BETA_CAROTENE__	2003-2004	questionnaire	supplement use	NA
BETA_CAROTENE_mcg	BETA_CAROTENE_mcg	2003-2004	questionnaire	supplement use	NA
CAFFEINE_mg	CAFFEINE_mg	2003-2004	questionnaire	supplement use	NA
CALCIUM_mg	CALCIUM_mg	2003-2004	questionnaire	supplement use	NA
CALCIUM_Unknown	CALCIUM_Unknown	2003-2004	questionnaire	supplement use	NA
CHOLESTEROL_mg	CHOLESTEROL_mg	2003-2004	questionnaire	supplement use	NA
COPPER_mg	COPPER_mg	2003-2004	questionnaire	supplement use	NA
COPPER_Unknown	COPPER_Unknown	2003-2004	questionnaire	supplement use	NA
CYSTINE_mg	CYSTINE_mg	2003-2004	questionnaire	supplement use	NA
L_GLUTAMINE_mg	L_GLUTAMINE_mg	2003-2004	questionnaire	supplement use	NA
L_GLUTAMINE_gm	L_GLUTAMINE_gm	2003-2004	questionnaire	supplement use	NA
GLYCINE_mg	GLYCINE_mg	2003-2004	questionnaire	supplement use	NA
GLYCINE_gm	GLYCINE_gm	2003-2004	questionnaire	supplement use	NA
HISTIDINE_mg	HISTIDINE_mg	2003-2004	questionnaire	supplement use	NA
IRON_mg	IRON_mg	2003-2004	questionnaire	supplement use	NA
IRON_Unknown	IRON_Unknown	2003-2004	questionnaire	supplement use	NA
ISOLEUCINE_mg	ISOLEUCINE_mg	2003-2004	questionnaire	supplement use	NA
LEUCINE_mg	LEUCINE_mg	2003-2004	questionnaire	supplement use	NA
LYSINE_mg	LYSINE_mg	2003-2004	questionnaire	supplement use	NA
MAGNESIUM_mg	MAGNESIUM_mg	2003-2004	questionnaire	supplement use	NA
MAGNESIUM_Unknown	MAGNESIUM_Unknown	2003-2004	questionnaire	supplement use	NA
METHIONINE_mg	METHIONINE_mg	2003-2004	questionnaire	supplement use	NA
PHENYLALANINE_mg	PHENYLALANINE_mg	2003-2004	questionnaire	supplement use	NA
PHOSPHORUS_mg	PHOSPHORUS_mg	2003-2004	questionnaire	supplement use	NA
PHOSPHORUS_Unknown	PHOSPHORUS_Unknown	2003-2004	questionnaire	supplement use	NA
POTASSIUM_mg	POTASSIUM_mg	2003-2004	questionnaire	supplement use	NA
PROLINE_mg	PROLINE_mg	2003-2004	questionnaire	supplement use	NA
PROTEIN_gm	PROTEIN_gm	2003-2004	questionnaire	supplement use	NA
RIBOFLAVIN_mg	RIBOFLAVIN_mg	2003-2004	questionnaire	supplement use	NA
RIBOFLAVIN_Unknown	RIBOFLAVIN_Unknown	2003-2004	questionnaire	supplement use	NA
SELENIUM_mcg	SELENIUM_mcg	2003-2004	questionnaire	supplement use	NA
SELENIUM_Unknown	SELENIUM_Unknown	2003-2004	questionnaire	supplement use	NA
SERINE_mg	SERINE_mg	2003-2004	questionnaire	supplement use	NA
SODIUM_mg	SODIUM_mg	2003-2004	questionnaire	supplement use	NA
SODIUM_Trace	SODIUM_Trace	2003-2004	questionnaire	supplement use	NA
THIAMIN_Unknown	THIAMIN_Unknown	2003-2004	questionnaire	supplement use	NA
THREONINE_mg	THREONINE_mg	2003-2004	questionnaire	supplement use	NA
ALPHA_TOCOPHEROL_mg	ALPHA_TOCOPHEROL_mg	2003-2004	questionnaire	supplement use	NA
TOTAL_CARBOHYDRATE_gm	TOTAL_CARBOHYDRATE_gm	2003-2004	questionnaire	supplement use	NA
TRYPTOPHAN_mg	TRYPTOPHAN_mg	2003-2004	questionnaire	supplement use	NA
TYROSINE_mg	TYROSINE_mg	2003-2004	questionnaire	supplement use	NA
VALINE_mg	VALINE_mg	2003-2004	questionnaire	supplement use	NA
DSDCOUNT	DSDCOUNT	2005-2006	questionnaire	supplement use	NA
VITAMIN_B_6_mg	VITAMIN_B_6_mg	2005-2006	questionnaire	supplement use	NA
VITAMIN_B_12_mcg	VITAMIN_B_12_mcg	2005-2006	questionnaire	supplement use	NA
VITAMIN_C_mg	VITAMIN_C_mg	2005-2006	questionnaire	supplement use	NA
VITAMIN_A_IU	VITAMIN_A_IU	2005-2006	questionnaire	supplement use	NA
VITAMIN_A_mcg	VITAMIN_A_mcg	2005-2006	questionnaire	supplement use	NA
FOLIC_ACID_mcg	FOLIC_ACID_mcg	2005-2006	questionnaire	supplement use	NA
OMEGA_6_FATTY_ACIDS_mg	OMEGA_6_FATTY_ACIDS_mg	2005-2006	questionnaire	supplement use	NA
OMEGA_3_FATTY_ACIDS_mg	OMEGA_3_FATTY_ACIDS_mg	2005-2006	questionnaire	supplement use	NA
OMEGA_9_FATTY_ACIDS_mg	OMEGA_9_FATTY_ACIDS_mg	2005-2006	questionnaire	supplement use	NA
OTHER_FATTY_ACIDS_mg	OTHER_FATTY_ACIDS_mg	2005-2006	questionnaire	supplement use	NA
OTHER_OMEGA_3_FATTY_ACIDS_NA	OTHER_OMEGA_3_FATTY_ACIDS_NA	2005-2006	questionnaire	supplement use	NA
ALANINE_mg	ALANINE_mg	2005-2006	questionnaire	supplement use	NA
ALCOHOL_PERCENT	ALCOHOL_PERCENT	2005-2006	questionnaire	supplement use	NA
ARGININE_mg	ARGININE_mg	2005-2006	questionnaire	supplement use	NA
L_ASPARTIC_ACID_mg	L_ASPARTIC_ACID_mg	2005-2006	questionnaire	supplement use	NA
L_ASPARTIC_ACID_gm	L_ASPARTIC_ACID_gm	2005-2006	questionnaire	supplement use	NA
BETA_CAROTENE_IU	BETA_CAROTENE_IU	2005-2006	questionnaire	supplement use	NA
BETA_CAROTENE_mg	BETA_CAROTENE_mg	2005-2006	questionnaire	supplement use	NA
BETA_CAROTENE_mcg	BETA_CAROTENE_mcg	2005-2006	questionnaire	supplement use	NA
CAFFEINE_mg	CAFFEINE_mg	2005-2006	questionnaire	supplement use	NA
CALCIUM_mg	CALCIUM_mg	2005-2006	questionnaire	supplement use	NA
CHOLESTEROL_mg	CHOLESTEROL_mg	2005-2006	questionnaire	supplement use	NA
COPPER_mg	COPPER_mg	2005-2006	questionnaire	supplement use	NA
CYSTINE_mg	CYSTINE_mg	2005-2006	questionnaire	supplement use	NA
L_GLUTAMINE_mg	L_GLUTAMINE_mg	2005-2006	questionnaire	supplement use	NA
L_GLUTAMINE_gm	L_GLUTAMINE_gm	2005-2006	questionnaire	supplement use	NA
GLYCINE_mg	GLYCINE_mg	2005-2006	questionnaire	supplement use	NA
HISTIDINE_mg	HISTIDINE_mg	2005-2006	questionnaire	supplement use	NA
IRON_mg	IRON_mg	2005-2006	questionnaire	supplement use	NA
ISOLEUCINE_mg	ISOLEUCINE_mg	2005-2006	questionnaire	supplement use	NA
LEUCINE_mg	LEUCINE_mg	2005-2006	questionnaire	supplement use	NA
LYSINE_mg	LYSINE_mg	2005-2006	questionnaire	supplement use	NA
MAGNESIUM_mg	MAGNESIUM_mg	2005-2006	questionnaire	supplement use	NA
METHIONINE_mg	METHIONINE_mg	2005-2006	questionnaire	supplement use	NA
PHENYLALANINE_mg	PHENYLALANINE_mg	2005-2006	questionnaire	supplement use	NA
PHOSPHORUS_mg	PHOSPHORUS_mg	2005-2006	questionnaire	supplement use	NA
POTASSIUM_mg	POTASSIUM_mg	2005-2006	questionnaire	supplement use	NA
PROLINE_mg	PROLINE_mg	2005-2006	questionnaire	supplement use	NA
PROTEIN_gm	PROTEIN_gm	2005-2006	questionnaire	supplement use	NA
RIBOFLAVIN_mg	RIBOFLAVIN_mg	2005-2006	questionnaire	supplement use	NA
SELENIUM_mcg	SELENIUM_mcg	2005-2006	questionnaire	supplement use	NA
SERINE_mg	SERINE_mg	2005-2006	questionnaire	supplement use	NA
SODIUM_mg	SODIUM_mg	2005-2006	questionnaire	supplement use	NA
THIAMIN_mg	THIAMIN_mg	2005-2006	questionnaire	supplement use	NA
ALPHA_TOCOPHEROL_mg	ALPHA_TOCOPHEROL_mg	2005-2006	questionnaire	supplement use	NA
TOTAL_CARBOHYDRATE_gm	TOTAL_CARBOHYDRATE_gm	2005-2006	questionnaire	supplement use	NA
TRYPTOPHAN_mg	TRYPTOPHAN_mg	2005-2006	questionnaire	supplement use	NA
TYROSINE_mg	TYROSINE_mg	2005-2006	questionnaire	supplement use	NA
VALINE_mg	VALINE_mg	2005-2006	questionnaire	supplement use	NA
DIETARY_FIBER_mg	DIETARY_FIBER_mg	1999-2000	questionnaire	supplement use	NA
DIETARY_FIBER_mg	DIETARY_FIBER_mg	2001-2002	questionnaire	supplement use	NA
DIETARY_FIBER_mg	DIETARY_FIBER_mg	2003-2004	questionnaire	supplement use	NA
DIETARY_FIBER_mg	DIETARY_FIBER_mg	2005-2006	questionnaire	supplement use	NA
URX24D	2,4-D (ug/L)	2003-2004	laboratory	pesticides	NA
URX25T	2,4,5 Trichlorophenoxyacetic acid (ug/L)	2003-2004	laboratory	pesticides	NA
URXBSM	Bensulfuron methyl (ug/L)	2003-2004	laboratory	pesticides	NA
URXCHS	Chlorsulfuron (ug/L)	2003-2004	laboratory	pesticides	NA
URXEMM	Ethametsulfuron methyl (ug/L)	2003-2004	laboratory	pesticides	NA
URXFRM	Foramsulfuron (ug/L)	2003-2004	laboratory	pesticides	NA
URXHLS	Halosulfuron (ug/L)	2003-2004	laboratory	pesticides	NA
URXMSM	Mesosulfuron methyl (ug/L)	2003-2004	laboratory	pesticides	NA
URXMTM	Metsulfuron methyl (ug/L)	2003-2004	laboratory	pesticides	NA
URXNOS	Nicosulfuron (ug/L)	2003-2004	laboratory	pesticides	NA
URXOXS	Oxasulfuron (ug/L)	2003-2004	laboratory	pesticides	NA
URXPIM	Primisulfuron methyl (ug/L)	2003-2004	laboratory	pesticides	NA
URXPRO	Prosulfuron (ug/L)	2003-2004	laboratory	pesticides	NA
URXRIM	Rimsulfuron (ug/L)	2003-2004	laboratory	pesticides	NA
URXSMM	Sulfometuron methyl (ug/L)	2003-2004	laboratory	pesticides	NA
URXSSF	Sulfosulfuron (ug/L)	2003-2004	laboratory	pesticides	NA
URXTHF	Thifensulfuron methyl (ug/L)	2003-2004	laboratory	pesticides	NA
URXTRA	Triasulfuron (ug/L)	2003-2004	laboratory	pesticides	NA
URXTRN	Triflusulfuron methyl (ug/L)	2003-2004	laboratory	pesticides	NA
URXUCR	Creatinine, urine (mg/dL)	2003-2004	laboratory	biochemistry	NA
cigarette_smoking	Current Cigarette Smoker?	1999-2000	questionnaire	smoking behavior	NA
cigarette_smoking	Current Cigarette Smoker?	2001-2002	questionnaire	smoking behavior	NA
cigarette_smoking	Current Cigarette Smoker?	2003-2004	questionnaire	smoking behavior	NA
cigarette_smoking	Current Cigarette Smoker?	2005-2006	questionnaire	smoking behavior	NA
URXCNP	Mono(carboxynonyl) phthalate (ng/mL)	2005-2006	laboratory	phthalates	NA
URXCOP	Mono(carboxyoctyl) phthalate(ng/mL)	2005-2006	laboratory	phthalates	NA
URXMBP	Mono-n-butyl phthalate	2005-2006	laboratory	phthalates	NA
URXMCP	Mono-cyclohexyl phthalate	2005-2006	laboratory	phthalates	NA
URXMEP	Mono-ethyl phthalate	2005-2006	laboratory	phthalates	NA
URXMHP	Mono-(2-ethyl)-hexyl phthalate	2005-2006	laboratory	phthalates	NA
URXMNP	Mono-isononyl phthalate	2005-2006	laboratory	phthalates	NA
URXMOP	Mono-n-octyl phthalate	2005-2006	laboratory	phthalates	NA
URXMZP	Mono-benzyl phthalate	2005-2006	laboratory	phthalates	NA
URXMNM	Mono-n-methyl phthalate	2005-2006	laboratory	phthalates	NA
URXMC1	Mono-(3-carboxypropyl) phthalate	2005-2006	laboratory	phthalates	NA
URXMHH	Mono-(2-ethyl-5-hydroxyhexyl) phthalate	2005-2006	laboratory	phthalates	NA
URXMOH	Mono-(2-ethyl-5-oxohexyl) phthalate	2005-2006	laboratory	phthalates	NA
URXMIB	Mono-isobutyl pthalate	2005-2006	laboratory	phthalates	NA
URXECP	Mono-2-ethyl-5-carboxypentyl phthalate	2005-2006	laboratory	phthalates	NA
URXUP8	Urinary perchlorate (ng/mL)	2005-2006	laboratory	perchlorate	NA
URXNO3	Urinary nitrate (ng/mL)	2005-2006	laboratory	perchlorate	NA
URXSCN	Urinary thiocyanate (ng/mL)	2005-2006	laboratory	perchlorate	NA
URXDAZ	Daidzein (ng/mL)	2005-2006	laboratory	phytoestrogens	NA
URXEQU	Equol (ng/mL)	2005-2006	laboratory	phytoestrogens	NA
URXETD	Enterodiol (ng/mL)	2005-2006	laboratory	phytoestrogens	NA
URXETL	Enterolactone (ng/mL)	2005-2006	laboratory	phytoestrogens	NA
URXGNS	Genistein (ng/mL)	2005-2006	laboratory	phytoestrogens	NA
URXBSM	Bensulfuron methyl (ug/L)	2005-2006	laboratory	pesticides	NA
URXCHS	Chlorsulfuron (ug/L)	2005-2006	laboratory	pesticides	NA
URXEMM	Ethametsulfuron methyl (ug/L)	2005-2006	laboratory	pesticides	NA
URXFRM	Foramsulfuron (ug/L)	2005-2006	laboratory	pesticides	NA
URXHLS	Halosulfuron (ug/L)	2005-2006	laboratory	pesticides	NA
URXMSM	Mesosulfuron methyl (ug/L)	2005-2006	laboratory	pesticides	NA
URXMTM	Metsulfuron methyl (ug/L)	2005-2006	laboratory	pesticides	NA
URXNOS	Nicosulfuron (ug/L)	2005-2006	laboratory	pesticides	NA
URXOXS	Oxasulfuron (ug/L)	2005-2006	laboratory	pesticides	NA
URXPIM	Primisulfuron methyl (ug/L)	2005-2006	laboratory	pesticides	NA
URXPRO	Prosulfuron (ug/L)	2005-2006	laboratory	pesticides	NA
URXRIM	Rimsulfuron (ug/L)	2005-2006	laboratory	pesticides	NA
URXSMM	Sulfometuron methyl (ug/L)	2005-2006	laboratory	pesticides	NA
URXSSF	Sulfosulfuron (ug/L)	2005-2006	laboratory	pesticides	NA
URXTHF	Thifensulfuron methyl (ug/L)	2005-2006	laboratory	pesticides	NA
URXTRA	Triasulfuron (ug/L)	2005-2006	laboratory	pesticides	NA
URXTRN	Triflusulfuron methyl (ug/L)	2005-2006	laboratory	pesticides	NA
LBXV1A	Blood 1,1-Dichloroethane (ng/mL)	2005-2006	laboratory	volatile compounds	NA
LBXV1D	Blood 1,2-Dichlorobenzene (ng/mL)	2005-2006	laboratory	volatile compounds	NA
LBXV1E	Blood 1,1-Dichloroethene (ng/mL)	2005-2006	laboratory	volatile compounds	NA
LBXV2A	Blood 1,2-Dichloroethane (ng/mL)	2005-2006	laboratory	volatile compounds	NA
LBXV2C	Blood cis-1,2-Dichloroethene (ng/mL)	2005-2006	laboratory	volatile compounds	NA
LBXV2E	Blood 1,1,2-Trichloroethene (ng/mL)	2005-2006	laboratory	volatile compounds	NA
LBXV2P	Blood 1,2-Dibromo-3-chloropropane(ng/mL)	2005-2006	laboratory	volatile compounds	NA
LBXV2T	Blood trans-1,2-Dichloroethene (ng/mL)	2005-2006	laboratory	volatile compounds	NA
LBXV3B	Blood 1,3-Dichlorobenzene (ng/mL)	2005-2006	laboratory	volatile compounds	NA
LBXV4E	Blood 1,1,2,2-Tetrachloroethane (ng/mL)	2005-2006	laboratory	volatile compounds	NA
LBXV4C	Blood Tetrachloroethene (ng/mL)	2005-2006	laboratory	volatile compounds	NA
LBXVBF	Blood Bromoform (pg/mL)	2005-2006	laboratory	volatile compounds	NA
LBXVBM	Blood Bromodichloromethane (pg/mL)	2005-2006	laboratory	volatile compounds	NA
LBXVBZ	Blood Benzene (ng/mL)	2005-2006	laboratory	volatile compounds	NA
LBXVCB	Blood Chlorobenzene (ng/mL)	2005-2006	laboratory	volatile compounds	NA
LBXVCF	Blood Chloroform (pg/mL)	2005-2006	laboratory	volatile compounds	NA
LBXVCM	Blood Dibromochloromethane (pg/mL)	2005-2006	laboratory	volatile compounds	NA
LBXVCT	Blood Carbon Tetrachloride (ng/mL)	2005-2006	laboratory	volatile compounds	NA
LBXVDB	Blood 1,4-Dichlorobenzene (ng/mL)	2005-2006	laboratory	volatile compounds	NA
LBXVDM	Blood Dibromomethane (ng/mL)	2005-2006	laboratory	volatile compounds	NA
LBXVDP	Blood 1,2-Dichloropropane (ng/mL)	2005-2006	laboratory	volatile compounds	NA
LBXVEB	Blood Ethylbenzene (ng/mL)	2005-2006	laboratory	volatile compounds	NA
LBXVHE	Blood Hexachloroethane (ng/mL)	2005-2006	laboratory	volatile compounds	NA
LBXVMC	Blood Methylene Chloride (ng/mL)	2005-2006	laboratory	volatile compounds	NA
LBXVME	Blood MTBE (pg/mL)	2005-2006	laboratory	volatile compounds	NA
LBXVOX	Blood o-Xylene (ng/mL)	2005-2006	laboratory	volatile compounds	NA
LBXVST	Blood Styrene (ng/mL)	2005-2006	laboratory	volatile compounds	NA
LBXVTC	Blood Trichloroethene (ng/mL)	2005-2006	laboratory	volatile compounds	NA
LBXVTE	Blood 1,1,1-Trichloroethane (ng/mL)	2005-2006	laboratory	volatile compounds	NA
LBXVTO	Blood Toluene (ng/mL)	2005-2006	laboratory	volatile compounds	NA
LBXVXY	Blood m-/p-Xylene (ng/mL)	2005-2006	laboratory	volatile compounds	NA
LBX2DF	Blood 2,5-Dimethylfuran (ng/mL)	2005-2006	laboratory	volatile compounds	NA
any_diabetes	Any Diabetes (FBG >= 126 mg/dL or self-report)	1999-2000	questionnaire	disease	NA
any_diabetes	Any Diabetes (FBG >= 126 mg/dL or self-report)	2001-2002	questionnaire	disease	NA
any_diabetes	Any Diabetes (FBG >= 126 mg/dL or self-report)	2003-2004	questionnaire	disease	NA
any_diabetes	Any Diabetes (FBG >= 126 mg/dL or self-report)	2005-2006	questionnaire	disease	NA
any_ht	Any Hypertension? (BP >= 139/90 or self report)	1999-2000	questionnaire	disease	NA
any_ht	Any Hypertension? (BP >= 139/90 or self report)	2001-2002	questionnaire	disease	NA
any_ht	Any Hypertension? (BP >= 139/90 or self report)	2003-2004	questionnaire	disease	NA
any_ht	Any Hypertension? (BP >= 139/90 or self report)	2005-2006	questionnaire	disease	NA
cad	Any CAD (self report)	1999-2000	questionnaire	disease	NA
cad	Any CAD (self report)	2001-2002	questionnaire	disease	NA
cad	Any CAD (self report)	2003-2004	questionnaire	disease	NA
cad	Any CAD (self report)	2005-2006	questionnaire	disease	NA
bladder_cancer_self_report	bladder_cancer_self_report	1999-2000	questionnaire	disease	NA
bladder_cancer_self_report	bladder_cancer_self_report	2001-2002	questionnaire	disease	NA
bladder_cancer_self_report	bladder_cancer_self_report	2003-2004	questionnaire	disease	NA
bladder_cancer_self_report	bladder_cancer_self_report	2005-2006	questionnaire	disease	NA
blood_cancer_self_report	blood_cancer_self_report	1999-2000	questionnaire	disease	NA
blood_cancer_self_report	blood_cancer_self_report	2001-2002	questionnaire	disease	NA
blood_cancer_self_report	blood_cancer_self_report	2003-2004	questionnaire	disease	NA
blood_cancer_self_report	blood_cancer_self_report	2005-2006	questionnaire	disease	NA
bone_cancer_self_report	bone_cancer_self_report	1999-2000	questionnaire	disease	NA
bone_cancer_self_report	bone_cancer_self_report	2001-2002	questionnaire	disease	NA
bone_cancer_self_report	bone_cancer_self_report	2003-2004	questionnaire	disease	NA
bone_cancer_self_report	bone_cancer_self_report	2005-2006	questionnaire	disease	NA
brain_cancer_self_report	brain_cancer_self_report	1999-2000	questionnaire	disease	NA
brain_cancer_self_report	brain_cancer_self_report	2001-2002	questionnaire	disease	NA
brain_cancer_self_report	brain_cancer_self_report	2003-2004	questionnaire	disease	NA
brain_cancer_self_report	brain_cancer_self_report	2005-2006	questionnaire	disease	NA
breast_cancer_self_report	breast_cancer_self_report	1999-2000	questionnaire	disease	NA
breast_cancer_self_report	breast_cancer_self_report	2001-2002	questionnaire	disease	NA
breast_cancer_self_report	breast_cancer_self_report	2003-2004	questionnaire	disease	NA
breast_cancer_self_report	breast_cancer_self_report	2005-2006	questionnaire	disease	NA
cervix_cacner_self_report	cervix_cacner_self_report	1999-2000	questionnaire	disease	NA
cervix_cacner_self_report	cervix_cacner_self_report	2001-2002	questionnaire	disease	NA
cervix_cacner_self_report	cervix_cacner_self_report	2003-2004	questionnaire	disease	NA
cervix_cacner_self_report	cervix_cacner_self_report	2005-2006	questionnaire	disease	NA
colon_cancer_self_report	colon_cancer_self_report	1999-2000	questionnaire	disease	NA
colon_cancer_self_report	colon_cancer_self_report	2001-2002	questionnaire	disease	NA
colon_cancer_self_report	colon_cancer_self_report	2003-2004	questionnaire	disease	NA
colon_cancer_self_report	colon_cancer_self_report	2005-2006	questionnaire	disease	NA
esophagus_cancer_self_report	esophagus_cancer_self_report	1999-2000	questionnaire	disease	NA
esophagus_cancer_self_report	esophagus_cancer_self_report	2001-2002	questionnaire	disease	NA
esophagus_cancer_self_report	esophagus_cancer_self_report	2005-2006	questionnaire	disease	NA
gallbladder_cancer_self_report	gallbladder_cancer_self_report	1999-2000	questionnaire	disease	NA
gallbladder_cancer_self_report	gallbladder_cancer_self_report	2001-2002	questionnaire	disease	NA
gallbladder_cancer_self_report	gallbladder_cancer_self_report	2003-2004	questionnaire	disease	NA
gallbladder_cancer_self_report	gallbladder_cancer_self_report	2005-2006	questionnaire	disease	NA
kidney_cancer_self_report	kidney_cancer_self_report	1999-2000	questionnaire	disease	NA
kidney_cancer_self_report	kidney_cancer_self_report	2001-2002	questionnaire	disease	NA
kidney_cancer_self_report	kidney_cancer_self_report	2003-2004	questionnaire	disease	NA
kidney_cancer_self_report	kidney_cancer_self_report	2005-2006	questionnaire	disease	NA
larynx_cancer_self_report	larynx_cancer_self_report	1999-2000	questionnaire	disease	NA
larynx_cancer_self_report	larynx_cancer_self_report	2001-2002	questionnaire	disease	NA
larynx_cancer_self_report	larynx_cancer_self_report	2003-2004	questionnaire	disease	NA
larynx_cancer_self_report	larynx_cancer_self_report	2005-2006	questionnaire	disease	NA
leukemia_self_report	leukemia_self_report	1999-2000	questionnaire	disease	NA
leukemia_self_report	leukemia_self_report	2001-2002	questionnaire	disease	NA
leukemia_self_report	leukemia_self_report	2003-2004	questionnaire	disease	NA
leukemia_self_report	leukemia_self_report	2005-2006	questionnaire	disease	NA
liver_cancer_self_report	liver_cancer_self_report	1999-2000	questionnaire	disease	NA
liver_cancer_self_report	liver_cancer_self_report	2001-2002	questionnaire	disease	NA
liver_cancer_self_report	liver_cancer_self_report	2003-2004	questionnaire	disease	NA
liver_cancer_self_report	liver_cancer_self_report	2005-2006	questionnaire	disease	NA
lung_cancer_self_report	lung_cancer_self_report	1999-2000	questionnaire	disease	NA
lung_cancer_self_report	lung_cancer_self_report	2001-2002	questionnaire	disease	NA
lung_cancer_self_report	lung_cancer_self_report	2003-2004	questionnaire	disease	NA
lung_cancer_self_report	lung_cancer_self_report	2005-2006	questionnaire	disease	NA
lymphoma_self_report	lymphoma_self_report	1999-2000	questionnaire	disease	NA
lymphoma_self_report	lymphoma_self_report	2001-2002	questionnaire	disease	NA
lymphoma_self_report	lymphoma_self_report	2003-2004	questionnaire	disease	NA
lymphoma_self_report	lymphoma_self_report	2005-2006	questionnaire	disease	NA
melanoma_self_report	melanoma_self_report	1999-2000	questionnaire	disease	NA
melanoma_self_report	melanoma_self_report	2001-2002	questionnaire	disease	NA
melanoma_self_report	melanoma_self_report	2003-2004	questionnaire	disease	NA
melanoma_self_report	melanoma_self_report	2005-2006	questionnaire	disease	NA
mouth_cancer_self_report	mouth_cancer_self_report	1999-2000	questionnaire	disease	NA
mouth_cancer_self_report	mouth_cancer_self_report	2001-2002	questionnaire	disease	NA
mouth_cancer_self_report	mouth_cancer_self_report	2003-2004	questionnaire	disease	NA
mouth_cancer_self_report	mouth_cancer_self_report	2005-2006	questionnaire	disease	NA
nervous_cancer_self_report	nervous_cancer_self_report	1999-2000	questionnaire	disease	NA
nervous_cancer_self_report	nervous_cancer_self_report	2001-2002	questionnaire	disease	NA
nervous_cancer_self_report	nervous_cancer_self_report	2003-2004	questionnaire	disease	NA
nervous_cancer_self_report	nervous_cancer_self_report	2005-2006	questionnaire	disease	NA
ovarian_cancer_self_report	ovarian_cancer_self_report	1999-2000	questionnaire	disease	NA
ovarian_cancer_self_report	ovarian_cancer_self_report	2001-2002	questionnaire	disease	NA
ovarian_cancer_self_report	ovarian_cancer_self_report	2003-2004	questionnaire	disease	NA
ovarian_cancer_self_report	ovarian_cancer_self_report	2005-2006	questionnaire	disease	NA
pancreatic_cancer_self_report	pancreatic_cancer_self_report	1999-2000	questionnaire	disease	NA
pancreatic_cancer_self_report	pancreatic_cancer_self_report	2001-2002	questionnaire	disease	NA
pancreatic_cancer_self_report	pancreatic_cancer_self_report	2003-2004	questionnaire	disease	NA
pancreatic_cancer_self_report	pancreatic_cancer_self_report	2005-2006	questionnaire	disease	NA
prostate_cancer_self_report	prostate_cancer_self_report	1999-2000	questionnaire	disease	NA
prostate_cancer_self_report	prostate_cancer_self_report	2001-2002	questionnaire	disease	NA
prostate_cancer_self_report	prostate_cancer_self_report	2003-2004	questionnaire	disease	NA
prostate_cancer_self_report	prostate_cancer_self_report	2005-2006	questionnaire	disease	NA
rectum_cancer_self_report	rectum_cancer_self_report	1999-2000	questionnaire	disease	NA
rectum_cancer_self_report	rectum_cancer_self_report	2001-2002	questionnaire	disease	NA
rectum_cancer_self_report	rectum_cancer_self_report	2003-2004	questionnaire	disease	NA
rectum_cancer_self_report	rectum_cancer_self_report	2005-2006	questionnaire	disease	NA
skin_cancer_self_report	skin_cancer_self_report	1999-2000	questionnaire	disease	NA
skin_cancer_self_report	skin_cancer_self_report	2001-2002	questionnaire	disease	NA
skin_cancer_self_report	skin_cancer_self_report	2003-2004	questionnaire	disease	NA
skin_cancer_self_report	skin_cancer_self_report	2005-2006	questionnaire	disease	NA
other_skin_cancer_self_report	other_skin_cancer_self_report	1999-2000	questionnaire	disease	NA
other_skin_cancer_self_report	other_skin_cancer_self_report	2001-2002	questionnaire	disease	NA
other_skin_cancer_self_report	other_skin_cancer_self_report	2003-2004	questionnaire	disease	NA
other_skin_cancer_self_report	other_skin_cancer_self_report	2005-2006	questionnaire	disease	NA
soft_cancer_self_report	soft_cancer_self_report	1999-2000	questionnaire	disease	NA
soft_cancer_self_report	soft_cancer_self_report	2001-2002	questionnaire	disease	NA
soft_cancer_self_report	soft_cancer_self_report	2003-2004	questionnaire	disease	NA
soft_cancer_self_report	soft_cancer_self_report	2005-2006	questionnaire	disease	NA
stomach_cancer_self_report	stomach_cancer_self_report	1999-2000	questionnaire	disease	NA
stomach_cancer_self_report	stomach_cancer_self_report	2001-2002	questionnaire	disease	NA
stomach_cancer_self_report	stomach_cancer_self_report	2003-2004	questionnaire	disease	NA
stomach_cancer_self_report	stomach_cancer_self_report	2005-2006	questionnaire	disease	NA
testis_cancer_self_report	testis_cancer_self_report	1999-2000	questionnaire	disease	NA
testis_cancer_self_report	testis_cancer_self_report	2001-2002	questionnaire	disease	NA
testis_cancer_self_report	testis_cancer_self_report	2003-2004	questionnaire	disease	NA
testis_cancer_self_report	testis_cancer_self_report	2005-2006	questionnaire	disease	NA
thyroid_cancer_self_report	thyroid_cancer_self_report	1999-2000	questionnaire	disease	NA
thyroid_cancer_self_report	thyroid_cancer_self_report	2001-2002	questionnaire	disease	NA
thyroid_cancer_self_report	thyroid_cancer_self_report	2003-2004	questionnaire	disease	NA
thyroid_cancer_self_report	thyroid_cancer_self_report	2005-2006	questionnaire	disease	NA
uterine_cancer_self_report	uterine_cancer_self_report	1999-2000	questionnaire	disease	NA
uterine_cancer_self_report	uterine_cancer_self_report	2001-2002	questionnaire	disease	NA
uterine_cancer_self_report	uterine_cancer_self_report	2003-2004	questionnaire	disease	NA
uterine_cancer_self_report	uterine_cancer_self_report	2005-2006	questionnaire	disease	NA
other_cancer_self_report	other_cancer_self_report	1999-2000	questionnaire	disease	NA
other_cancer_self_report	other_cancer_self_report	2001-2002	questionnaire	disease	NA
other_cancer_self_report	other_cancer_self_report	2003-2004	questionnaire	disease	NA
other_cancer_self_report	other_cancer_self_report	2005-2006	questionnaire	disease	NA
any_cancer_self_report	Any cancer or malignancy?	1999-2000	questionnaire	disease	NA
any_cancer_self_report	Any cancer or malignancy?	2001-2002	questionnaire	disease	NA
any_cancer_self_report	Any cancer or malignancy?	2003-2004	questionnaire	disease	NA
any_cancer_self_report	Any cancer or malignancy?	2005-2006	questionnaire	disease	NA
LBDHDL	Direct HDL-Cholesterol (mg/dL)	2003-2004	laboratory	biochemistry	NA
any_family_cad	Any family with heart attack or angina?	1999-2000	questionnaire	disease	NA
any_family_cad	Any family with heart attack or angina?	2001-2002	questionnaire	disease	NA
any_family_cad	Any family with heart attack or angina?	2003-2004	questionnaire	disease	NA
any_family_cad	Any family with heart attack or angina?	2005-2006	questionnaire	disease	NA
current_past_smoking	Current or Past Cigarette Smoker?	1999-2000	questionnaire	smoking behavior	0,1,2
current_past_smoking	Current or Past Cigarette Smoker?	2001-2002	questionnaire	smoking behavior	0,1,2
current_past_smoking	Current or Past Cigarette Smoker?	2003-2004	questionnaire	smoking behavior	0,1,2
current_past_smoking	Current or Past Cigarette Smoker?	2005-2006	questionnaire	smoking behavior	0,1,2
occupation	occupation (never, blue-semi, blue-high, white-semi, white-high)	1999-2000	questionnaire	occupation	1,2,3,4,5
occupation	occupation (never, blue-semi, blue-high, white-semi, white-high)	2001-2002	questionnaire	occupation	1,2,3,4,5
occupation	occupation (never, blue-semi, blue-high, white-semi, white-high)	2003-2004	questionnaire	occupation	1,2,3,4,5
LBXVID	Vitamin D (ng/mL)	2005-2006	laboratory	nutrients	NA
DXXLSBMD	Lumber Spine BMD (g/cm^2)	1999-2000	examination	body measures	NA
DXXPEBMD	Lumber Pelvis BMD (g/cm^2)	1999-2000	examination	body measures	NA
DXXHEBMD	Head BMD (g/cm^2)	1999-2000	examination	body measures	NA
DXDTRLE	Trunk Lean excl BMC (g)	1999-2000	examination	body measures	NA
DXXTRFAT	Trunk Fat (g)	1999-2000	examination	body measures	NA
DXDTOLE	Total Lean excl BMC (g)	1999-2000	examination	body measures	NA
DXDTOFAT	Total Fat (g)	1999-2000	examination	body measures	NA
DXDTOBMD	Total BMD (g/cm^2)	1999-2000	examination	body measures	NA
DXXLSBMD	Lumber Spine BMD (g/cm^2)	2001-2002	examination	body measures	NA
DXXPEBMD	Lumber Pelvis BMD (g/cm^2)	2001-2002	examination	body measures	NA
DXDTRLE	Trunk Lean excl BMC (g)	2001-2002	examination	body measures	NA
DXXTRFAT	Trunk Fat (g)	2001-2002	examination	body measures	NA
DXDTOLE	Total Lean excl BMC (g)	2001-2002	examination	body measures	NA
DXDTOFAT	Total Fat (g)	2001-2002	examination	body measures	NA
DXDTOBMD	Total BMD (g/cm^2)	2001-2002	examination	body measures	NA
DXXLSBMD	Lumber Spine BMD (g/cm^2)	2003-2004	examination	body measures	NA
DXXPEBMD	Lumber Pelvis BMD (g/cm^2)	2003-2004	examination	body measures	NA
DXDTRLE	Trunk Lean excl BMC (g)	2003-2004	examination	body measures	NA
DXDTOLE	Total Lean excl BMC (g)	2003-2004	examination	body measures	NA
DXDTOFAT	Total Fat (g)	2003-2004	examination	body measures	NA
DXDTOBMD	Total BMD (g/cm^2)	2003-2004	examination	body measures	NA
DXXLSBMD	Lumber Spine BMD (g/cm^2)	2005-2006	examination	body measures	NA
DXXPEBMD	Lumber Pelvis BMD (g/cm^2)	2005-2006	examination	body measures	NA
DXDTRLE	Trunk Lean excl BMC (g)	2005-2006	examination	body measures	NA
DXXTRFAT	Trunk Fat (g)	2005-2006	examination	body measures	NA
DXDTOLE	Total Lean excl BMC (g)	2005-2006	examination	body measures	NA
DXDTOFAT	Total Fat (g)	2005-2006	examination	body measures	NA
DXDTOBMD	Total BMD (g/cm^2)	2005-2006	examination	body measures	NA
DXXHEBMD	Head BMD (g/cm^2)	2001-2002	examination	body measures	NA
DXXHEBMD	Head BMD (g/cm^2)	2003-2004	examination	body measures	NA
DXXHEBMD	Head BMD (g/cm^2)	2005-2006	examination	body measures	NA
occupation	occupation (never, blue-semi, blue-high, white-semi, white-high)	2005-2006	questionnaire	occupation	1,2,3,4,5
current_asthma	Current asthma?	1999-2000	questionnaire	disease	NA
current_asthma	Current asthma?	2001-2002	questionnaire	disease	NA
current_asthma	Current asthma?	2003-2004	questionnaire	disease	NA
current_asthma	Current asthma?	2005-2006	questionnaire	disease	NA
ever_asthma	Ever asthma?	1999-2000	questionnaire	disease	NA
ever_asthma	Ever asthma?	2001-2002	questionnaire	disease	NA
ever_asthma	Ever asthma?	2003-2004	questionnaire	disease	NA
ever_asthma	Ever asthma?	2005-2006	questionnaire	disease	NA
ever_arthritis	Ever arthritis?	1999-2000	questionnaire	disease	NA
ever_arthritis	Ever arthritis?	2001-2002	questionnaire	disease	NA
ever_arthritis	Ever arthritis?	2003-2004	questionnaire	disease	NA
ever_arthritis	Ever arthritis?	2005-2006	questionnaire	disease	NA
ever_rheumatoid_arthritis	Ever rheumatoid arthritis?	1999-2000	questionnaire	disease	NA
ever_rheumatoid_arthritis	Ever rheumatoid arthritis?	2001-2002	questionnaire	disease	NA
ever_rheumatoid_arthritis	Ever rheumatoid arthritis?	2003-2004	questionnaire	disease	NA
ever_rheumatoid_arthritis	Ever rheumatoid arthritis?	2005-2006	questionnaire	disease	NA
ever_osteo_arthritis	Ever osteo arthritis?	1999-2000	questionnaire	disease	NA
ever_osteo_arthritis	Ever osteo arthritis?	2001-2002	questionnaire	disease	NA
ever_osteo_arthritis	Ever osteo arthritis?	2003-2004	questionnaire	disease	NA
ever_osteo_arthritis	Ever osteo arthritis?	2005-2006	questionnaire	disease	NA
TELOMEAN	Mean Telomere Length	1999-2000	laboratory	aging	NA
TELOMEAN	Mean Telomere Length	2001-2002	laboratory	aging	NA
The data dictionary (also available in Data Citation 1 as ‘VarDescription.csv’) describes the column name of the variable (‘var’), the human readable description (‘var_desc’), module, series (year of survey), category, categorical levels of each variable in the dataset.					
